# Hydrate/moisture co-assisted synthesis enables humid-air stability of halide solid-state electrolytes

**DOI:** 10.1093/nsr/nwag209

**Published:** 2026-04-22

**Authors:** Xiangzhen Zhu, Chao Liu, Xiaolong Yan, Junyi Yue, Mingying Zhang, Simeng Zhang, Yueyue Wang, Han Wu, Yue Gong, Yanlong Wu, Xinmiao Wang, Shengjie Xia, Shanshan Wang, Zaifa Wang, Changtai Zhao, Jianwen Liang, Songbai Han, Xueliang Sun, Xiaona Li

**Affiliations:** Eastern Institute for Advanced Study, Zhejiang Key Laboratory of All-Solid-State Battery, Ningbo Key Laboratory of All-Solid-State Battery, Eastern Institute of Technology, Ningbo Institute of Digital Twin, Ningbo 315200, China; Solid State Batteries Research Center, GRINM (Guangdong) Institute for Advanced Materials and Technology, Foshan Key Laboratory of Advanced Electrochemical Functional Materials and Technology, Foshan 528051, China; Solid State Batteries Research Center, GRINM (Guangdong) Institute for Advanced Materials and Technology, Foshan Key Laboratory of Advanced Electrochemical Functional Materials and Technology, Foshan 528051, China; Solid State Batteries Research Center, GRINM (Guangdong) Institute for Advanced Materials and Technology, Foshan Key Laboratory of Advanced Electrochemical Functional Materials and Technology, Foshan 528051, China; Eastern Institute for Advanced Study, Zhejiang Key Laboratory of All-Solid-State Battery, Ningbo Key Laboratory of All-Solid-State Battery, Eastern Institute of Technology, Ningbo Institute of Digital Twin, Ningbo 315200, China; Eastern Institute for Advanced Study, Zhejiang Key Laboratory of All-Solid-State Battery, Ningbo Key Laboratory of All-Solid-State Battery, Eastern Institute of Technology, Ningbo Institute of Digital Twin, Ningbo 315200, China; Eastern Institute for Advanced Study, Zhejiang Key Laboratory of All-Solid-State Battery, Ningbo Key Laboratory of All-Solid-State Battery, Eastern Institute of Technology, Ningbo Institute of Digital Twin, Ningbo 315200, China; Solid State Batteries Research Center, GRINM (Guangdong) Institute for Advanced Materials and Technology, Foshan Key Laboratory of Advanced Electrochemical Functional Materials and Technology, Foshan 528051, China; Eastern Institute for Advanced Study, Zhejiang Key Laboratory of All-Solid-State Battery, Ningbo Key Laboratory of All-Solid-State Battery, Eastern Institute of Technology, Ningbo Institute of Digital Twin, Ningbo 315200, China; Solid State Batteries Research Center, GRINM (Guangdong) Institute for Advanced Materials and Technology, Foshan Key Laboratory of Advanced Electrochemical Functional Materials and Technology, Foshan 528051, China; Solid State Batteries Research Center, GRINM (Guangdong) Institute for Advanced Materials and Technology, Foshan Key Laboratory of Advanced Electrochemical Functional Materials and Technology, Foshan 528051, China; Eastern Institute for Advanced Study, Zhejiang Key Laboratory of All-Solid-State Battery, Ningbo Key Laboratory of All-Solid-State Battery, Eastern Institute of Technology, Ningbo Institute of Digital Twin, Ningbo 315200, China; Eastern Institute for Advanced Study, Zhejiang Key Laboratory of All-Solid-State Battery, Ningbo Key Laboratory of All-Solid-State Battery, Eastern Institute of Technology, Ningbo Institute of Digital Twin, Ningbo 315200, China; Solid State Batteries Research Center, GRINM (Guangdong) Institute for Advanced Materials and Technology, Foshan Key Laboratory of Advanced Electrochemical Functional Materials and Technology, Foshan 528051, China; Solid State Batteries Research Center, GRINM (Guangdong) Institute for Advanced Materials and Technology, Foshan Key Laboratory of Advanced Electrochemical Functional Materials and Technology, Foshan 528051, China; Solid State Batteries Research Center, GRINM (Guangdong) Institute for Advanced Materials and Technology, Foshan Key Laboratory of Advanced Electrochemical Functional Materials and Technology, Foshan 528051, China; Solid State Batteries Research Center, GRINM (Guangdong) Institute for Advanced Materials and Technology, Foshan Key Laboratory of Advanced Electrochemical Functional Materials and Technology, Foshan 528051, China; Shenzhen Key Laboratory of Solid State Batteries, Institute of Major Scientific Facilities for New Materials, Southern University of Science and Technology, Shenzhen 518055, China; Eastern Institute for Advanced Study, Zhejiang Key Laboratory of All-Solid-State Battery, Ningbo Key Laboratory of All-Solid-State Battery, Eastern Institute of Technology, Ningbo Institute of Digital Twin, Ningbo 315200, China; Eastern Institute for Advanced Study, Zhejiang Key Laboratory of All-Solid-State Battery, Ningbo Key Laboratory of All-Solid-State Battery, Eastern Institute of Technology, Ningbo Institute of Digital Twin, Ningbo 315200, China

**Keywords:** low-cost, halide solid-state electrolyte, stability in humid-air, all-solid-state batteries

## Abstract

Halide solid-state electrolytes (HSSEs) combine high ionic conductivity with wide electrochemical stability windows, making them promising candidates for all-solid-state lithium batteries (ASSLBs). However, their poor humid-air stability demands ultra-dry processing environments, severely limiting industrial scalability. Here, we report a water-assisted synthesis strategy to construct a zirconium-based core-shell structured HSSE, Li_2_Zr_1.5_OCl_6_@Li_2_CO_3_ (LZOC-H), under industrially viable dry-room conditions (dew point <−40 °C). By exploiting trace ambient H_2_O and CO_2_ during synthesis, a self-derived Li_2_CO_3_-rich layer is formed *in situ*, significantly enhancing air stability. The resulting LZOC-H electrolyte achieves a relatively high room-temperature ionic conductivity of 1.12 mS cm^−1^ and excellent moisture resistance. Full cell (Ni89|LZOC-H|LPSC|Li–In) shows an initial capacity of 200.4 mAh g^−1^ and retains 93.5% capacity over 1000 cycles at 1 C. Moreover, a pouch cell with a silicon anode fabricated in a dry room demonstrates stable cycling (85.1% retention over 300 cycles). This work offers a scalable and rare-earth-metal-free pathway for producing moisture-resistant HSSEs, addressing key challenges in ASSLBs’ commercialization.

## Teaser

A water-assisted technique builds a self-protective layer around a low-cost zirconium-based solid-state electrolyte. This enables robust all-solid-state batteries to be manufactured in dry-room environments, removing a major barrier to commercialization.

## INTRODUCTION

The commercial deployment of all-solid-state lithium batteries (ASSLBs) is anticipated to be accelerated by the development of halide solid-state electrolytes (HSSEs), which have garnered increasing interest from researchers in recent years owing to their high ionic conductivity (>1.0 mS cm^−1^ at 25°C), high voltage oxidation resistance (>4.2 V vs. Li^+^/Li), and good deformability [1–8]. Nonetheless, the industrial implementation of ASSLBs must consider economic issues, particularly the expenses associated with solid-state electrolytes (SSEs), including raw material costs, energy usage during manufacture, and application processes [9–11]. The current HSSEs face two critical challenges that hinder commercial viability. First, the predominance of the HSSEs’ reliance on rare-earth or precious metal element constituents such as indium (In) [12,13], yttrium (Y) [1], scandium (Sc) [14], and tantalum (Ta) [15–17] has driven raw material costs beyond sustainable thresholds for mainstream market adoption. Second, a critical gap exists in current research regarding the humid-air stability of HSSEs, which exhibit insufficient humid-air stability [12,18–20]. Notably, nearly all existing protocols for HSSEs study, manufacturing, and implementation require exceptionally stringent environmental controls, requiring ar-filled gloveboxes maintaining ultra-dry conditions [H_2_O/O_2_ < 0.01 ppm, dew point (DP) < −80°C, relative humidity (RH) < 3.2 × 10^−5^%] to ensure material stability during processing and testing. As a consequence, the manufacturing costs of the HSSEs experienced a substantial increase [21].

Zirconium (Zr)-based HSSEs have garnered significant attention as promising candidates for large-scale commercial adoption, owing to their cost-effective raw materials and enhanced air stability compared to conventional HSSEs [22–28]. In comparison to the precursors of the majority of HSSEs, the Zr element possesses significant reserves in the Earth’s crust. Furthermore, based on the hard and soft acid-base theory [29,30], while Zr^4+^ is categorized as a hard acid, its large ionic radius (0.73 Å) and high polarization capability result in a weaker binding affinity with O^2−^ of H_2_O (a hard base) compared to other transition metal elements such as Al^3+^, Ti^4+^, Nb^5+^, and Ta^5+^. Therefore, compared with other HSSEs, Zr-based HSSEs exhibit better stability against humid air [22]. Nevertheless, the moisture resistance of Zr-based HSSEs remains inadequate for the humidity requirements of contemporary industrial lithium-ion battery (LIB) production (DP < −40°C, RH < 0.6%), and their synthesis and application processes still cannot be conducted outside of a glovebox environment with low water content [19,20]. Consequently, there is a pressing demand for innovative techniques to enhance the humid-air stability of HSSEs and tackle the essential obstacles hindering their widespread commercialization in ASSLBs [28,31].

In this work, inspired by the ionization equilibrium principles of multicomponent weak acids [32,33], we successfully synthesized a moisture-stable, core-shell structured Li_2_Zr_1.5_OCl_6_@Li_2_CO_3_ (LZOC-H, ʻH’ means H_2_O-assisted) HSSE with a relatively high ionic conductivity of 1.12 mS cm^−1^ at 25°C by taking advantage of the moisture-absorbing site decomposition characteristics of HSSEs. Notably, even after being exposed to industrial dry-room conditions for 24 h, LZOC-H HSSE maintains more than 60% of its original ionic conductivity and structural integrity, indicating exceptional compatibility with scalable manufacturing environments while maintaining crucial electrochemical performance. Combining these characteristics, a full cell (LiNi_0.89_Co_0.06_Mn_0.05_O_2_(Ni89)|LZOC-H|Li_6_PS_5_Cl(LPSC)|Li–In) constructed within an Ar-filled glovebox demonstrates an initial discharge capacity of 200.4 mAh g^−1^ at 0.1 C, exhibiting remarkable cycle longevity with a capacity retention of 92.8% of its initial specific capacity (177.9 mAh g^−1^) after 1000 cycles at 1 C. Even after 24 h of exposure to a dry room, the LZOC-H-based ASSLB exhibits a stable discharge specific capacity of 196.3 mAh g^−1^ at 0.1 C and retains 84.6% of its initial specific capacity (133.5 mAh g^−1^) after 500 cycles at 1 C, demonstrating no decline in cycle performance. Moreover, when adapted to industrially pertinent configurations, a single-layer pouch cell with a Si anode (Ni89|LZOC-H|LPSC|Si), produced in the dry room, has an initial discharge capacity of 196.1 mAh g^−1^ at 0.05 C and maintains 85.1% capacity after 300 cycles at 0.5 C. This study presents an innovative methodology to produce humidity-resistant HSSEs with fast ion transport kinetics, addressing critical challenges in practical application and facilitating the transition to commercial ASSLBs. Specifically, during the synthesis process, it can neutralize atmospheric CO_2_, thereby mitigating emissions and contributing to the attainment of carbon neutrality objectives.

## RESULTS AND DISCUSSION

### Synthesis of LZOC-based HSSEs

**Figure 1.**
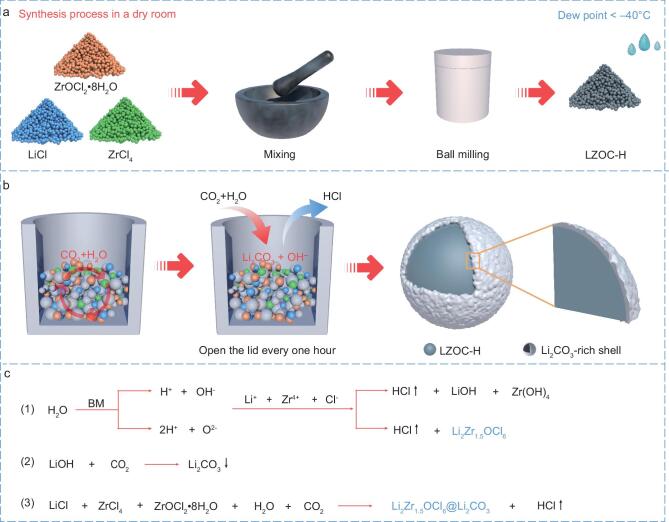
Schematic diagram of the material synthesis technology route for LZOC HSSEs. (a) The synthesis process of Zr-based HSSEs coated with a self-derived interfacial layer, which is conducted in a dry room. (b and c) The growth mechanisms of the LZOC electrolyte and self-derived interfacial layer.

Conventional HSSEs’ synthesis necessitates an Ar-filled glovebox (H_2_O/O_2_ < 0.01 ppm, DP < −80°C) and mechanochemical ball-milling due to extreme moisture sensitivity (Fig. S1). This stringent inert-atmosphere requirement severely limits scalability and hinders commercial deployment. To overcome this constraint, we developed an unconventional synthesis strategy enabling HSSE preparation in a dry room (DP < −40°C). By accurately controlling the stoichiometry of precursor materials and employing advanced synthesis methods within a dry chamber environment, we demonstrate the effectiveness of the process using Zr-based HSSEs, selected for their elemental abundance and cost-effectiveness (Tables S1–S3) [9,22]. As shown in Fig. 1a, ZrOCl_2_·8H_2_O (with coordinated water), ZrCl_4_, and LiCl were mixed under controlled dry-room conditions and subjected to high-energy ball-milling. The sealed milling jar thus contained crystalline water from precursors alongside trace atmospheric H_2_O and CO_2_ (Fig. 1b). During the ball-milling process, mechanical energy induces the gradual dissociation and rearrangement of various elements within the raw materials, and H_2_O molecules may progressively disintegrate into H^+^ and O^2−^ ions. Liberated O^2–^ ions incorporated into the lattice, forming a Li_2_Zr_1.5_OCl_6_ bulk phase. Concurrently, H^+^ reacted with Cl^–^ to form HCl gas, while residual H_2_O dissociation generated H^+^ and OH^–^ (Fig. 1c). Surface-adsorbed OH^–^ then reacted with CO_2_ to yield a Li_2_CO_3_ coating on electrolyte particles as HCl evolved. This process produced LZOC-H, a core-shell composite HSSE. We anticipate that LZOC-H will demonstrate improved stability in humid air and robust ionic transport. Li_2_CO_3_, which is frequently utilized as a solid electrolyte interphase film in LIBs, exhibits moderate ionic conductivity and high moisture resistance [34]. Critically, this method enables stable HSSE synthesis within commercial LIB-manufacturing environments, significantly reducing energy consumption and lowering barriers to commercialization.

### Structural analysis of LZOC HSSE

**Figure 2.**
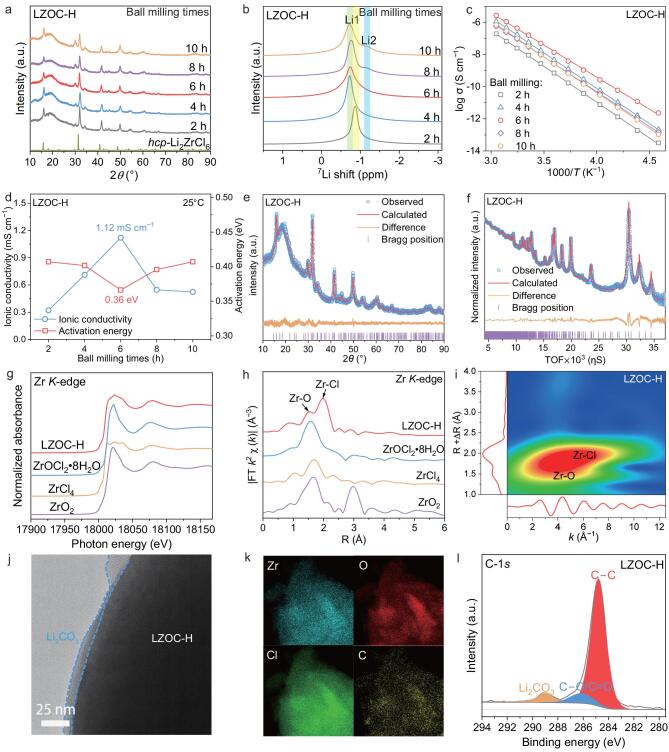
Structural and ionic transport characterization of LZOC-based electrolyte. (a) X-ray diffraction (XRD) patterns, (b) nuclear magnetic resonance (NMR) patterns, and (c) Arrhenius plots of LZOC-H electrolytes synthesized with different ball-milling times. (d) Trends of ionic conductivity and activation energy of LZOC-H electrolyte with different ball-milling times. Refinement for the (e) XRD and (f) neutron powder diffraction (NPD) data of LZOC-H electrolyte. (g) The X-ray absorption near-edge structure (XANES) and (h) Fourier-transformed extended X-ray absorption fine structure (FT-EXAFS) spectrum of the LZOC-H electrolyte at the Zr *K*-edge, with ZrOCl_2_·8H_2_O, ZrO_2_, and ZrCl_4_ as reference samples. (i) Wavelet transform (WT)-EXAFS contour plots of LZOC-H electrolyte at Zr-edge. The original EXAFS signal *χ*(*k*) is weighted by *k*^2^, and *k* represents wavenumber. *R* represents the radial distance. (j) High-resolution transmission electron microscopy (HRTEM) and (k) energy-dispersive X-ray spectroscopy (EDS) mapping images of the LZOC-H electrolyte. (l) C-1*s* X-ray photoelectron spectroscopy (XPS) spectrum of the LZOC-H electrolyte.

To identify the optimal synthesis conditions, we prepared a series of LZOC-H samples by varying the ball-milling time while keeping the ball-to-powder ratio, ball-milling speed, and environmental conditions constant. Figure 2a presents the X-ray diffraction (XRD) patterns of the resulting samples synthesized at different ball-milling durations. Notably, after just 2 h of ball-milling, the sample exhibited high crystallinity and a phase consistent with hexagonal close-packed Li_2_ZrCl_6_, with no detectable impurity peaks originating from the raw materials, including LiCl (Fig. S2), ZrCl_4_ (Fig. S3), or ZrOCl_2_·8H_2_O (Fig. S4). This indicates that the high-energy ball-milling process effectively drives the dissociation and recombination of precursors into a new, single-phase compound. Extending the milling time from 2 to 10 h led to only subtle changes in peak intensities, without any shift in peak positions or emergence of secondary phases, suggesting that prolonged milling does not significantly alter the bulk crystal structure. Upon opening the milling jar in the dry room, a pronounced increase in HCl gas concentration was observed (Fig. S5), which can be attributed to the reaction between H^+^ ions released from coordinated water in the precursors and Cl^−^ ions. According to the Lewis acid–base theory, this release of H^+^ ions results in the formation of HCl, while some O^2−^ is incorporated into the electrolyte lattice, and residual OH^–^ groups remain adsorbed on the particle surfaces.

Figure 2b displays the ^7^Li magic-angle spinning solid-state nuclear magnetic resonance (SS-NMR) spectra of LZOC‑H powders ball-milled for 2–10 h. As ball-milling time increases, the principal Li1 site resonance progressively shifts downfield from −0.86 to −0.73 ppm, reflecting gradual lattice disruption and the onset of local structural disorder. After 6 h, a new resonance appears at −1.2 ppm, which we assign to Li⁺ in the Li2 site within an increasingly amorphous, shielding-rich environment; its intensity grows with further ball-milling time, consistent with progressive amorphization. Deconvolution of each spectrum (Fig. S6) reveals two Gaussian components at the Li1 site: a narrow peak attributable to fast-exchanging Li⁺ and a broader peak corresponding to slower, more constrained Li⁺ [31,35–37]. With increasing ball-milling time, the fast-exchange component gradually intensifies and reaches a maximum at 6 h, whereas the slow-exchange component correspondingly diminishes. These trends suggest that 6 h of high-energy ball-milling strikes the optimal balance between structural disorder and the creation of conductive pathways, yielding the highest population of mobile Li1 sites. Whether the emerging amorphous Li2 phase further enhances bulk ion transport remains to be confirmed by complementary conductivity measurements.

The temperature-dependent ionic conductivities of LZOC-H samples synthesized with varying ball-milling durations were systematically evaluated via electrochemical impedance spectroscopy (EIS; Figs S7–S13 and Table S4). The room-temperature ionic conductivity increased steadily with ball-milling times, reaching a maximum of 1.12 mS cm^−1^ after 6 h (Fig. 2c and d). Further increase in ball-milling time led to a decline in conductivity, likely due to the formation of amorphous phase impurities that hindered Li^+^ transport. This trend was mirrored in the Li^+^ migration activation energy, which decreased with ball-milling time and reached a minimum of 0.36 eV at 6 h (Fig. 2d). To further optimize the synthesis, the ball-milling parameters, synthesis environment, and raw material composition were kept constant, except for bound water, while the proportion of the hydrated oxygen source was systematically varied. The resulting structural and electrochemical properties were characterized using XRD (Fig. S14), SS-NMR (Figs S15 and S16), ionic conductivity measurements, and activation energy analysis (Fig. S17). The optimal composition was identified at a LiCl:ZrCl_4_:ZrOCl_2_·8H_2_O molar ratio of 18:12.5:1, delivering the highest ionic conductivity (1.12 mS cm^1^) and the lowest activation energy (0.36 eV) for Li^+^ migration. Accordingly, the 6 h milled sample was selected for subsequent detailed structural and electrochemical analyses, and the electronic conductivity of the optimized LZOC-H sample was measured to be 1.29 × 10^−8^ S cm^−1^ (Fig. S18), confirming its excellent ionic selectivity and suitability for solid-state applications.

Scanning electron microscopy (SEM; Fig. S19) revealed particle sizes ranging from 1 to 20 μm. Elemental mapping by energy-dispersive X-ray spectroscopy (EDS; Fig. S20) confirmed the uniform distribution of Zr, O, and Cl throughout the LZOC-H particles. Rietveld refinement of the XRD pattern for the LZOC-H electrolyte (Fig. 2e) yielded satisfactory agreement factors (*R*_wp_ = 4.62%, *R*_p_ = 3.50%, Table S5), verifying phase purity and structural consistency with the targeted Li_3_YCl_6_-type framework with space group *P*${\bar{3}}$*m1* (PDF#44-0286) [1,22], and refinement details, including atomic coordinates, isotropic displacement parameters, and lattice parameters, are summarized in Table S6. Neutron powder diffraction (NPD) analysis (Fig. 2f, *R*_wp_ = 4.62%, *R*_p_ = 3.50%, Table S7) further confirmed successful incorporation of O into the 6i site, with an occupancy of 0.045, yielding an LZOC-H phase structurally analogous to Li_3_YCl_6_ (Table S8). As in LZOC-H, two distinct Li sites were identified: Li1 at the tetrahedral 6g site (occupancy = 0.503) and Li2 at the octahedral 6h site (occupancy = 0.731). A crystal structural diagram was displayed in Fig. S21. The powder XRD and NPD patterns of polycrystalline LZOC-H prove the single-phase nature of this sample.

To elucidate the local atomic environment of the LZOC-H HSSE, we employed X-ray absorption near-edge structure (XANES) spectroscopy at the Zr *K*-edge. This technique provides insights into the correlation between local chemical structure and Li⁺ transport properties within crystalline domains, as the spectral line shape at the *K*-edge of transition metals is highly sensitive to bond lengths and the symmetry of the surrounding coordination environment. As shown in Fig. 2g, the Zr *K*-edge XANES spectrum of LZOC-H (red trace) is compared with those of well-characterized reference compounds, including ZrCl_4_, ZrO_2_, and ZrOCl_2_·8H_2_O. The main peak positions of Zr are all around 18 021 eV, indicating that the valence state of zirconium in LZOC-H is Zr^4+^ [27,38]. The smooth and integrated spectral profile of LZOC-H indicates a more homogeneous local bonding environment. This observation suggests that O incorporation reduces bond length disparities within the Zr–Cl/O polyhedra [25,26], providing strong evidence for the partial substitution of Cl^−^ by O^2−^, a conclusion that is further supported by Rietveld refinement results from both XRD and NPD analyses. To further resolve the local coordination structure of Zr in LZOC-H, we performed Fourier-transformed extended X-ray absorption fine structure (FT-EXAFS) analysis at the Zr *K*-edge (Fig. 2h) [39,40]. Based on back-scattering paths derived from reference compounds of ZrCl_4_, ZrO_2_, and ZrOCl_2_·8H_2_O (Figs S22–S24), the major EXAFS features were assigned to Zr–O and Zr–Cl coordination shells. The coexistence of both Zr–O and Zr–Cl contributions confirms the formation of a mixed-anion environment around Zr, consistent with partial Cl^−^/O^2−^ substitution. This mixed-anion coordination structure is further supported by wavelet transform (WT)-EXAFS analysis (Fig. 2i) [41], which reveals Zr–O and Zr–Cl scattering contributions at approximately 1.5 and 2.0 Å, respectively. Quantitative EXAFS fitting (Fig. S25) indicates that each Zr atom is coordinated by approximately 1.8 O atoms at 2.07 Å and 5.9 Cl atoms at 2.48 Å (Table S9). These results collectively affirm that oxygen doping modifies the local Zr environment and promotes a more ordered and uniform configuration, which may enhance Li^+^ transport and aligns with our prior findings [26].

### Interphase analysis of LZOC-H HSSE

To gain deeper insights into the structural characteristics and interfacial chemistry of the LZOC-H solid electrolyte, we carried out high-resolution transmission electron microscopy (HRTEM) investigations. At lower magnification, HRTEM imaging revealed a uniform interfacial layer of ∼5 nm coating the surface of the LZOC-H particles (Fig. 2j), indicating a core-shell-like morphology. This layer was consistently observed across multiple particles, suggesting that the interfacial coating formed uniformly during the synthesis process. Upon further magnification, HRTEM imaging demonstrated that the surface region was amorphous in nature, lacking long-range atomic ordering, whereas the underlying bulk exhibited well-defined lattice fringes. Fast Fourier transform analysis of the HRTEM images confirmed the presence of distinct crystal planes in the core region of the particles. Specifically, the observed interplanar spacings corresponded to the (304), (602), and (700) planes of the refined LZOC-H crystal structure (Fig. S26), indicating relatively high crystallinity in the bulk phase. Additionally, the selected-area electron diffraction (SAED) pattern showed diffraction spots that could be indexed to the (001) plane of LZOC (Fig. S27), further validating the phase identity and confirming the polycrystalline nature of the bulk. Taken together, these structural analyses reveal that the LZOC-H material synthesized with crystallization water as a processing aid adopts a core-shell configuration, consisting of a polycrystalline LZOC-H core encapsulated by a uniformly distributed amorphous surface layer. To further probe the chemical composition of this interfacial region, we performed EDS mapping in TEM mode (Fig. 2j). The EDS maps (Fig. 2k) showed that the O element is homogeneously distributed throughout both the core and interfacial regions. Interestingly, the C element was also detected and appeared to be uniformly distributed across the particles. However, EDS spectral analysis (Fig. S28) revealed that the overall C element content was relatively low, suggesting that the C-containing species are confined primarily to the thin amorphous interfacial layer rather than incorporated into the crystalline bulk.

To further elucidate the surface chemical states and elemental composition of the LZOC-H electrolyte, X-ray photoelectron spectroscopy (XPS) analysis was carried out. The survey spectrum (Fig. S29) validates the existence of Li, Zr, O, Cl, and C, aligning with the expected elemental makeup of the material. We subsequently performed a fitting analysis on each peak spectrum to determine its underlying association and eliminate interference from carbon and oxygen pollution. High-resolution spectra were subsequently acquired for the C-1*s*, Li-1*s*, Zr-3*d*, O-1*s*, and Cl-2*p* regions to gain insight into the local chemical environments. The high-resolution C-1*s* spectrum (Fig. 2l) displays a dominant peak at 284.8 eV, corresponding to C–C bonds typically associated with adventitious carbon. A secondary peak at 286.1 eV can be attributed to C=O or C–O bonds, likely arising from physisorbed CO_2_ or organic species on the particle surface. Notably, an additional peak at 289.1 eV is assigned to the carbonate group (CO_3_^2−^), indicating the presence of surface carbonate species [34,42,43]. In the Li-1*s* spectrum (Fig. S30a), a main peak at 54.1 eV is assigned to Li^+^ within the bulk LZOC-H structure. A shoulder at 56.4 eV may be ascribed to LiCl, LiOH, or Li_2_CO_3_, as these compounds are closely related; thus, further investigation of their primary constituents is required by synthesizing the fitting findings of other elements [44–46]. The Zr-3*d* spectrum (Fig. S30b) displays two distinct peaks at 183.2 and 185.6 eV, attributed to the Zr-3*d*_5/2_ and Zr-3*d*_3/2_ spin-orbit components, respectively, confirming the Zr^4+^ oxidation state in LZOC-H, which aligns with the Zr *K*-edge XANES findings [47,48]. The O-1*s* spectrum (Fig. S30c) contains a primary peak at 530.7 eV, attributed to lattice oxygen. A secondary peak at 532.0 eV is assigned to surface hydroxyl species (OH^−^), or C–O of carbonate species (CO_3_^2−^), while a shoulder at 533.5 eV corresponds to C=O of CO_3_^2−^ or adsorbed H_2_O [49,50]. The Cl-2*p* region (Fig. S30d) displays a spin-orbit doublet at 198.6 and 200.4 eV, corresponding to Cl-2*p*_3/2_ and Cl-2*p*_1/2_, indicating the presence of covalently bonded chlorine within the structure [22,27]. The fitting results of the combined Zr-3*d* and Cl-2*p* spectra indicate a lack of overlap between the Cl-2*p*_3/2_ and Cl-2*p*_1/2_ peaks. Consequently, we hypothesize that the Cl element predominantly pertains to LZOC-H. Taken together, the XPS results corroborate the TEM analysis, confirming the formation of an amorphous surface layer enriched in Li_2_CO_3_ and OH^−^ species on LZOC-H particles.

For comparison, Li_2_Zr_1.5_OCl_6_-Ar (LZOC-A) was synthesized using the same raw material ratios and ball-milling conditions as those for LZOC-H, all of which were carried out in an argon atmosphere. Li_2_O was used as the oxygen source in the separate preparation of Li_2_ZrOCl_4_-Li_2_O (LZOC-O), and all processes were carried out in an argon environment in accordance with previous techniques [23]. Both samples were characterized by XRD, SEM, TEM, and EIS measurements. LZOC-A exhibited XRD diffraction peaks (Fig. S31) and particle sizes (Fig. S32) analogous to LZOC-H. However, TEM revealed no uniform interfacial protective layer, instead showing sparse plate-like amorphous regions (Fig. S33). Similarly, LZOC-O displayed low crystallinity (Fig. S34) and particle sizes of 1–10 μm (Fig. S35), with TEM confirming smooth particle surfaces devoid of any protective layer (Fig. S36). These results collectively demonstrate that crystalline water and moisture in a dry room are critical for constructing the interfacial protective layer during electrolyte synthesis. Additionally, to examine the effect of CO_2_ concentration on the interfacial layer during the synthesis of LZOC-H electrolyte, 0.5 g of dry ice was introduced into a sealed ball-milling jar before synthesis to create a CO_2_-rich experimental atmosphere. The incorporation of dry ice led to an augmented thickness of the amorphous layer (Fig. S37), but the ionic conductivity significantly diminished (Fig. S38), dropping below 0.3 mS cm^−1^.

### Humid-air stability analysis of LZOC electrolyte in a dry room

**Figure 3.**
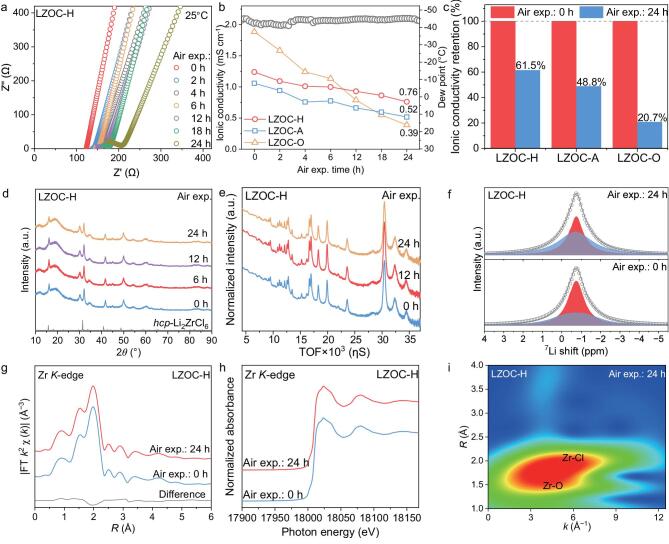
Humidity-air stability of LZOC-based HSSEs in a dry room (dew point (DP): −40°C) at 25°C. (a) Nyquist plots of LZOC-H electrolyte after different air-exposure times. (b) Ionic conductivity degradation kinetics and (c) ionic conductivity retention rate of LZOC-H, LZOC-A, and LZOC-O electrolytes under prolonged air exposure. Structural characterization of LZOC-H electrolyte: (d) XRD patterns, (e) NPD profiles, and (f) solid-state-NMR spectra before and after 24 h of air exposure. Local coordination environment analysis of LZOC-H electrolyte via Zr *K*-edge XANES spectra in (g) R space and (h) *k* space, and (i) WT-EXAFS contour plot of LZOC-H before and after 24 h of air exposure.

To verify the stability in humid air of the LZOC-H electrolyte, three electrolytes (LZOC-H, LZOC-A, and LZOC-O) were exposed to a dry room for 24 h. The temperature-dependent ionic conductivities of the three Zr-based halide electrolyte samples were investigated via EIS. The Nyquist plots of LZOC-H and the corresponding fitting data are shown in Fig. 3a, Figs S39–S46, and Table S10. Data for LZOC-A and LZOC-O are presented in Figs S47–S55, S56–S64, and Tables S11 and S12, respectively. Figure 3b and Table S13 illustrate the variation in ionic conductivity as a function of air-exposure time. The ionic conductivities diminished progressively with extended exposure to air at room temperature. Thanks to the high hydrolysis resistance, relatively low lithium-ion migration energy barrier (0.227–0.491 eV) [51], and high lithium-ion conductivity (∼6.7 × 10^−8^ S cm^−1^) [52] of Li_2_CO_3_ interphase, the LZOC-H HSSE exhibits outstanding water resistance while maintaining a relatively high ionic conductivity, marking a significant advancement in the development of SSEs that are stable in humid air. Notably, LZOC-H exhibited significantly slower degradation compared to LZOC-A and LZOC-O. After 24 h of air exposure, LZOC-H maintained 61.5% of its initial ionic conductivity. In contrast, LZOC-A and LZOC-O preserved only 48.8% and 20.7%, respectively (Fig. 3c). In addition, the lithium ion migration energy barriers calculated based on temperature-dependent EIS are shown in Figs S39, S48, and S57, respectively. The activation energies of the LZOC-H and LZOC-A electrolytes did not change significantly after 24 h of exposure to air (Figs S39b and S48b), while the activation energy of the LZOC-O electrolyte gradually increased with increasing exposure time (Fig. S57b). This indicates that the amorphous Li_2_CO_3_-rich interface layer of LZOC-H effectively resists moisture in the dry room, reducing the probability of moisture decomposition in the electrolyte body particles. Furthermore, we performed air stability exposure tests under the same conditions as a comparable study on previously documented established HSSEs, including Li_3_InCl_6_ (LIC) [13], LiTaOCl_4_ [16], and Li_2.6_Y_0.6_Hf_0.4_Cl_6_ [53]. The results demonstrated that following 24 h of exposure to air in the dry room, the retention of ionic conductivities was 44.5%, 12.3%, and 12.7%, respectively (Figs S65–S68). These results further underscore the remarkable humid-air stability of the synthesized LZOC-H electrolyte in the dry room. It is important to highlight that, in contrast to previous studies on the humid-air stability of HSSEs (Table S14), this study represents the inaugural validation of the LZOC-H electrolyte’s resilience to humid air under conditions typical of an industrial dry-room environment.

To assess the structural stability of the LZOC-H electrolyte under dry-room conditions <−40 °C, we conducted SEM imaging after 0, 6, 12, and 24 h of air exposure (Fig. S69). With prolonged exposure, the particle surfaces exhibited increased roughness (Fig. S69d), though the overall particle morphology remained largely unchanged. Elemental mapping via EDS confirmed a consistent distribution of Zr, O, and Cl over time (Fig. S70). Furthermore, *ex situ* XRD and NPD measurements (Fig. 3d and e) at various exposure durations revealed no discernible changes in the crystal structure or emergence of impurity phases, indicating robust structural integrity. Solid-state ^7^Li NMR spectroscopy (Fig. 3f), however, revealed a reduction in the peak area associated with fast Li-ion transport and a corresponding increase in the slow-ion transport signal after 24 h of exposure, correlating with a decline in ionic conductivity. Complementary Zr *K*-edge XANES, FT-EXAFS, and WT-EXAFS analyses (Fig. 3g–i, Figs S71 and S72, and Table S15) showed negligible changes in the local coordination environment of Zr atoms, further underscoring the structural resilience of LZOC-H under dry-room conditions.

In contrast, LZOC-A and LZOC-O electrolytes, devoid of a functional passivation layer, are immediately exposed to ambient H_2_O, making their particle surfaces particularly vulnerable to hydrolytic degradation. Consistent with previous studies, such direct moisture exposure initiates a sequence of irreversible chemical events, encompassing the hydrolysis of Zr–Cl bonds and the ensuing reconfiguration of the local coordination environment. These processes result in the generation of non-Li⁺-conductive or poorly conducting byproducts, including ZrOCl_2−_*_x_*·H_2_O, Li_2_ZrO_3_, and LiCl [19]. The build-up of insulating phases on particle surfaces and grain boundaries obstructs continuous Li^+^ transport channels and elevates interfacial resistance, resulting in significant deterioration of ionic conductivity and electrochemical performance when exposed to air.

### Interfacial evolution of LZOC-H electrolyte in a dry room

**Figure 4.**
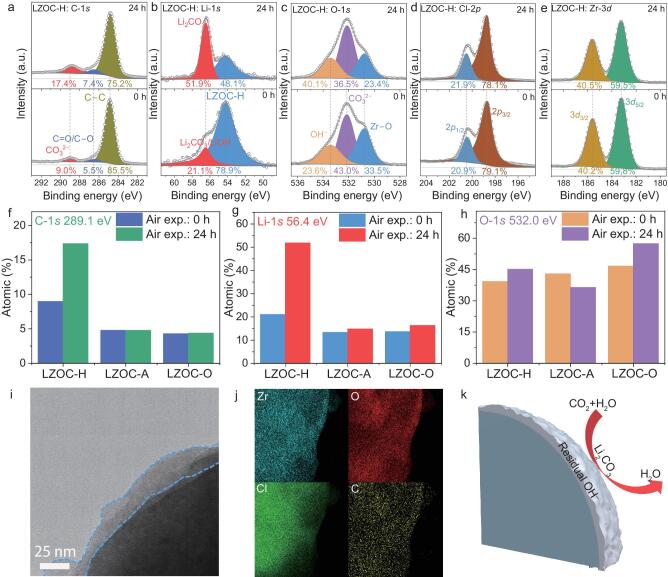
Interfacial evolution of LZOC HSSEs in a dry room. (a–e) XPS spectra of LZOC-H before and after humid-air exposure for 24 h. Evolution of XPS characteristic peaks of (f) C-1*s*, (g) Li-1*s*, and (h) O-1*s* related to Li_2_CO_3_ in LZOC-based electrolytes (LZOC-H, LZOC-A, and LZOC-O) before and after humid-air exposure. (i) HRTEM and (j) TEM-EDS images of the LZOC-H electrolyte after air exposure for 24 h. (k) Schematic diagram of the interfacial evolution of LZOC-H electrolyte during air exposure in a dry room.

To elucidate the origin of the diminished ionic conductivities observed in Zr-based electrolytes upon exposure to air in a dry room, *ex situ* XPS analyses were performed on three representative electrolytes before and after air exposure. Spectral fitting was carried out for the characteristic binding energy peaks of five elements. In the C-1*s* spectrum of LZOC-H (Fig. 4a), the binding energy peak corresponding to Li_2_CO_3_ at 289.1 eV accounted for 9.0% of the total peak area before exposure, increasing to 17.4% after 24 h. In the Li-1*s* spectrum (Fig. 4b), the LiOH/Li_2_CO_3_ peak at 56.4 eV showed a substantial increase in relative area, from 21.1% to 51.9%, while the signal attributed to the Li^+^ in the LZOC-H framework at 54.1 eV declined from 78.9% to 48.1%. Similarly, in the O-1*s* spectrum (Fig. 4c), the component associated with C=O/H_2_O at 533.5 eV decreased from 32.4% to 25.7%, whereas the peak assigned to C–O/O–H species at 532.0 eV increased from 39.3% to 45.8%. Notably, the peak area associated with lattice oxygen (Zr–O) remained stable. The Cl-2*p* and Zr-3*d* regions showed no significant variation in peak intensities or distributions, suggesting the preservation of the host lattice structure (Fig. 4d and e).

For comparison, XPS fitting results for LZOC-A and LZOC-O are presented in Figs. S73a–e and S73f–j, respectively. The relative changes in Li-1*s*, C-1*s*, and O-1*s* peak areas across the three electrolytes are summarized in Fig. 4f–h. These findings indicate that residual coordinated water and OH^−^ species in LZOC-H progressively react with atmospheric CO_2_ during air exposure (Fig. S74), leading to the formation and accumulation of Li_2_CO_3_, accompanied by the consumption of OH⁻ and H_2_O. HRTEM (Fig. 4i) and TEM–EDS (Fig. 4j and Fig. S75) analyses further reveal the growth of an amorphous surface layer to a thickness of 10–20 nm, which likely contributes to the observed decline in ionic conductivity, consistent with the XPS results. A schematic of the interfacial phase evolution process during dry-room exposure is illustrated in Fig. 4k. This mechanism ensures that, upon exposure to air, the electrolyte can mend regions where the particles are inadequately compacted, hence enhancing its resistance against moisture-induced degradation in the dry room.

Following 1-h exposure of LZOC samples to ambient air (RH > 30%), significant modifications were observed: alterations in the crystalline structure and volumetric properties of the electrolyte (XRD, Fig. S76), a marked reduction in chloride content occurred alongside a notable increase in carbon content (XPS, Fig. S77), the amorphous interfacial layer experienced substantial thickening (TEM, Fig. S78), and it transitioned into an amorphous/nanocrystalline composite phase (SAED, Fig. S79). The development of this amorphous Li_2_CO_3_ phase serves as the rate-limiting factor within the dry room. Furthermore, TEM–EDS mapping results (Fig. S80 and Table S16) reveal an exceedingly low chlorine concentration (mass ratio < 0.06%), implying that during sample preparation in ambient conditions, the LZOC-H electrolyte has experienced breakdown, resulting in HCl evolution. This discovery aligns with our hypothesis articulated in Fig. 1c. Once a complete interfacial layer is formed, it efficiently blocks moisture ingress, thereby preserving electrolyte stability for a certain period (e.g. 24 h). Nonetheless, under more extreme ambient air conditions (RH > 30%), this protective barrier does not adequately protect the underlying electrolyte matrix. This failure arises from the intrinsic Lewis acidity and electrophilic characteristics of metal ions in halide electrolytes, which expedite electrolyte dissolution. The ongoing production of hydroxide ions and the precipitation of hydrochloric acid result in a persistent elevation of Li_2_CO_3_ concentration. Thus, the production of Li_2_CO_3_ upon exposure to air is not a self-limiting phenomenon.

### Electrochemical performance of LZOC-based ASSLBs

**Figure 5.**
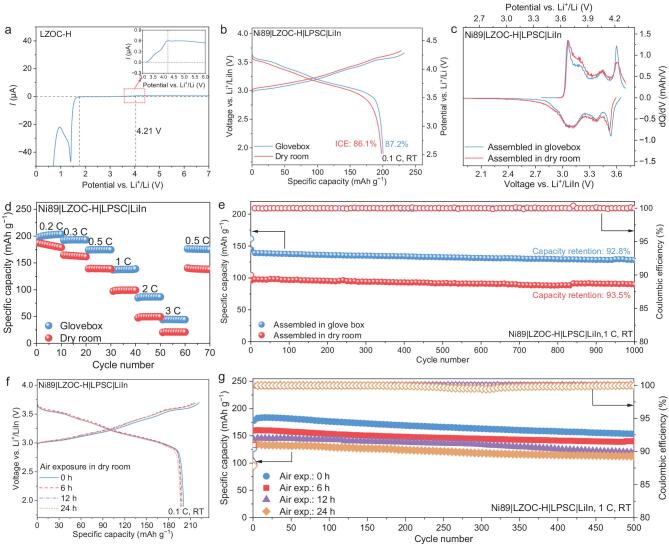
Electrochemical performance of Ni89|LZOC-H|LPSC|In–Li mold-type ASSLBs. (a) Electrochemical stability window of the LZOC-H HSSE determined by LSV at a scan rate of 0.1 mV s^−1^. (b) The charge–discharge curves and corresponding (c) d*Q*/d*V* profiles during the initial cycles at 0.1 C for mold cells assembled in glovebox and dry-room environments. (d) Rate capability comparison and (e) cyclic stability evaluation of these cells fabricated under different assembly environments (glovebox *vs.* dry room). (f) The charge–discharge curves and (g) long-term cycling performance of these cells assembled with LZOC-H HSSE after different times of exposure in a dry room.

The application potential of the LZOC-H solid electrolyte was evaluated in ASSLBs. Linear sweep voltammetry (LSV) measurements confirmed the exceptional high-voltage stability of LZOC-H, revealing an electrochemical oxidation window >4.2 V versus Li^+^/Li (Fig. 5a). Notably, even beyond its oxidation threshold, no severe oxidative decomposition occurred, indicating compatibility with high-voltage cathodes such as LiCoO_2_, LiNi_0.8_Co_0.1_Mn_0.1_O_2_ (NCM811), and LiNi_0.89_Co_0.55_Mn_0.55_O_2_ (Ni89; Figs S81 and S82). However, a reduction limit near 1.65 V suggests instability against lithium metal or indium anodes. To address this, we employed a lithium-indium (Li–In) alloy anode and inserted a Li_6_PS_5_Cl (LPSC; Figs S83 and S84) sulfide electrolyte buffer layer between the LZOC-H electrolyte and the Li–In anode. This configuration enabled the assembly of Ni89|LZOC-H|LPSC|Li–In mold-type ASSLBs for electrochemical performance evaluation. Mold-type ASSLBs were assembled in both an Ar-filled glovebox and a dry room. Initial charge–discharge curves at 0.1 C (Fig. 5b) showed comparable first-cycle Coulombic efficiencies (87.2% vs. 86.1%) and reversible specific capacities (200.4 vs. 198.0 mAh g^−1^) for cells assembled in the glovebox versus those assembled in the dry room. However, mold-type ASSLB assembled in the dry room exhibited slightly enhanced polarization, likely attributable to moisture exposure during electrode preparation and cell assembly. The d*Q*/d*V* curves derived from the 0.1 C test are illustrated in Fig. 5c. Mold-type ASSLBs assembled in varying environments exhibited comparable redox potentials, akin to the charge–discharge curves, with only minor discrepancies in polarization. This suggests that the assembly of batteries in a dry room does not result in any fundamental alterations to the electrode or electrolyte materials.

**Figure 6.**
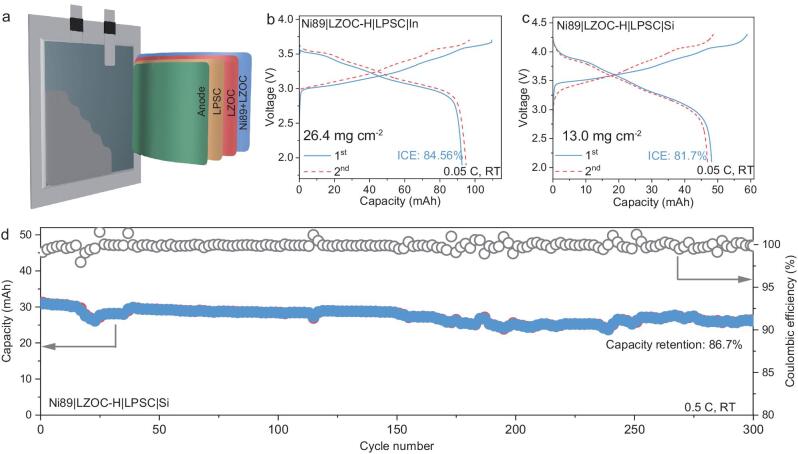
Electrochemical performance of pouch-type ASSLBs. (a) Schematic illustration of the all-solid-state pouch cell structure. (b) Charge–discharge voltage profiles of the Ni89|LZOC-H|LPSC|In pouch cell during the first two cycles at 0.1 C. (c) The first two cyclic charge–discharge curves of the Ni89|LZOC-H|LPSC|Si pouch cell measured at 0.05 C. (d) Cycling performance of the Ni89|LZOC-H|LPSC|Si pouch cell at 0.5 C.

The rate performance of the assembled mold-type ASSLB was evaluated at room temperature by progressively increasing the current density from 0.1 to 3 C. ASSLMBs fabricated in an Ar-filled glovebox delivered reversible discharge capacities of 200.4, 199.5, 192.7, 174.5, 137.5, 86.2, and 44.7 mAh g^−1^ at 0.1, 0.2, 0.3, 0.5, 1, 2, and 3 C, respectively (Fig. 5d and Fig. S85a). In contrast, cells assembled in a dry room exhibited reduced capacities of 198.0, 186.0, 164.4, 139.6, 98.6, 49.3, and 21.2 mAh g^−1^ at the corresponding current densities (Fig. 5d and Fig. S85b), owing to moisture-induced degradation of ionic transport kinetics. Notably, when the current density was reverted to 0.5 C, the glovebox-assembled cell retained nearly 100% of its initial 0.5 C capacity, while the dry room-assembled counterparts retained approximately 99.5%. Remarkably, both cells exhibited over 90.0% (92.8% vs. 93.5%) specific capacity retention following 1000 cycles at 1 C (Fig. 5e and Fig. S86), with an average Coulombic efficiency exceeding 99.90%. To further assess the HSSE’s humid-air stability, mold-type ASSLBs were assembled using the LZOC-H HSSE after controlled air exposure (0, 6, 12, and 24 h) in a dry room, followed by fabrication in a glovebox. As shown in Fig. 5f, these cells exhibited reversible specific capacities of 200.4, 197.0, 198.5, and 196.3 mAh g^−1^ at 0.1 C, respectively, demonstrating minimal variation in electrochemical performance and no observable increase in polarization. At a higher current density of 1 C after three cycles at 0.1C, a slight reduction in capacity was noted, which was attributed to diminished ionic transport kinetics upon prolonged humid-air exposure. Nevertheless, the long-term cycling stability remained largely unaffected (Fig. 5g and Fig. S87). After 500 cycles, the specific capacity retention relative to their initial specific capacity at 1 C was 86.3%, 87.6%, 83.6%, and 84.6% for 0, 6, 12, and 24 h of humid-air exposure, respectively, with an average Coulombic efficiency of 99.95%. By contrast, mold-type ASSLBs constructed with LZOC-A (Fig. S88a) and LZOC-O (Fig. S88b) HSSEs before air exposure deliver reversible specific capacities of 208.8 and 197.3 mAh g^‒1^, respectively, at 0.1 C, along with comparatively high initial Coulombic efficiencies of 90.9% and 91.1%. These results are analogous to those acquired for mold-type ASSLBs utilizing LZOC-H HSSE. Following 24 h of exposure in a dry room, the Coulombic efficiencies of the LZOC-A- and LZOC-O-based cells were rather stable, at 91.7% and 90.8%, respectively. The diminished ionic conductivity caused by air exposure resulted in heightened cell polarization, leading to decreased first-cycle reversible specific capacities of 191.4 and 164.3 mAh g^−1^. The capacity decline is significantly more pronounced than that seen in LZOC-H-based cells, regardless of the existence of a stable interfacial layer. When the current density was elevated to 1 C, the reversible specific capacities of the LZOC-A and LZOC-O cells diminished to 148.4 and 124.2 mAh g^−1^, respectively. Despite the losses in capacity, both electrolytes demonstrate remarkable cycle stability before and after exposure to air (Fig. S88c), attributable to the inherent breakdown behavior of LZOC-based electrolytes that does not lead to residual hydration. These results collectively further underscore the outstanding electrochemical performance and excellent air stability of the LZOC-H HSSE. To assess the practical applicability of the LZOC-H HSSE, we assembled two distinct pouch-type ASSLBs using indium (In) metal and nano-silicon (Si) as anode materials (Figs S89 and S90) [54], with fabrication conducted in a dry room (Fig. 6a). The charge–discharge profiles of the Ni89|LZOC-H|LPSC|In pouch-type ASSLB (Fig. S91) during the initial two cycles at 0.05 C are shown in Fig. 6b, delivering a reversible capacity of approximately 92.6 mAh, corresponding to a specific capacity of 197.2 mAh g^−1^ (Fig. S92), and a first-cycle Coulombic efficiency of 84.7%. However, the long-term cycling performance at 0.3 C is limited. As illustrated in Fig. S93, the battery exhibits a rapid decline in Coulombic efficiency, increasing polarization, and accelerated capacity fade after 20 cycles, primarily due to indium anode pulverization and aggressive lithium dendrite formation during repeated lithiation/delithiation.

In contrast, substituting the anode with nano-Si, a material of significantly greater commercial importance, leads to considerably improved electrochemical performance. The Ni89|LZOC-H|LPSC|Si pouch-type ASSLBs (Fig. S94) demonstrate consistent and reproducible electrochemical performance. Figure 6c illustrates that the first two formation cycles at 0.05 C yield a discharge capacity of 48.1 mAh, equating to a reversible specific capacity of 201.5 mAh g^−1^ (Fig. S95), accompanied by an initial Coulombic efficiency of 81.7%. The cell preserves over 86.7% of its initial capacity after 300 cycles at 0.5 C, while achieving an average Coulombic efficiency over 99.90% (Fig. 6d and Fig. S96). This degree of cycling stability in a pouch-cell configuration with a high-capacity Si anode is advantageous compared to numerous advanced halide-based ASSLBs, which frequently experience significant interfacial degradation, inadequate mechanical resilience to Si volume expansion, and demanding processing conditions under extremely dry environments. Furthermore, the LZOC-H HSSEs exhibit strong interfacial compatibility with both the cathode and the sulfide-based electrolyte, facilitating stable Li^+^ transport and highly reversible electrochemical processes during extended cycling. The capacity to maintain elevated Coulombic efficiency at modest current densities (0.5 C) in a scalable pouch-cell configuration underscores the inherent chemical and electrochemical stability of LZOC-H under realistically applicable operating circumstances. From a manufacturing standpoint, the utilization of economical raw materials and a scalable, water-assisted synthesis method further differentiates LZOC-H from traditional HSSEs, which generally depend on expensive precursors and moisture-sensitive processing. These advantages collectively mitigate numerous persistent obstacles in the commercialization of ASSLBs, such as interfacial instability, process complexity, and cost. LZOC-H presents itself as a highly promising high-safety solid electrolyte for next-generation high-energy-density ASSLBs, especially in large-format pouch cells aimed at electric vehicle and grid storage applications.

## CONCLUSION

This work tackles two critical obstacles hindering the commercialization of HSSEs, elevated material costs and inadequate moisture tolerance, through the rational design of a core-shell structured LZOC-H HSSE. Guided by ionization equilibrium principles, we harnessed the intrinsic moisture sensitivity of Zr-based HSSEs to trigger a controlled reaction with trace H_2_O and CO_2_ in industrial dry-room conditions (DP < −40°C), enabling *in situ* formation of a Li_2_CO_3_-rich passivation layer during synthesis. This self-derived reaction obviates the need for stringent Ar-filled glovebox processing (DP < −80 °C), offering a scalable and energy-efficient fabrication route. The resulting LZOC-H electrolyte exhibits a combination of high room-temperature ionic conductivity (1.12 mS cm^−1^) and enhanced humid-air stability, retaining over 60% of its conductivity after 24 h of exposure to dry-room conditions. Full cells incorporating LZOC-H (Ni89|LZOC-H|LPSC|Li–In) demonstrate excellent electrochemical durability, with 93.5% capacity retention over 1000 cycles at 1 C following ambient exposure, while industrially assembled pouch cells employing a Si anode maintain 85.1% capacity retention after 300 cycles at 0.5 C. The use of earth-abundant Zr avoids reliance on scarce rare-earth elements, while the self-derived Li_2_CO_3_-rich interface offers intrinsic protection against ambient degradation. This technique can also be applied to additional high-conductivity solid electrolytes (HSSEs), including Li_3_ScCl_6_, Li_3_YCl_6_, LIC, or LiMOCl_4_ (where M = Nb, Ta), providing a cost-effective solution for HSSE development. In conclusion, although more accurate data necessitate a comprehensive lifecycle assessment for commercial ASSLBs, the economic and environmental rationale for the LZOC-H synthesis is valuable. It intentionally circumvents the primary limitations of glovebox reliance, providing a viable avenue for the incorporation of high-performance HSSEs into the conventional battery industry.

## METHODS

The Li_2_Zr_1.5_OCl_6_-H_2_O (LZOC-H) solid electrolyte was prepared by ball-milling in a dry-room environment (DP ∼ −40°C, H_2_O ∼ 188 ppm, RH ∼0.6%). The precursors, comprising LiCl (Macklin, 99%), ZrCl_4_ (Macklin, 98%), and ZrOCl_2_·8H_2_O (Macklin, 98%), were meticulously weighed (0.2543, 0.9710, and 0.1074 g, respectively) in accordance with the stoichiometric molar ratios of the intended composition. They were then placed into a zirconia ball-milling jar (50 mL) holding 40 g of zirconia grinding balls. The dimensions of the balls and their respective weights were as follows: 3 mm (15 g), 5 mm (15 g), and 8 mm (10 g). Mechanical milling was performed at 600 rpm for various times (2, 4, 6, 8, and 10 h) using a FRITSCH, Pulverisette 7 premium line ball-mill machine. The LZOC-A was synthesized using identical stoichiometric ratios and milling parameters (600 rpm, 6 h) with LZOC-H, but processed in an Ar-filled glove box (H_2_O/O_2_ < 0.01 ppm). For LZOC-O preparation, 0.2988 g of Li_2_O (Macklin, 99%) and stoichiometrically calculated (2.3304 g) ZrCl_4_ (Macklin, 98%) were subjected to the same ball-milling procedure (600 rpm, 6 h) in a zirconia container in an Ar-filled glovebox (H₂O/O₂ < 0.01 ppm). The LPSC solid electrolyte required a two-step synthesis strategy in an Ar-filled glovebox. Stoichiometric amounts of Li_2_S (Alfa Aesar, 99.9%), P_2_S_5_ (Sigma-Aldrich, 99%), and LiCl (Sigma-Aldrich, 99%) were first subjected to planetary ball-milling at 550 rpm for 8 h. The resultant amorphous powder was then uniaxially pressed into dense pellets (12 mm diameter) under an Ar atmosphere and encapsulated in quartz ampoules for thermal processing. A controlled annealing protocol was implemented: heating to 550°C at 5°C min^−1^, followed by isothermal treatment for 5 h, with subsequent natural cooling to ambient temperature.

More detailed methods can be found in the online Supplementary file.

## Supplementary Material

nwag209_Supplemental_File
