# Supplementary material for: Hydrate/moisture co-assisted synthesis enables humid-air stability of halide solid-state electrolytes
Source: Natl Sci Rev. 2026 Apr 22;13(10):nwag209. doi: 10.1093/nsr/nwag209 (PMC13291819; doi:10.1093/nsr/nwag209)
Supplement: nwag209_Supplemental_File [file nwag209_Supplemental_File.pdf]

Supporting Information

Hydrate/moisture co-assisted synthesis enables humid-air stability of halide solid-state electrolytes

Xiangzhen Zhu[1,2], Chao Liu[2], Xiaolong Yan[2], Junyi Yue[1], Mingying Zhang[1], Simeng Zhang[1], Yueyue Wang[2], Han Wu[1], Yue Gong[2], Yanlong Wu[2], Xinmiao Wang[1], Shengjie Xia[1], Shanshan Wang[2], Zaifa Wang[2], Changtai Zhao[2], Jianwen Liang[2,], Songbai Han[3,]*, Xueliang Sun[1,]*, Xiaona Li[1,]**

[1] Eastern Institute for Advanced Study, Zhejiang Key Laboratory of All-Solid-State Battery, Ningbo Key Laboratory of All-Solid-State Battery, Eastern Institute of Technology, Ningbo Institute of Digital Twin, Ningbo, Zhejiang, 315200, P.R. China

[2] Solid State Batteries Research Center, GRINM (Guangdong) Institute for Advanced Materials and Technology, Foshan Key Laboratory of Advanced Electrochemical Functional Materials and Technology, Foshan, Guangdong 528051, P. R. China.

[3] Shenzhen Key Laboratory of Solid State Batteries, Institute of Major Scientific Facilities for New Materials, Southern University of Science and Technology, Shenzhen, Guangdong 518055, P. R. China.

*Corresponding email: xli@eitech.edu.cn; xsun@eitech.edu.cn; hansb@sustech.edu.cn; liangjianwen@grinm.com

Table of contents

Section 1. Materials and methods 1

Material fabrications .... 错误!未定义书签。

Materials characterizations 1

Electrochemical measurements 3

Mold-type ASSLBs assembly and electrochemical test 5

Pouch-type ASSLBs assembly and electrochemical measurements 5

LZOC-H Electrolyte Industrial Application Design by using current lithium-ion battery production methods 6

Section 2. Supplementary Figures 7

Section 3. Supplementary Tables 76

Section 4. Supplementary References 94

Section 1. Materials and methods

Materials characterizations

Phase analysis was performed using Powder X-ray diffraction (XRD) measurements on all materials at room temperature, utilizing a PANalytical Empyrean diffractometer with Cu-K_{α} radiation and a PIXcel bidimensional detector. XRD patterns were acquired using Bragg-Brentano geometry, with samples positioned on a zero-background holder within an Ar-filled glovebox and protected by Kapton film. Room-temperature neutron powder diffraction (NPD) measurements were carried out on the Multi-Physics Instrument at the China Spallation Neutron Source, employing a time-of-flight (TOF) configuration. A comprehensive Rietveld co-refinement of both the XRD and multi-detector TOF-NPD datasets was conducted using the GSAS-II suite[1-3]. Key structural parameters of the LZOC-H phase—including lattice constants, phase fractions, atomic coordinates, occupancy factors, and isotropic atomic displacement parameters (U_iso)—were refined simultaneously across both datasets. Given the negative coherent scattering length of Li for neutrons, Li site identification was aided by Fourier difference mapping. Final structure visualizations were prepared using the VESTA software[4].

A Hitachi S-4800 scanning electron microscope (SEM) coupled with an energy-dispersive X-ray (EDX) spectrometer was used to check the morphology and elemental distribution of samples. The customized inert transfer tank was used to transfer all of the samples while ensuring that they were protected appropriately. Cryogenic transmission electron microscopy (cryo-TEM) analysis was performed using a JEOL JEM-F200 microscope operated at 200 kV under cryogenic conditions (–180 ℃). For sample

preparation, the electrolyte powder was homogeneously deposited onto lacey carbon-supported grids within an Ar-filled glovebox (O_2<0.01 ppm, H_2O<0.01 ppm) to minimize air/moisture exposure. The prepared grids were subsequently loaded into a cryo-TEM holder under inert conditions to ensure preservation of the sample's native state during transfer and imaging.

X-ray photoelectron spectroscopic (XPS) characterization was carried out using a Krotos AXIS Ultra Spectrometer, which was equipped with monochromatic Al Kα radiation throughout the process. It was determined that the C1s location of the contaminated carbon, which was 284.8 eV, served as the reference for the calibration of the peak positions. The customized inert transfer tank was used to transfer all of the samples while ensuring that they were protected appropriately. The Thermo Fisher Scientific Raman spectrometer (DXR3xi, America) was utilized to capture the Raman spectra of the electrolytes under a laser beam at 532 nm. To prevent air exposure, the customized inert transfer tank was used to transfer all of the samples while ensuring that they were protected appropriately.

Solid-state ^{7}Li NMR characterization of LZOC-H solid electrolytes was performed using a Bruker Avance Neo 600WB spectrometer operating at a static magnetic field strength of 14.1 T (corresponding to 233.18 MHz for ^{7}Li nuclei). The system was equipped with a MASDVT600W2 BL3.2X/Y/H probe. All measurements employed zirconia rotors (3.2 mm outer diameter) under MAS conditions, with the rotor frequency carefully maintained at 15 kHz using a high-stability MAS controller to effectively suppress dipolar line broadening effects. Chemical shift referencing was implemented using LiCl as the external reference standard (δ = 0 ppm). Prior to NMR analysis, the LZOC-H specimens were mechanically

pulverized using an agate mortar to achieve particle sizes < 20 μm (preferably), ensuring optimal magnetic susceptibility distribution. The powdered samples were then meticulously packed into zirconia rotors in an Ar-filled glove box (O_2<0.01 ppm, H_2 O<0.01 ppm) and hermetically sealed with Kel-F end caps to prevent moisture absorption during data acquisition. All experiments were performed at room temperature.

The hard X-ray absorption spectroscopy (XAS) measurements were performed at beamline BL14W1 of the Hard X-ray Microfocus Platform at Shanghai Synchrotron Radiation Facility (SSRF). The Zr *K*-edge X-ray absorption near-edge structure (XANES) and extended X-ray absorption fine structure (EXAFS) spectra were collected using bulk-sensitive partial fluorescence yield (PFY) mode in an ultrahigh vacuum chamber with a base pressure better than 5×10^{-10} Torr at 300 K. All zirconium-containing samples were encapsulated with 6 μm Kapton film sandwiched between 100 nm Al foil barriers using an Ar-filled glove box (O_2<0.01 ppm, H_2O<0.01 ppm) before vacuum transfer to minimize oxidation artifacts. XANES data preprocessing, including energy calibration and atomic background removal, was conducted using the Demeter software package. The EXAFS data fitting with k^2-weighted Fourier transforms of the Zr *K*-edge spectra was performed via the Artemis module, incorporating *ab* initio FEFF10 calculations. Wavelet transform (WT) analysis of k^2-weighted EXAFS oscillations was implemented using the Waveme toolkit for three-dimensional scattering path visualization.

Electrochemical measurements

Electrochemical impedance spectroscopy (EIS) was employed to determine the ionic conductivity of the LZOC samples. In a typical experiment, 150 mg of LZOC-H, LZOC-A, or

LZOC-O powder was loaded between two stainless-steel rods and pressed into 10 mm diameter pellets using a hydraulic press at 300 MPa for 3 min in a mold cell in an Ar-filled glove box. EIS measurements were conducted at an AC amplitude of 10 mV with a VMP3 potentiostat (Bio-Logic) over a frequency range of 7 MHz to 1 Hz while maintaining an applied pressure of 300 MPa. For activation energy (E_a) measurements, 150 mg of the LZOC-H, LZOC-A, or LZOC-O powder was similarly pressed into 10 mm diameter pellets using a mold cell under 300 MPa for 3 min. Impedance measurements were then performed over a temperature range from –65 to 65 ℃, with the frequency swept from 7 MHz to 1 Hz. Electronic conductivity was determined via the DC polarization method. For this measurement, 150 mg of the same powder was pressed into a 10 mm diameter pellet under 300 MPa for 3 min in a mold cell in the argon-filled glove box. The pellet was then secured in a stainless-steel cage to ensure a constant pressure of approximately 300 MPa, and the current responses of the cell were measured at a range of constant voltages for 60 min each. The applied voltage ranged from 0.2 to 0.6 V with a step size of 0.1 V.

For linear sweep voltammetry (LSV) measurements, 80 mg of LPSC was placed into a mold cell (with 10 mm diameter stainless-steel rods) and pressed at 150 MPa for 3 min to form a pellet. Next, 30 mg of LZOC-H powder was evenly spread on one side of the LPSC pellet and pressed at 300 MPa for an additional 3 min. To prepare the LZOC-H–carbon nanofiber composite, LZOC-H, and vapor-grown carbon fiber (VGCF) composite were mixed in a 50:50 weight ratio and hand-ground in an agate mortar for 15 min. Approximately 10 mg of the composite was then placed on the same side of the solid electrolyte pellet to serve as the working electrode and pressed at 300 MPa for another 3 min. The counter

electrode assembly comprised a bilayer structure, a 10 mm diameter indium (In) foil (200 μm thickness) current collector overlain by ~4 mg of lithium metal roll-pressed, ensuring uniform Li^+ flux distribution. The cell was subsequently assembled into a stainless-steel casing and maintained at a constant pressure of 150 MPa. LSV measurements were performed at a scan rate of 0.1 mV s^{-1} using a VMP3 potentiostat (Bio-Logic).

Mold-type ASSLBs assembly and electrochemical test

For cathode composite preparation, commercial–grade $LiNi_{0.89}Co_{0.06}Mn_{0.05}O_2$ (Ni89, China Automotive Battery Research Institute Co, Ltd), LZOC-H/LZOC-A/LZOC-O, and Super P were weighted in a 70:30:1 weight ratio and mixed using an agate mortar for 30 minutes. For cell fabrication, 50 mg of Zr-based HSSE powder was placed into a 10 mm diameter stainless steel mold and then uniaxially pressed at 50 MPa; then 50 mg LPSC powder was placed into this mold and then uniaxially pressed at 50 MPa. Subsequently, 10 mg of the cathode composite was uniformly spread on one side of the Zr-based HSSE pellet and uniaxially pressed at 300 MPa. On the other side of the SSSB pellet, a Li-In alloy was placed and pressed at approximately 100 MPa. Finally, an external pressure of ~100 MPa was applied for the cell performance tests on the Land battery testing systems.

Pouch-type ASSLBs assembly and electrochemical measurements

Pouch-type ASSLBs were fabricated using a dry processing method. First, the composite positive electrode was fiberized with a mass ratio of Ni89: LZOC-H: VGCF: PTFE = 70:30:1:1. The resulting material was then processed into a ~200 μm-thick film via heated roller pressing at 90 °C. This film was cut into 3.5 × 5.0 cm electrode pieces and laminated onto aluminum foil current collectors by roller pressing. A 150 μm-thick cathode-side solid

electrolyte film was prepared by roller pressing a fibrillated mixture of LZOC-H and PTFE (99:1 by mass). Likewise, a 150 μm-thick anode-side electrolyte film was fabricated using LPSC and PTFE in the same 99:1 mass ratio. Both electrolyte films were cut into 4.5 × 6.0 cm sheets. For the anode, a 100 μm-thick indium foil or a silicon-based electrode (4 mAh cm^{-2}) was employed, paired with a 4.0 × 5.5 cm copper foil current collector. The components were assembled into a single-layer all-solid-state pouch cell, which was vacuum-sealed using aluminum–plastic laminate film. Detailed specifications for pouch cells are listed in **Table S16**. Following isostatic pressing, the assembled battery was subjected to electrochemical testing under a stacking pressure of 150 MPa using a Neware battery testing system.

LZOC-H Electrolyte Industrial Application Design by using current lithium-ion battery production methods

Step 1: Using commercial lithium-ion battery dry-room conditions, a dry-film fabrication strategy is employed in which the active materials, solid-state electrolytes, conductive additives, and binders are dry-mixed and subsequently processed into self-supporting films by hot rolling or pressing. The detailed procedure is as follows.

Step 2: The cathode film, composed of Ni89, LZOC-H, Super P, and PTFE, is prepared with a mass ratio of 80:20:1:1. The electrolyte films, namely LZOC-H + PTFE and LPSC + PTFE, are each formulated at a mass ratio of 99:1, where achieving effective PTFE fibrillization is essential for film integrity and mechanical robustness.

Step 3: The silicon–carbon anode is fabricated using a conventional slurry-based coating process.

Step 4: Cell assembly is conducted by sequential stacking in the following order: cathode current collector (Al foil), cathode film, LZOC electrolyte film, LPSC electrolyte film, anode film, and anode current collector (Cu foil).

Step 5: The stacked cell is then pre-sealed and subjected to isostatic pressing, either under cold or hot pressing conditions, to ensure intimate interfacial contact. Finally, the fully sealed pouch cell undergoes initial charge–discharge activation and subsequent electrochemical performance evaluation.

Section 2. Supplementary Figures

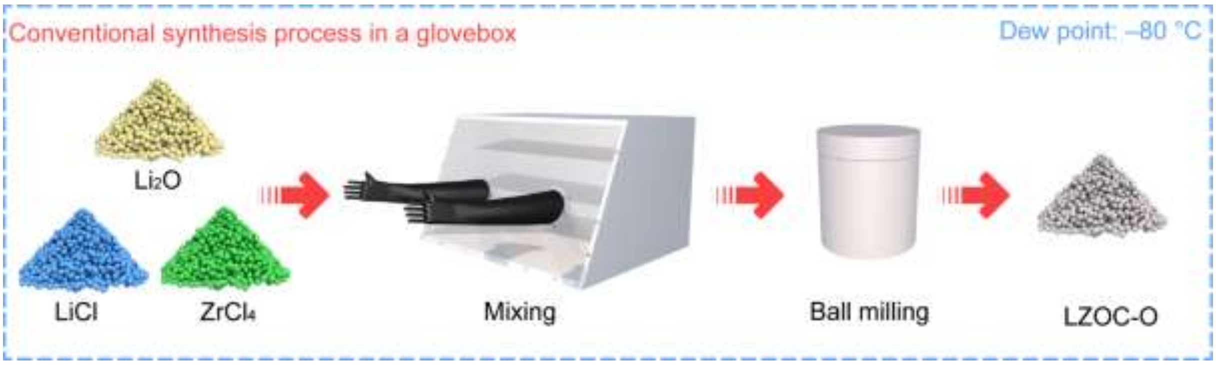

Fig. S1 Illustrate the schematic diagram of the material synthesis technology route for the conventional synthesis process of the Zr-based HSSEs.

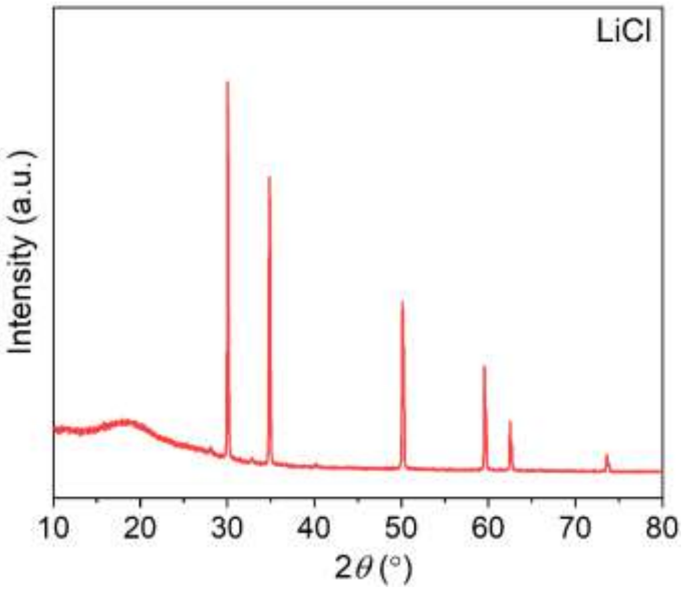

Fig. S2 XRD pattern of LiCl.

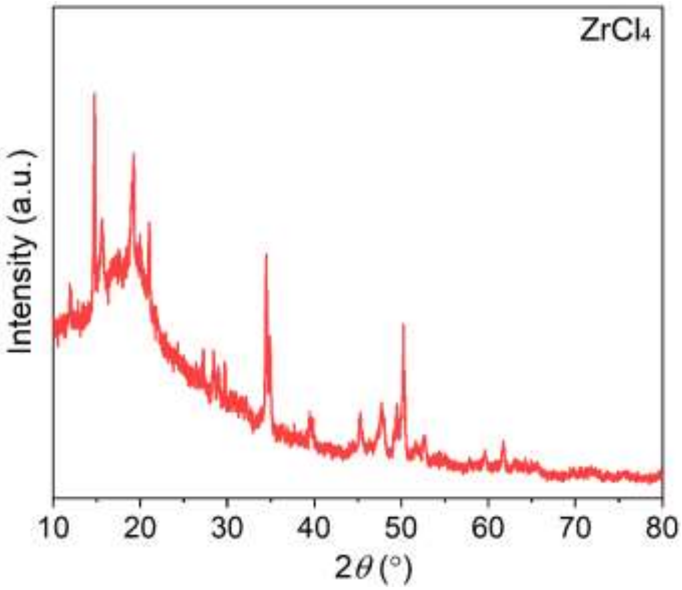

Fig. S3 XRD pattern of $ZrCl_4$.

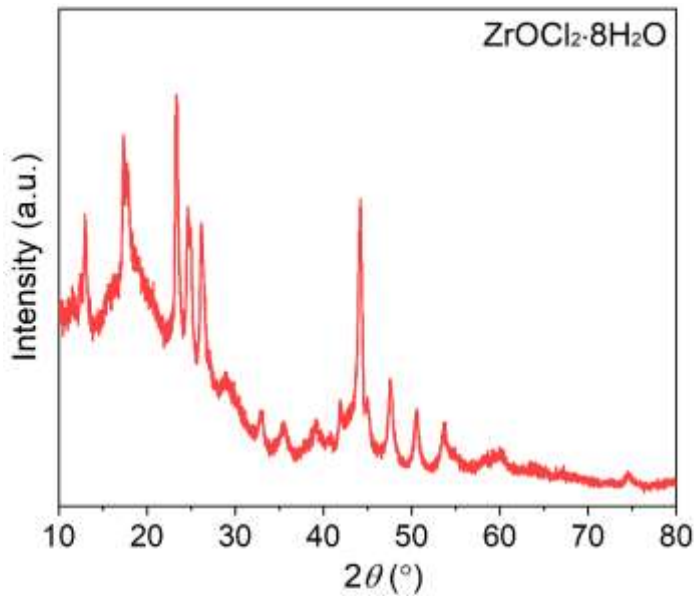

Fig. S4 XRD pattern of $ZrOCl_2 \cdot 8H_2O$.

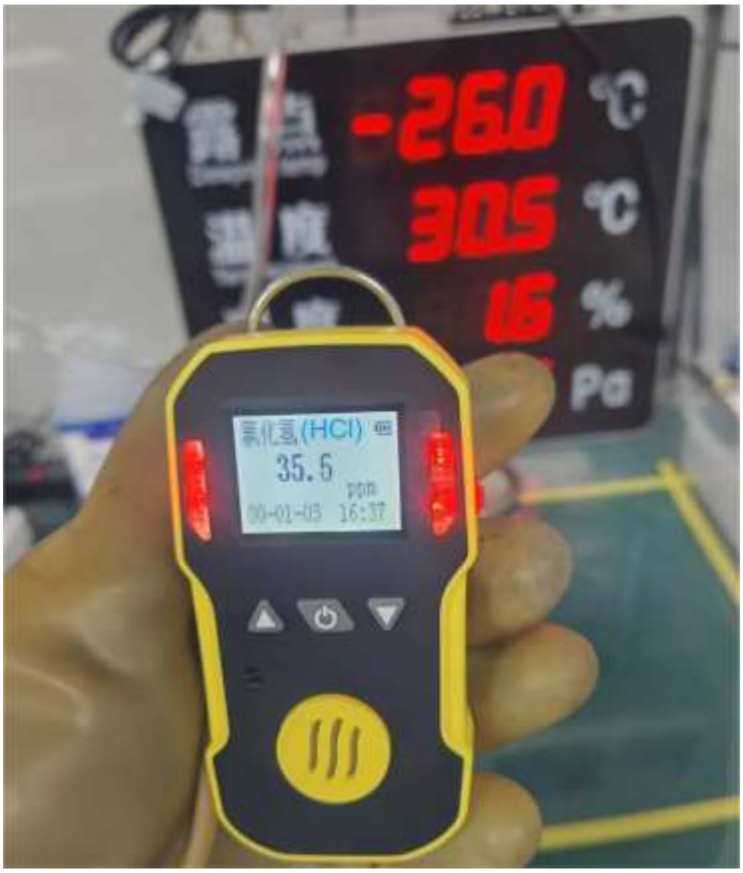

Fig. S5 HCl gas release during the ball milling process of LZOC-H.

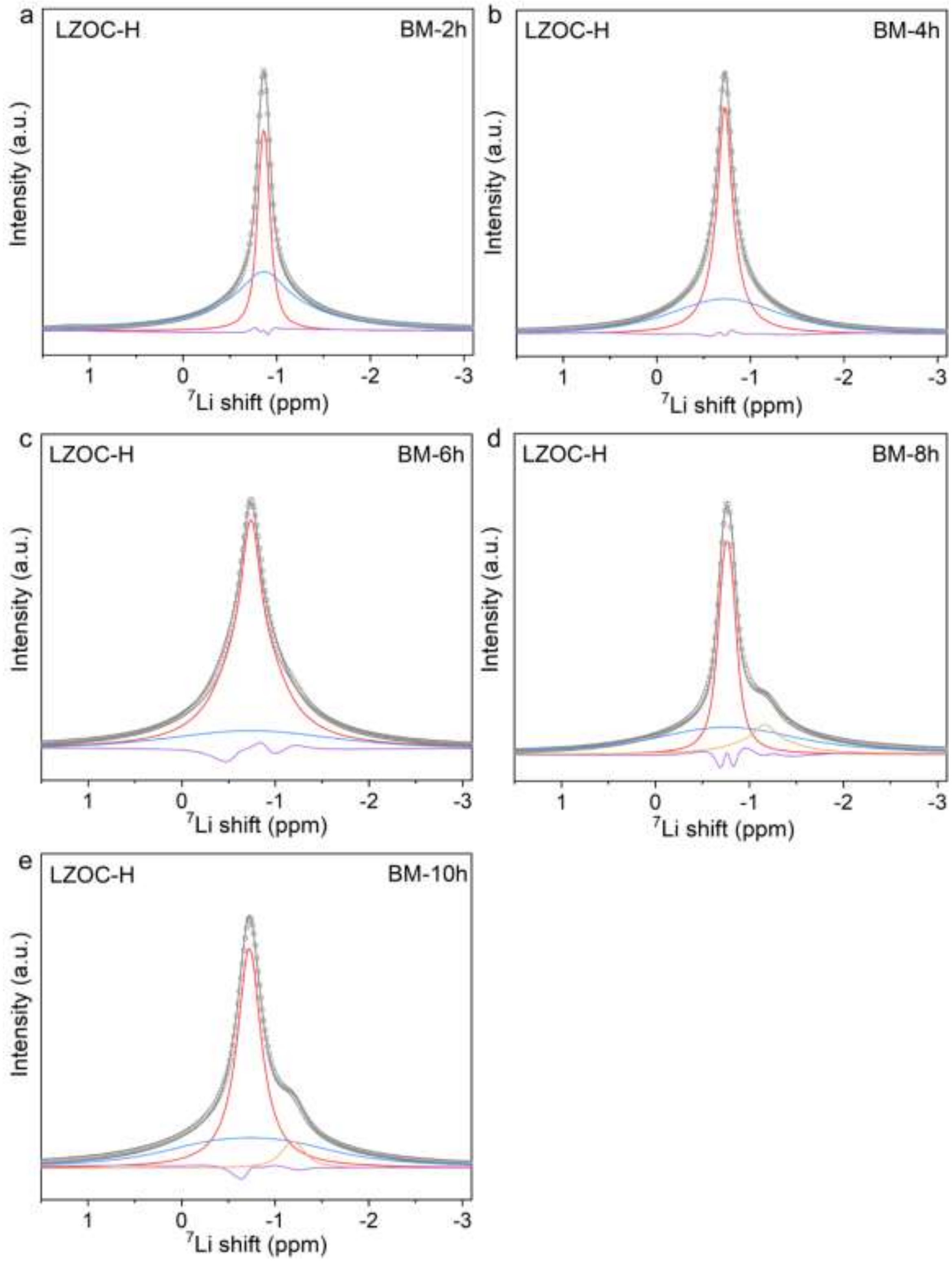

Fig. S6 Fitting results for ^{7}Li MAS NMR spectra of LZOC-H HSSE with different ball-milling times (a) 2 h, (b) 4 h, (c) 6 h, (d), 8 h and (e) 10 h.

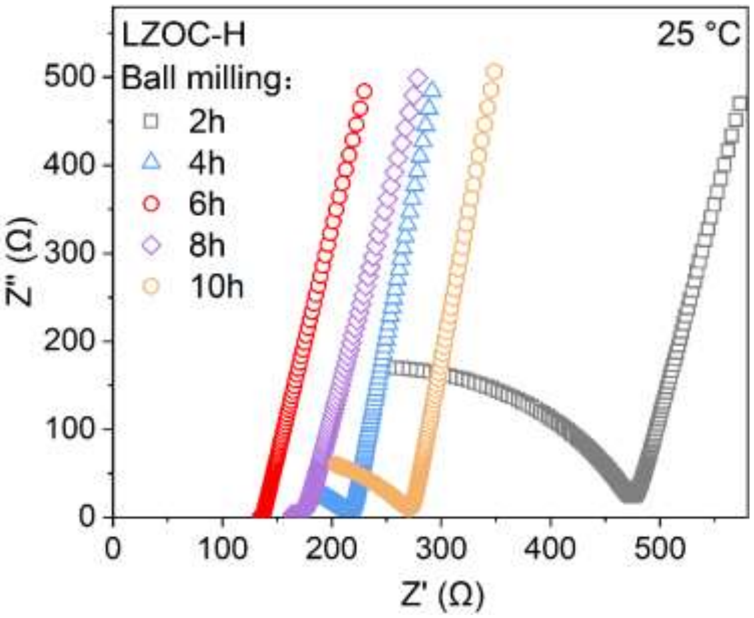

Fig. S7 Nyquist plots of LZOC-H HSSE (thickness of 1.2 mm) at different ball-milling times at 25 ℃.

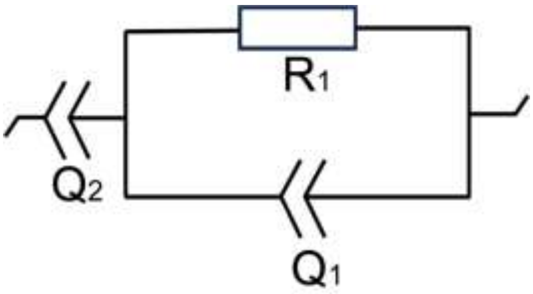

Fig. S8 The equivalent circuit model for the fitting of the Nyquist plot of LZOC samples; R_1 is bulk electrolyte resistance, Q_1 is constant-phase elements, and Q_2 is Warburg impedance.

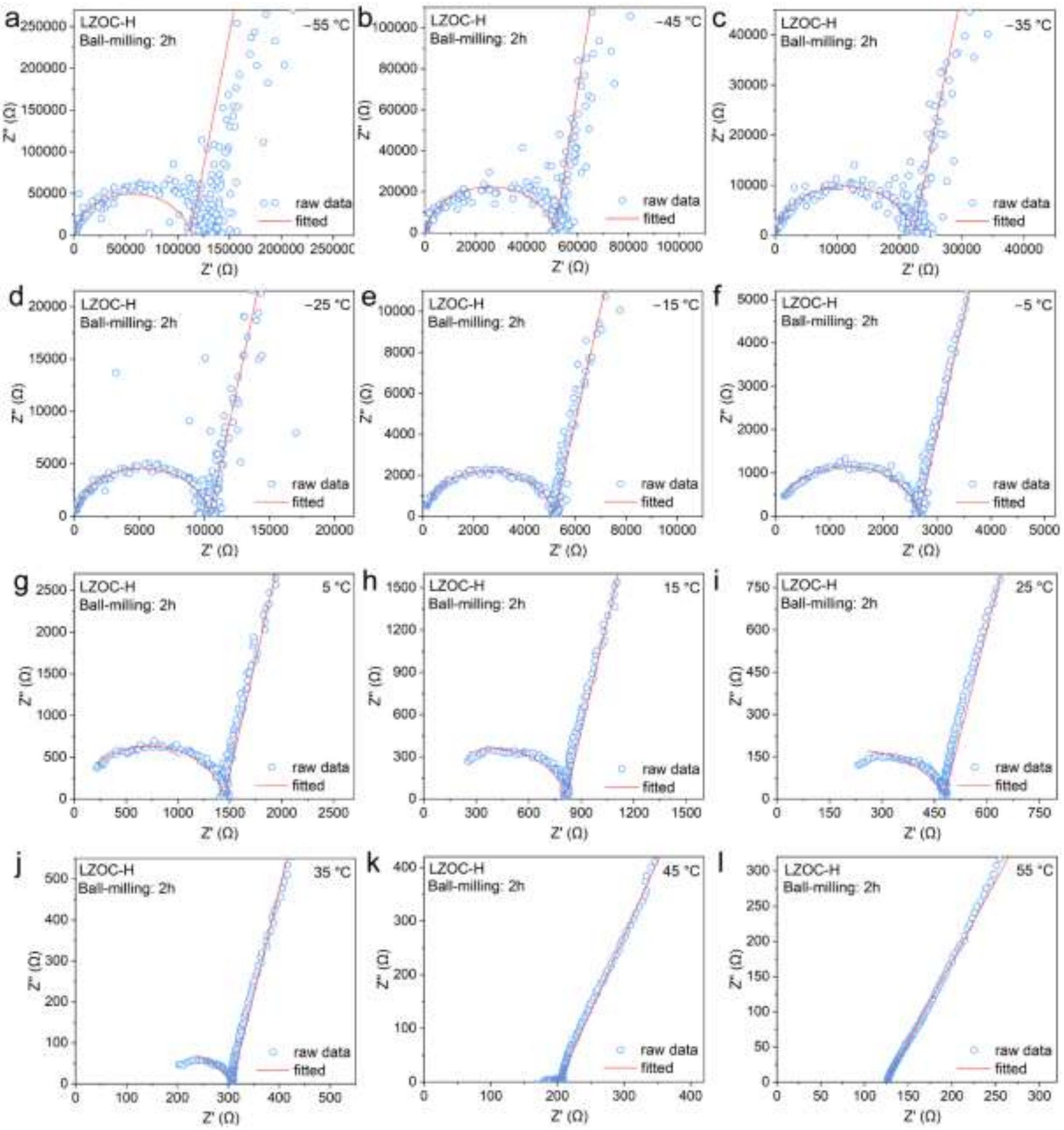

Fig. S9 Nyquist plots of LZOC-H HSSE measured immediately after ball-milling time of 2 h at various temperatures: (a) –55 ℃, (b) –45 ℃, (c) –35 ℃, (d) –25 ℃, (e) –15 ℃, (f) –5 ℃, (g) 5 ℃, (h) 15 ℃, (i) 25 ℃, (j) 35 ℃, (k) 45 ℃, and (l) 55 ℃.

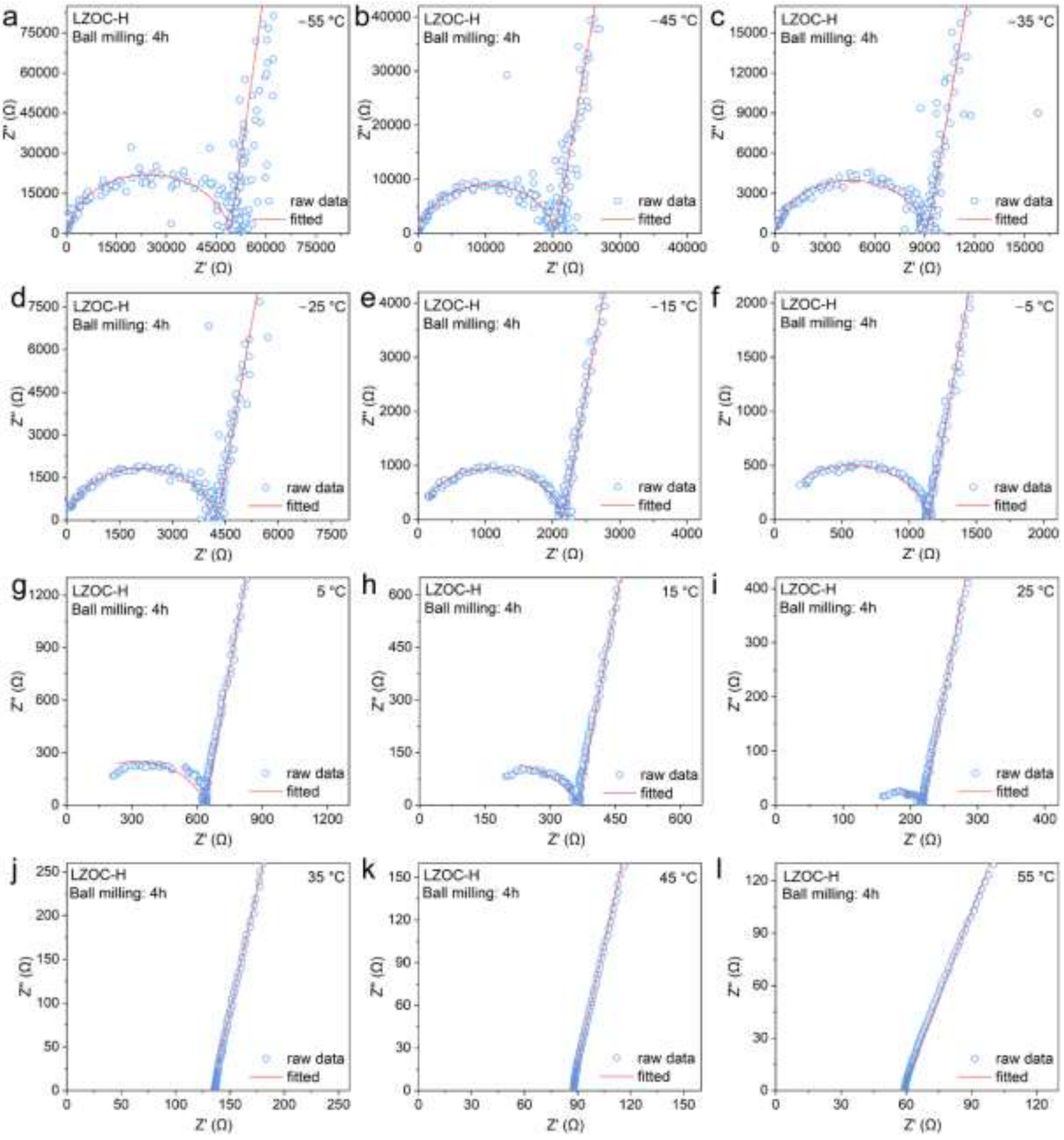

Fig. S10 Nyquist plots of LZOC-H HSSE measured immediately after ball-milling time of 4 h at various temperatures: (a) –55 ℃, (b) –45 ℃, (c) –35 ℃, (d) –25 ℃, (e) –15 ℃, (f) –5 ℃, (g) 5 ℃, (h) 15 ℃, (i) 25 ℃, (j) 35 ℃, (k) 45 ℃, and (l) 55 ℃.

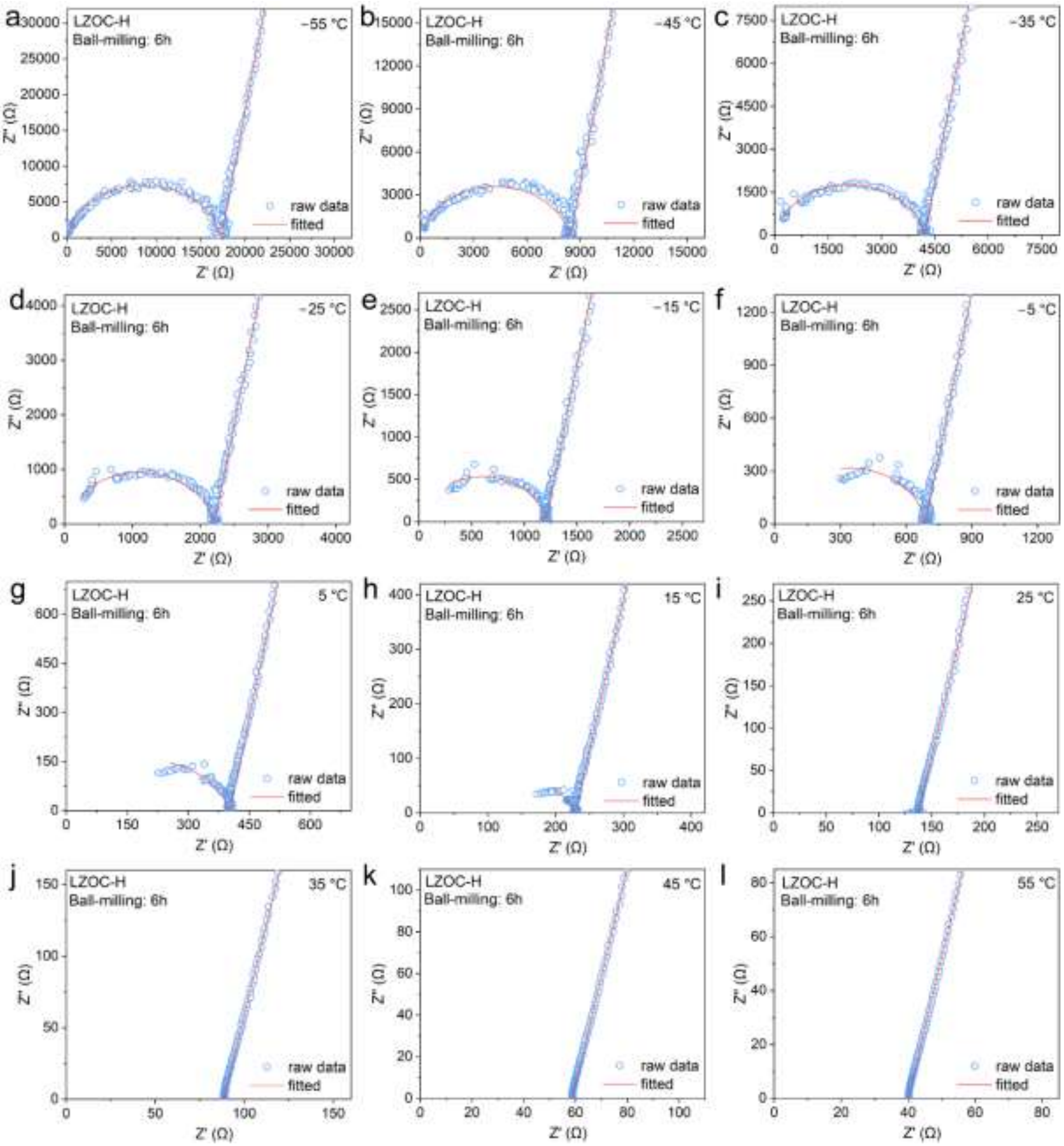

Fig. S11 Nyquist plots of LZOC-H HSSE measured immediately after ball-milling time of 6 h at various temperatures: (a) –55 ℃, (b) –45 ℃, (c) –35 ℃, (d) –25 ℃, (e) –15 ℃, (f) –5 ℃, (g) 5 ℃, (h) 15 ℃, (i) 25 ℃, (j) 35 ℃, (k) 45 ℃, and (l) 55 ℃.

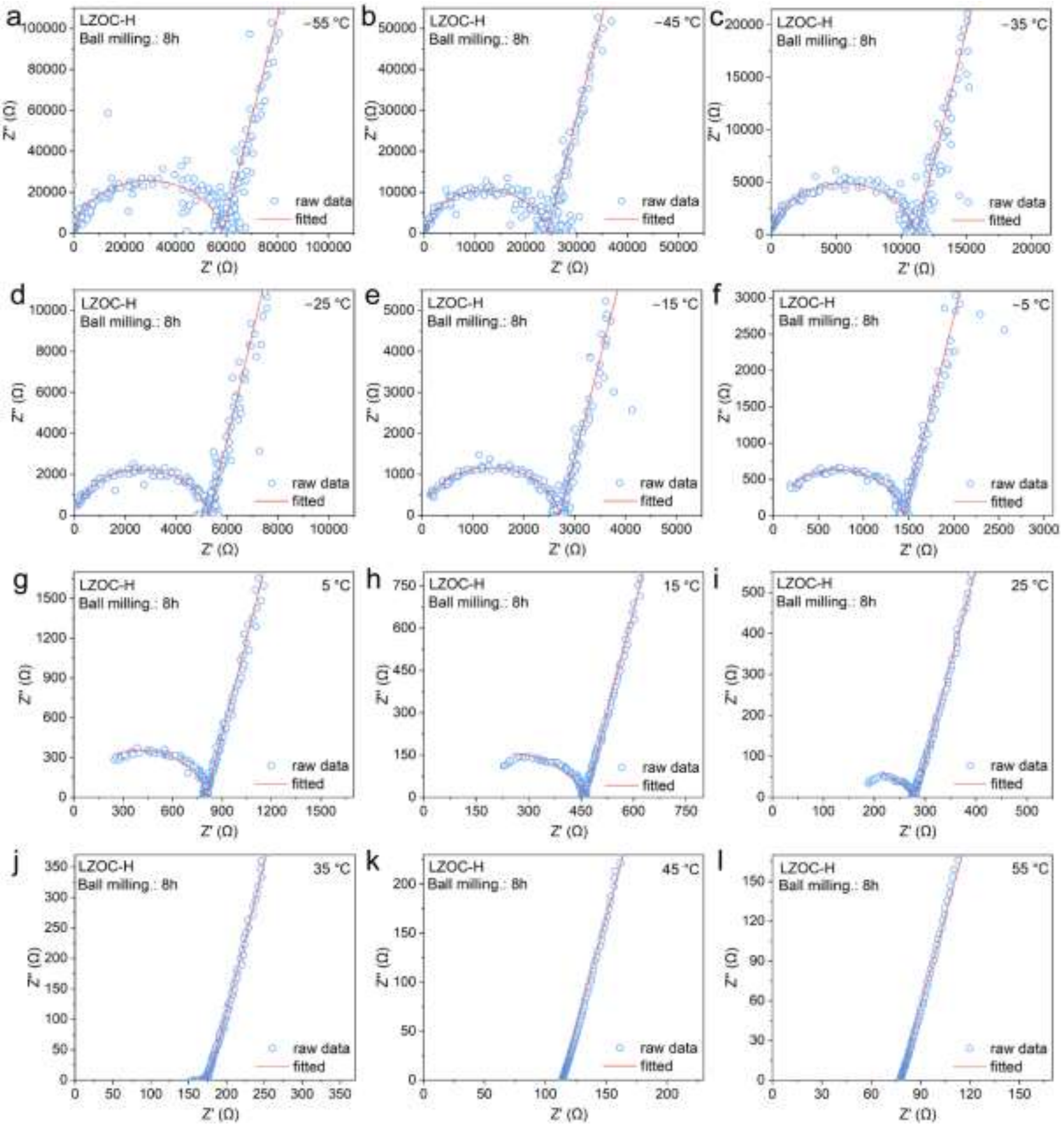

Fig. S12 Nyquist plots of LZOC-H HSSE measured immediately after ball-milling time of 8 h at various temperatures: (a) –55 ℃, (b) –45 ℃, (c) –35 ℃, (d) –25 ℃, (e) –15 ℃, (f) –5 ℃, (g) 5 ℃, (h) 15 ℃, (i) 25 ℃, (j) 35 ℃, (k) 45 ℃, and (l) 55 ℃.

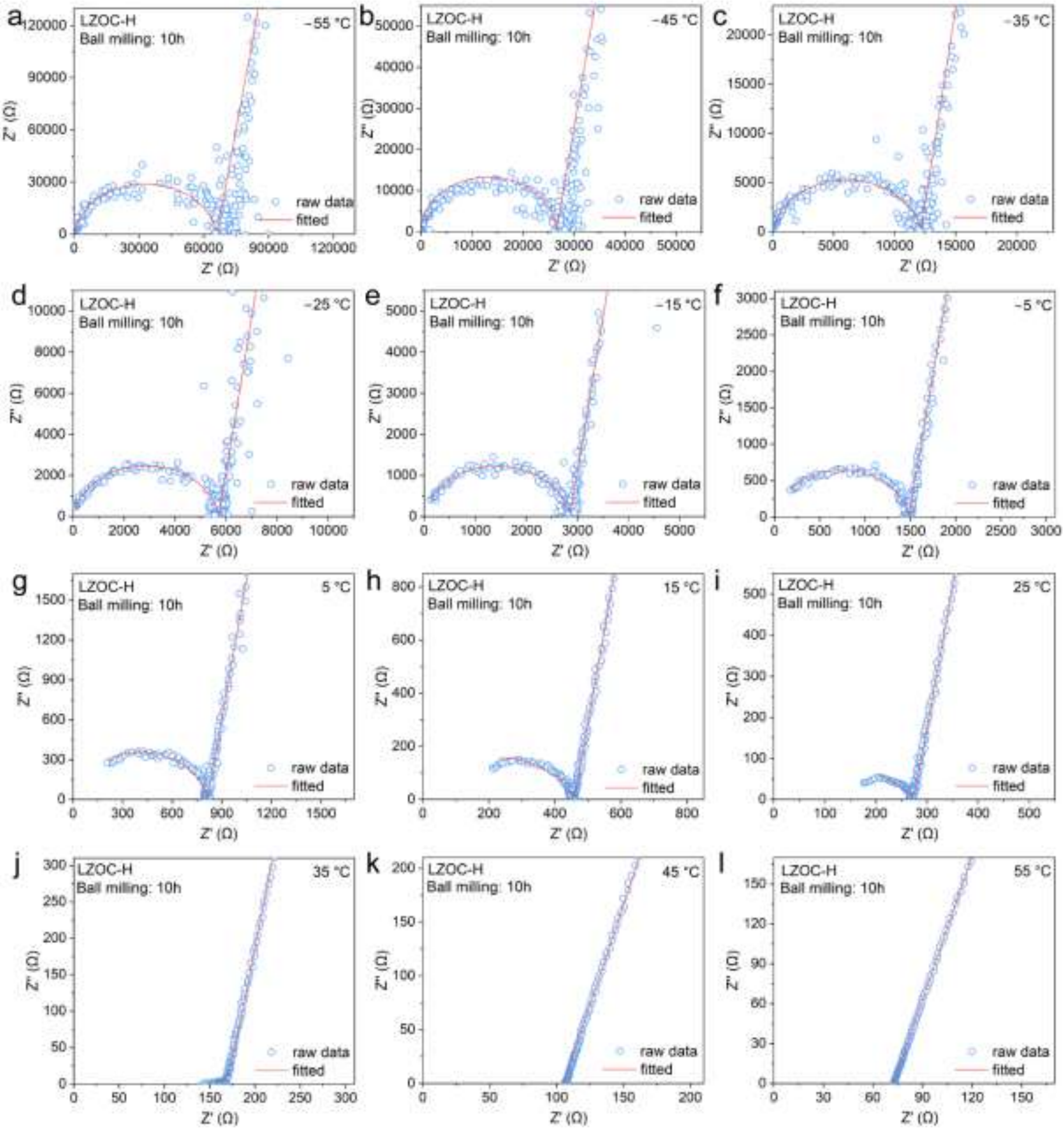

Fig. S13 Nyquist plots of LZOC-H HSSE measured immediately after ball-milling time of 10 h at various temperatures: (a) –55 ℃, (b) –45 ℃, (c) –35 ℃, (d) –25 ℃, (e) –15 ℃, (f) –5 ℃, (g) 5 ℃, (h) 15 ℃, (i) 25 ℃, (j) 35 ℃, (k) 45 ℃, and (l) 55 ℃.

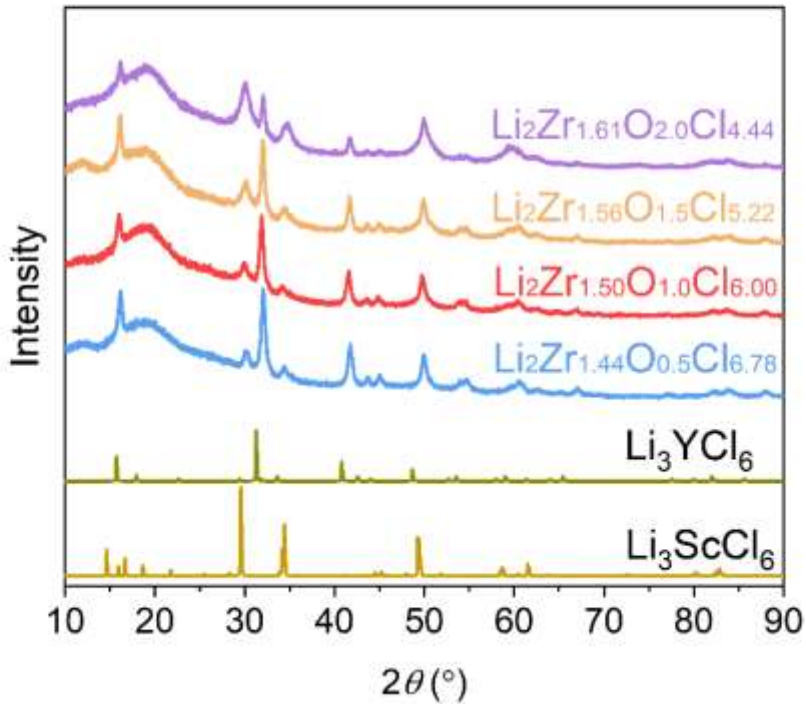

Fig. S14 XRD patterns of LZOC-H HSSE with different oxide element content.

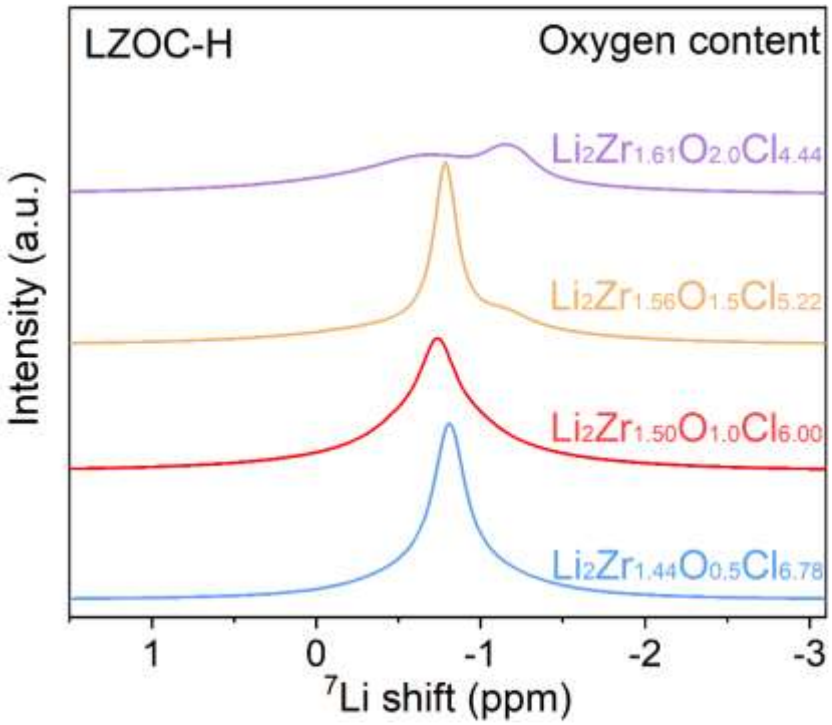

Fig. S15 NMR patterns of LZOC-H HSSE with different oxide element content.

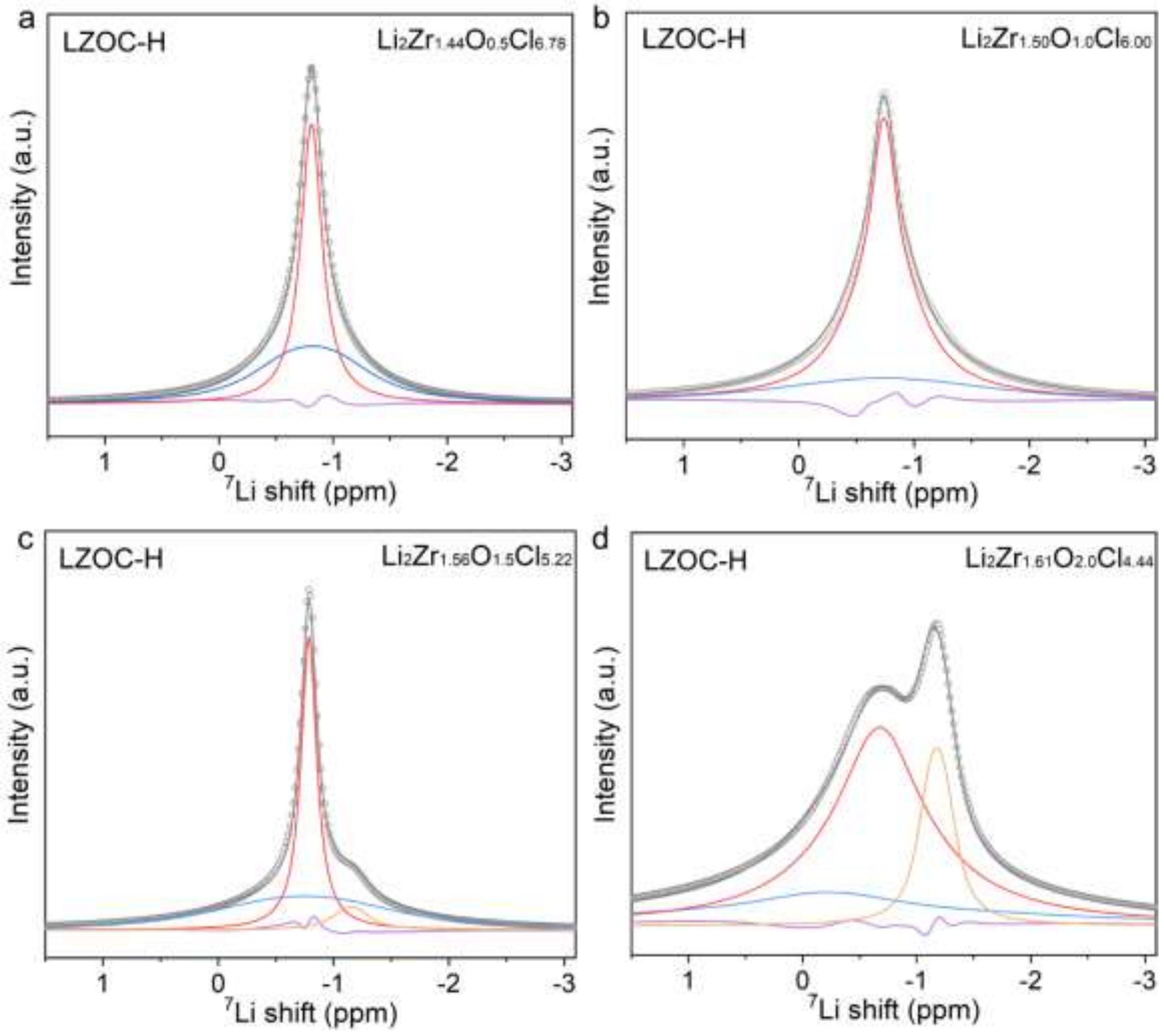

Fig. S16 Fitting results for ^{7}Li MAS NMR spectra of LZOC-H HSSEs with different oxide element contents (a) $Li_2Zr_{1.44}O_{0.5}Cl_{6.78}$, (b) $Li_2Zr_{1.50}O_{1.0}Cl_{6.00}$, (c) $Li_2Zr_{1.56}O_{1.5}Cl_{5.22}$, and (d) $Li_2Zr_{1.61}O_{2.0}Cl_{4.44}$.

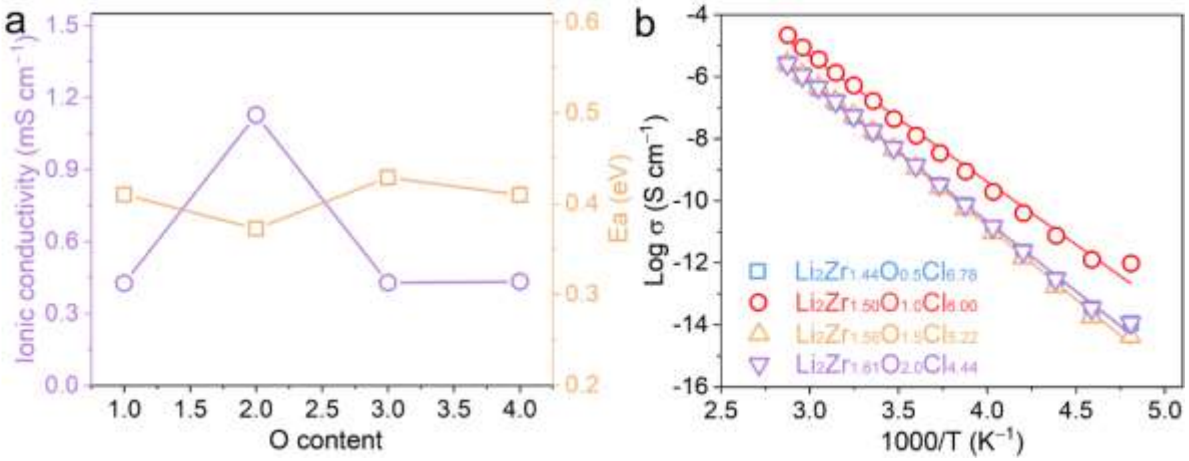

Fig. S17 Ionic conductivities and active energies of LZOC-H HSSEs with different oxide element content.

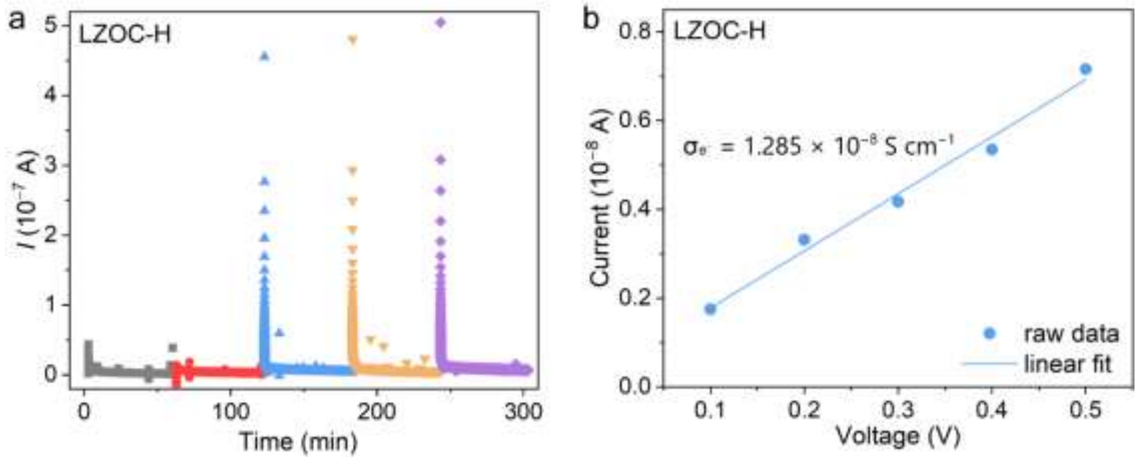

Fig. S18 (a) DC polarization curves and (b) the corresponding equilibrium current response of the LZOC-H HSSE (ball-milling 6 hours) using ion-blocking symmetric cell at different voltages from 0.1 to 0.5 V.

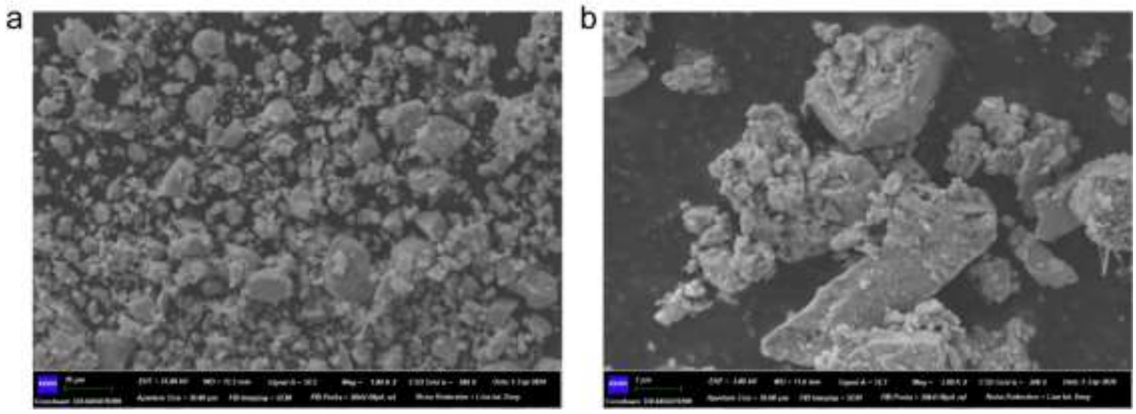

Fig. S19 SEM images of LZOC-H HSSE after ball-milling 6 hours.

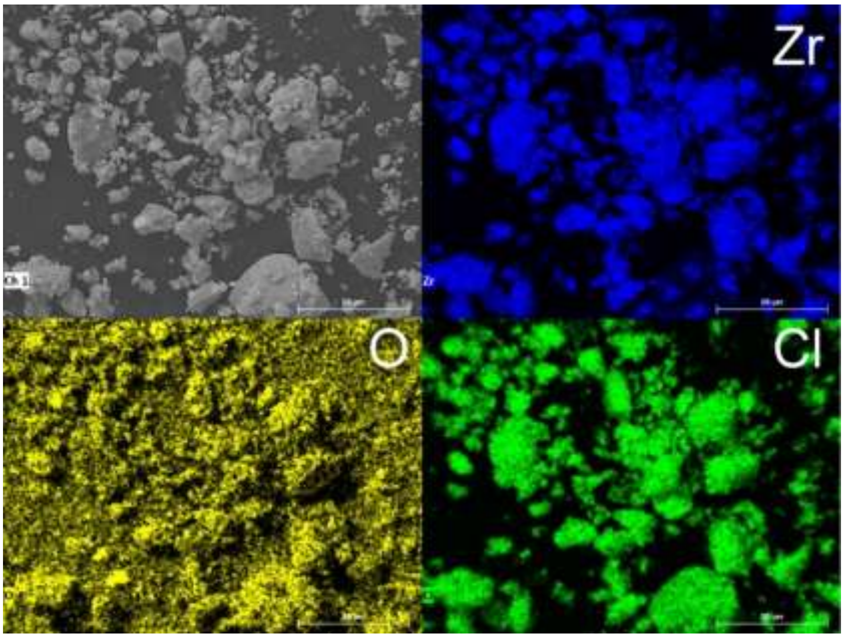

Fig. S20 SEM-EDS images of LZOC-H HSSE after ball-milling 6 hours.

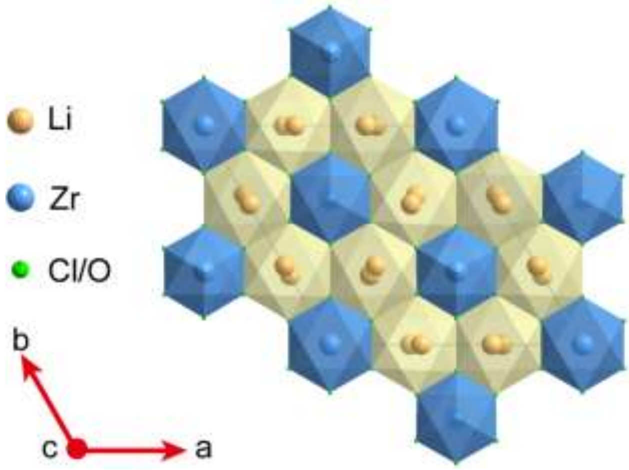

Fig. S21 Crystal structural diagram of LZOC-H HSSE.

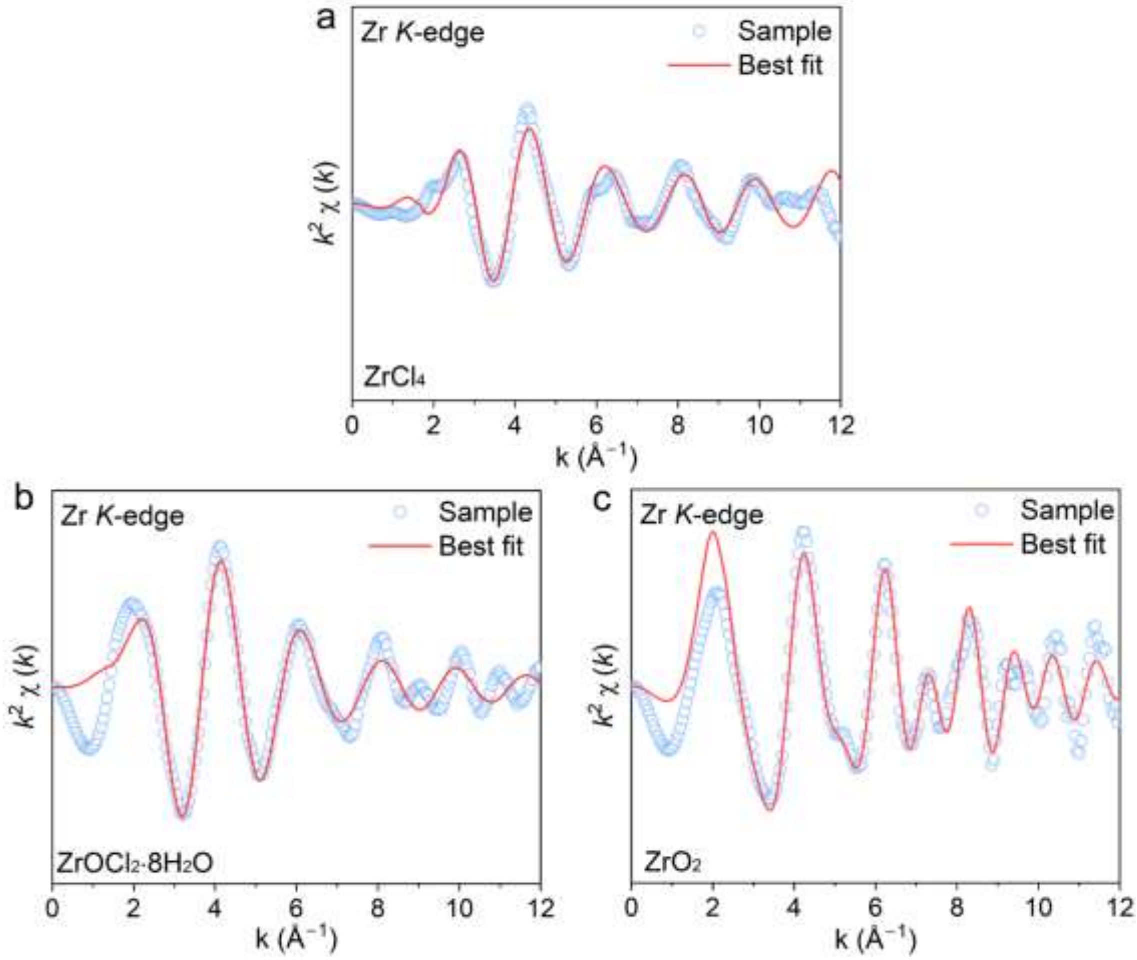

Fig. S22 Zr K-edge Fourier-transformed (FT) EXAFS fitting results of (a) $ZrCl_4$, (b) $ZrOCl_2 \cdot 8H_2O$, and (c) ZrO_2, showing the experimental data (blue circle) and Feff modeling (red line) traces.

.

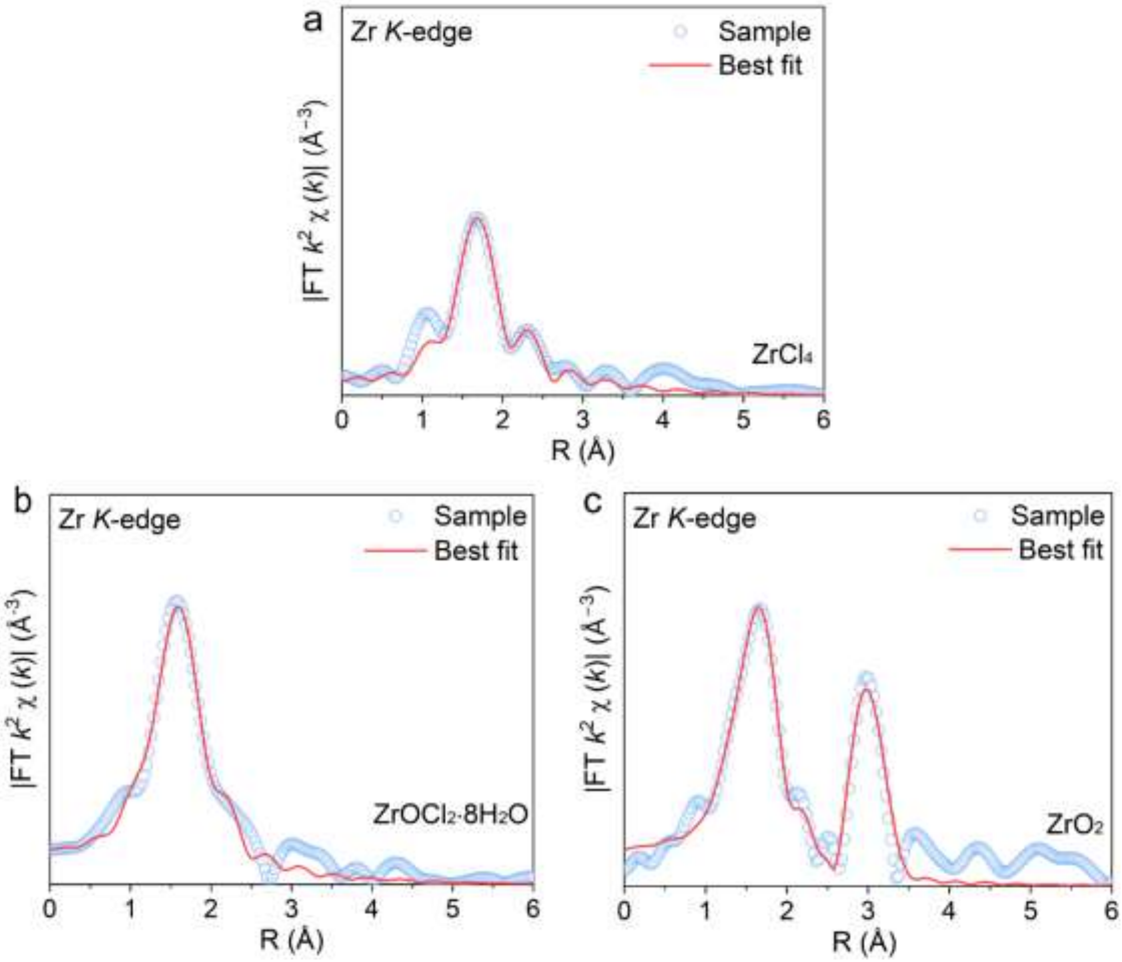

Fig. S23 Fitting results of the *k2*-weighted Fourier-transformed (FT) spectra of (a) $ZrCl_4$, (b) $ZrOCl_2 \cdot 8H_2O$, and (c) ZrO_2 at Zr K-edge, showing the experimental data (blue circle) and Feff modeling (red line) traces.

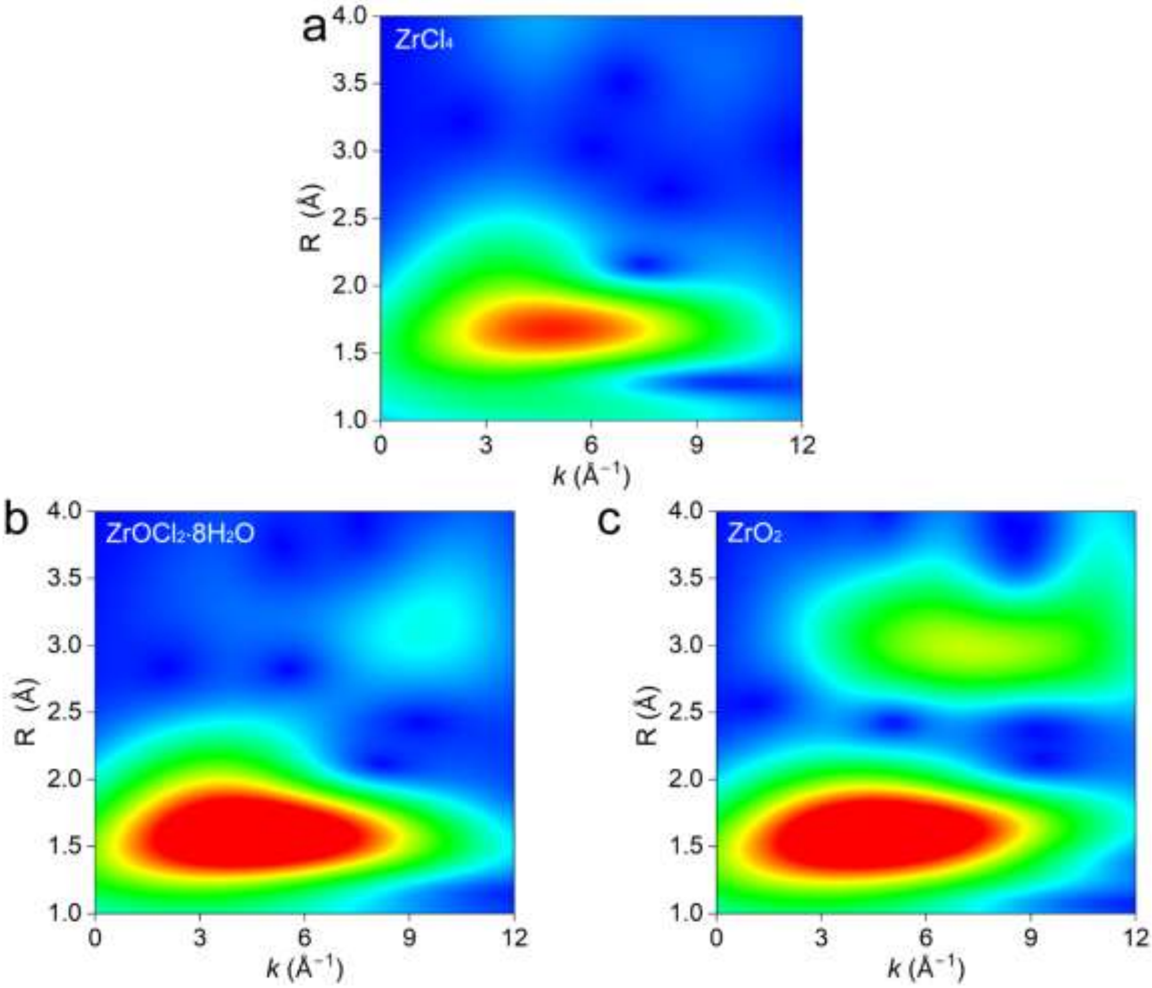

Fig. S24 Wavelet transformed (WT) Extended X-ray Absorption Fine Structure (EXAFS) contour plots of (a) $ZrCl_4$, (b) $ZrOCl_2 \cdot 8H_2O$, and (c) ZrO_2 at the Zr K-edge with a k^2 weighting.

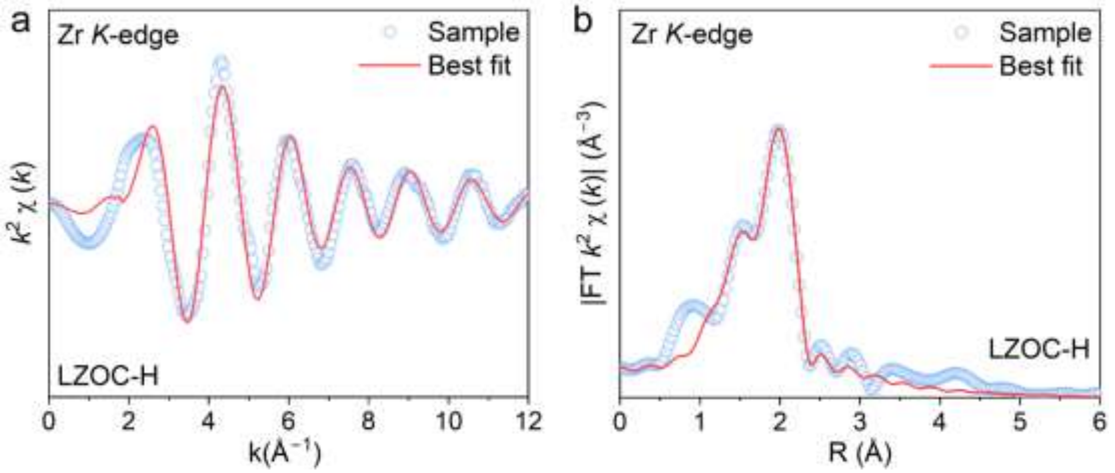

Fig. S25 Zr K-edge Fourier-transformed (FT) EXAFS fitting results of LZOC-H HSSE, showing the experimental data (blue circle) and Feff modeling (red line) traces.

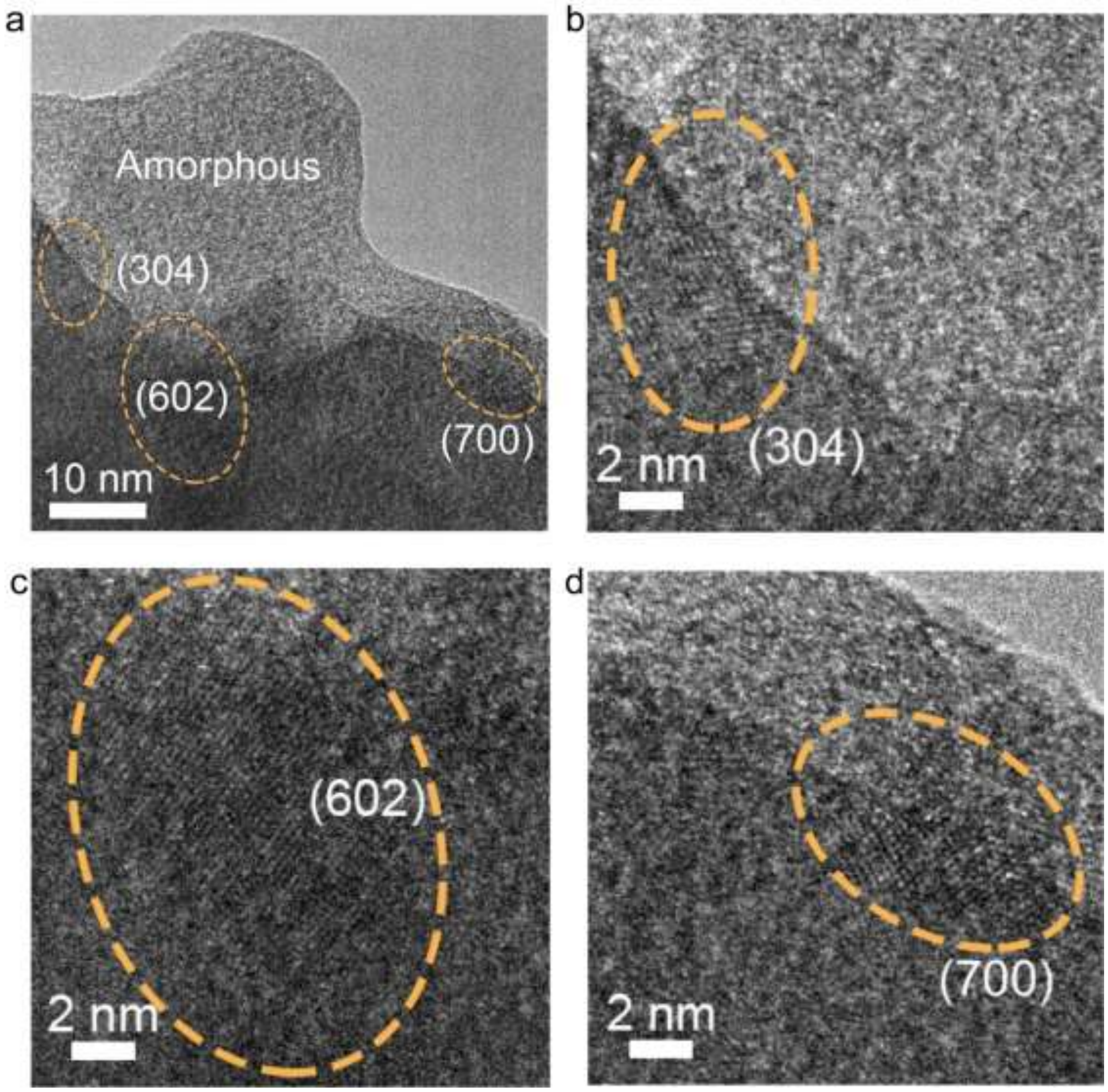

Fig. S26 The HRTEM images of LZOC-H HSSE.

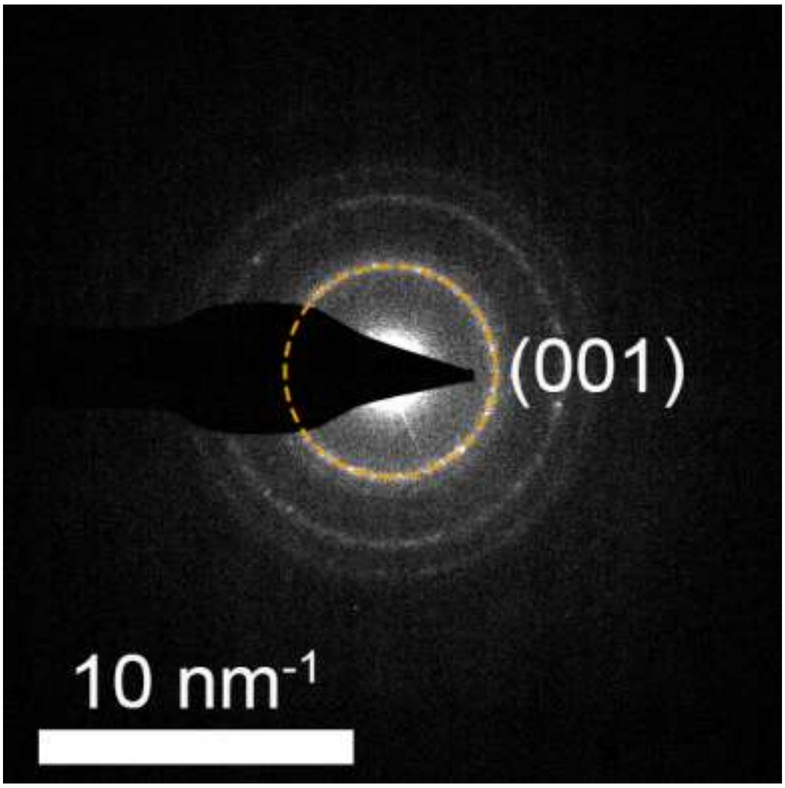

Fig. S27 The SAED image of LZOC-H HSSE.

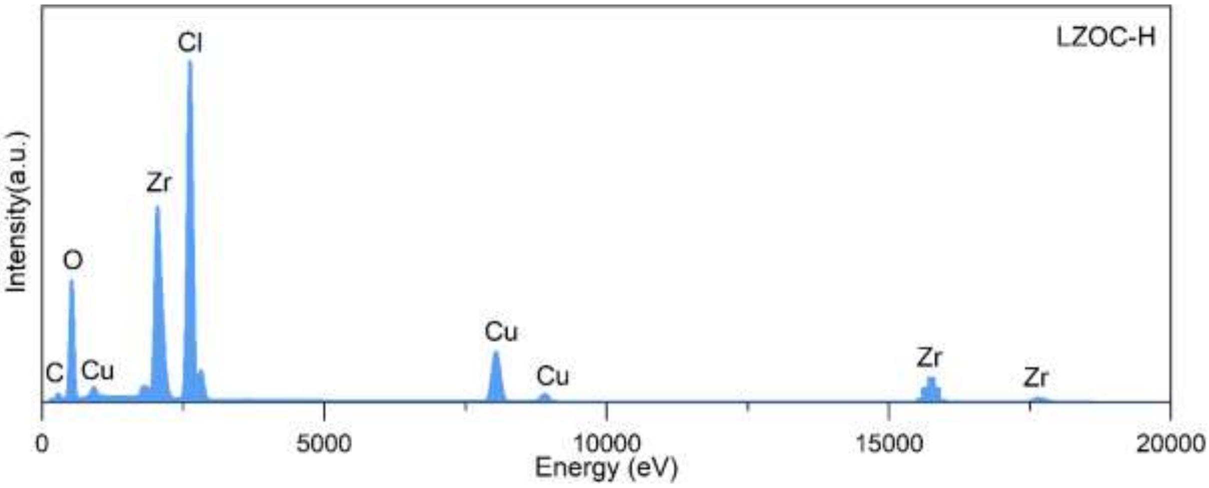

Fig. S28 Spectra from Area of TEM-EDS for LZOC-H HSSE.

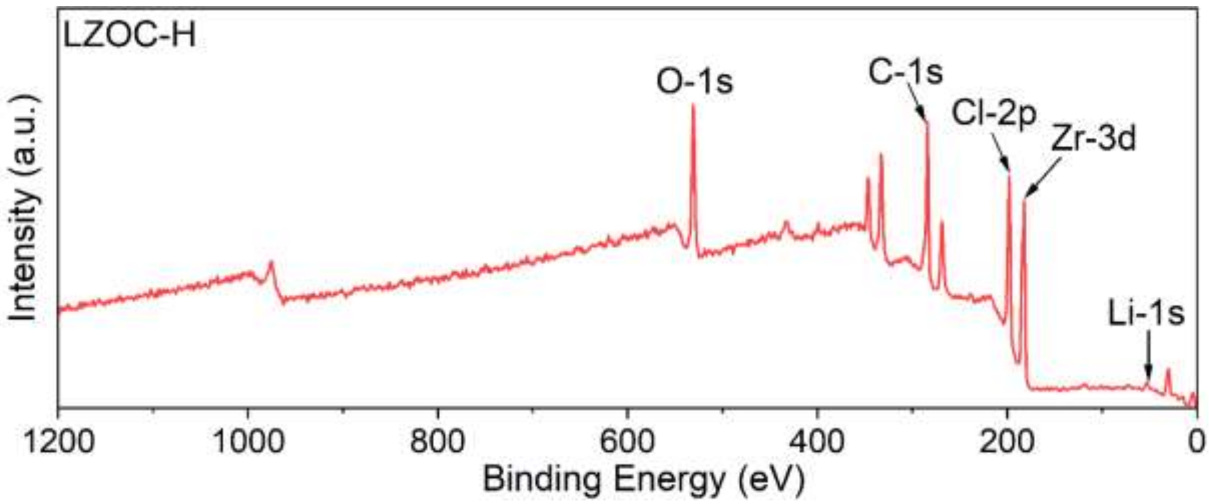

Fig. S29 XPS survey spectra of LZOC-H HSSE.

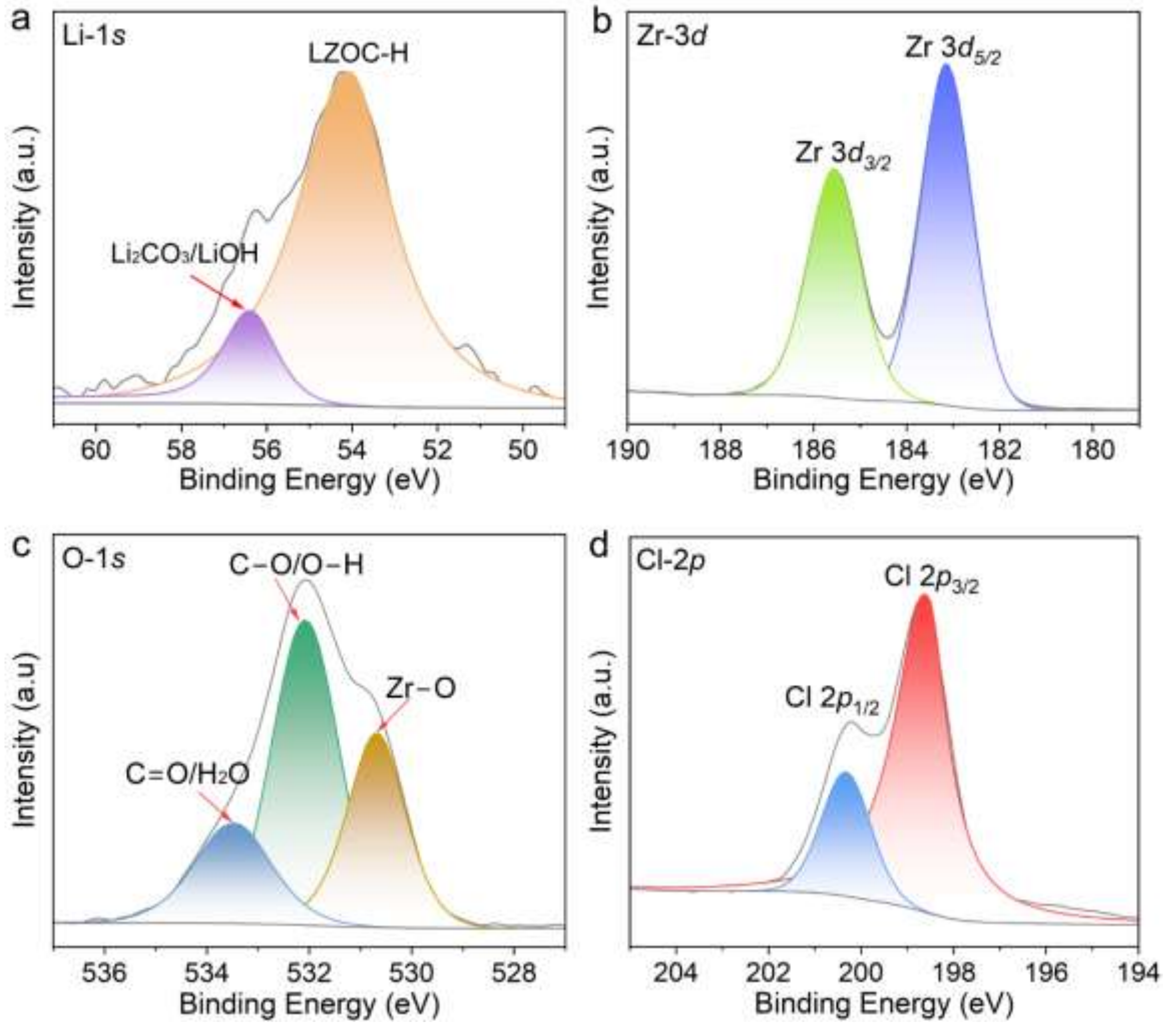

Fig. S30 XPS spectra of (a) Li-1*s*, (b) Zr-3*d*, (c) O-1*s*, (d) Cl-2*p* in LZOC-H HSSE.

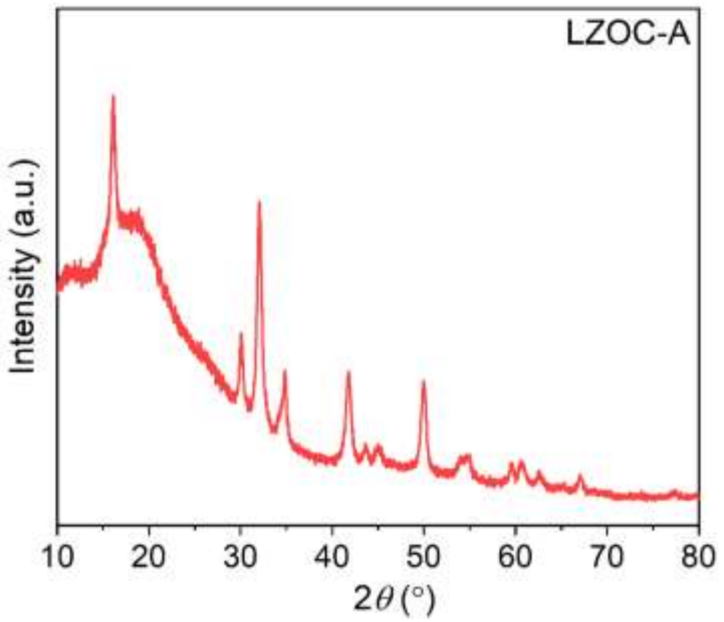

Fig. S31 XRD Pattern of the LZOC-A HSSE.

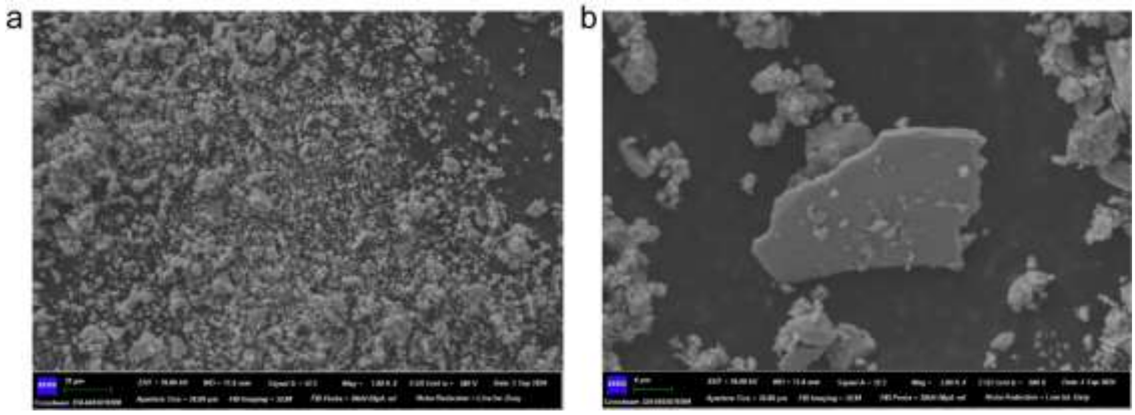

Fig. S32 SEM images of the LZOC-A HSSE.

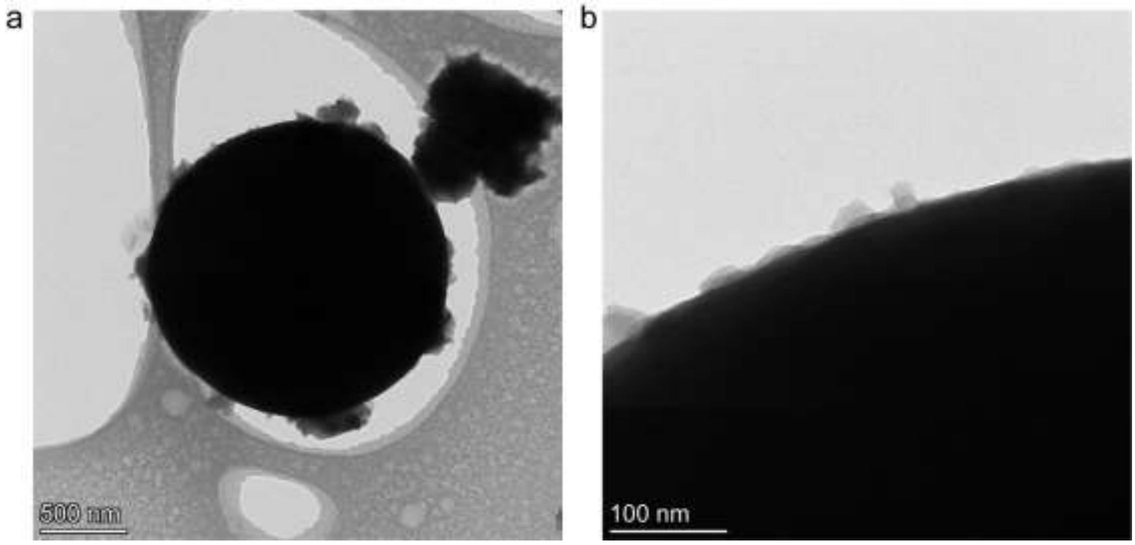

Fig. S33 TEM images of the LZOC-A HSSE.

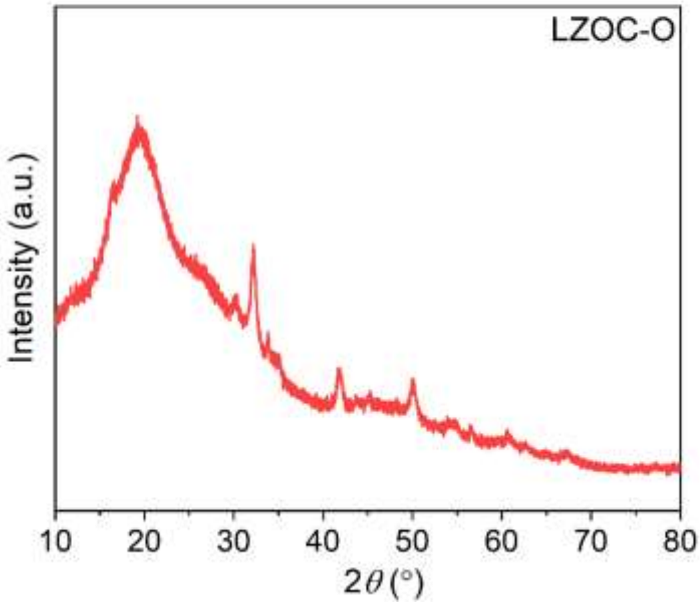

Fig. S34 XRD Pattern of the LZOC-O HSSE.

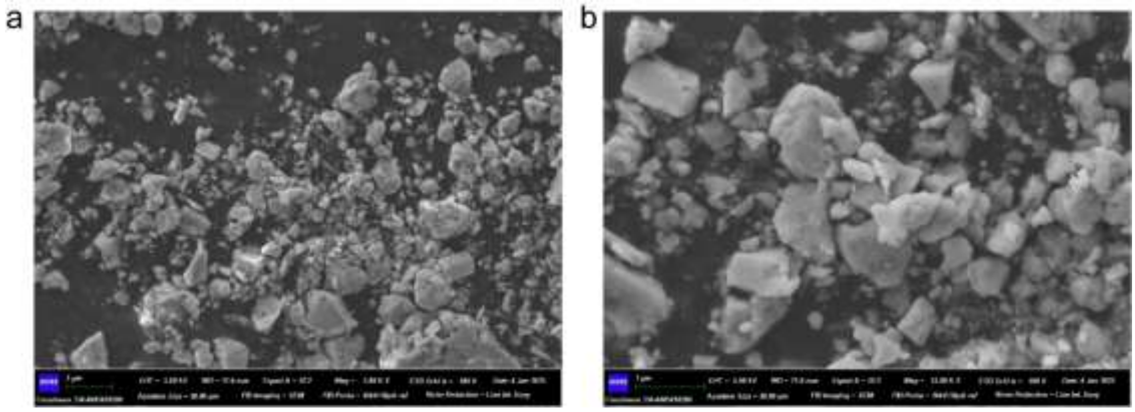

Fig. S35 SEM images of the LZOC-O HSSE.

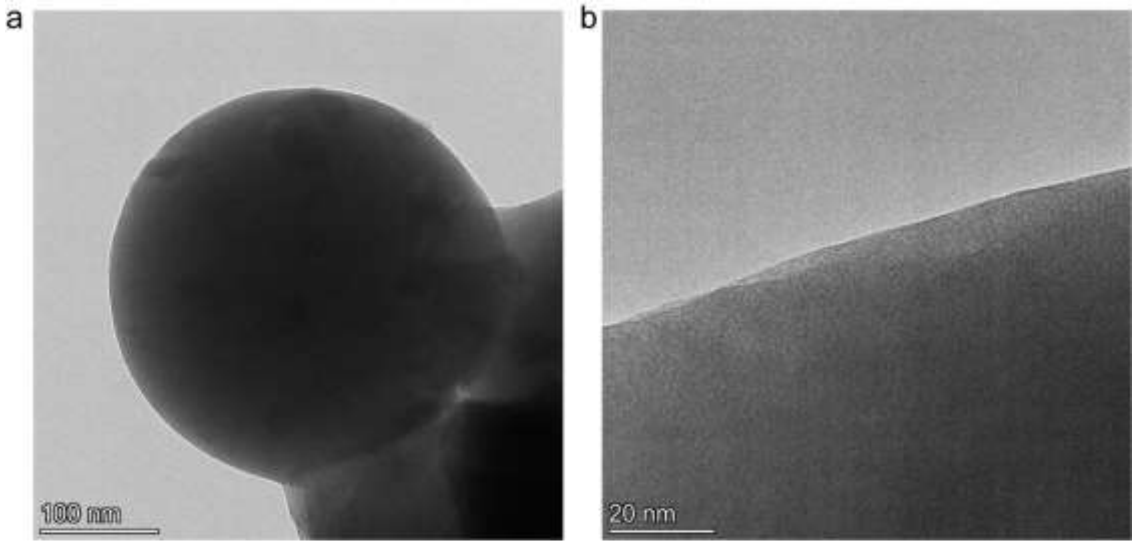

Fig. S36 TEM images of the LZOC-O HSSE.

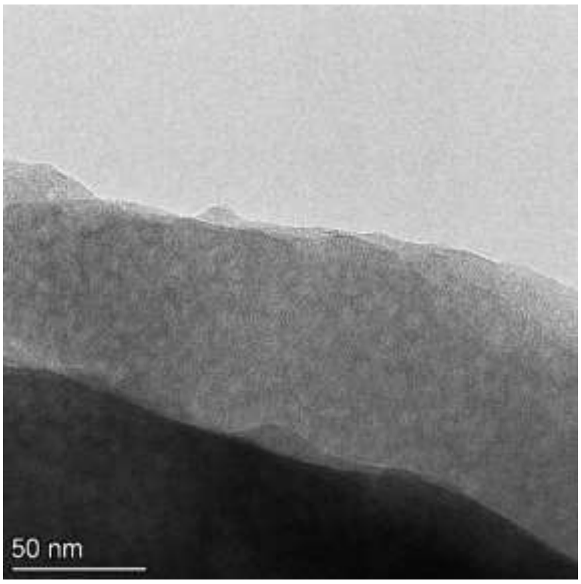

Fig. S37 HRTEM image of LZOC-H HSSE interphase synthesis under CO_2-rich conditions.

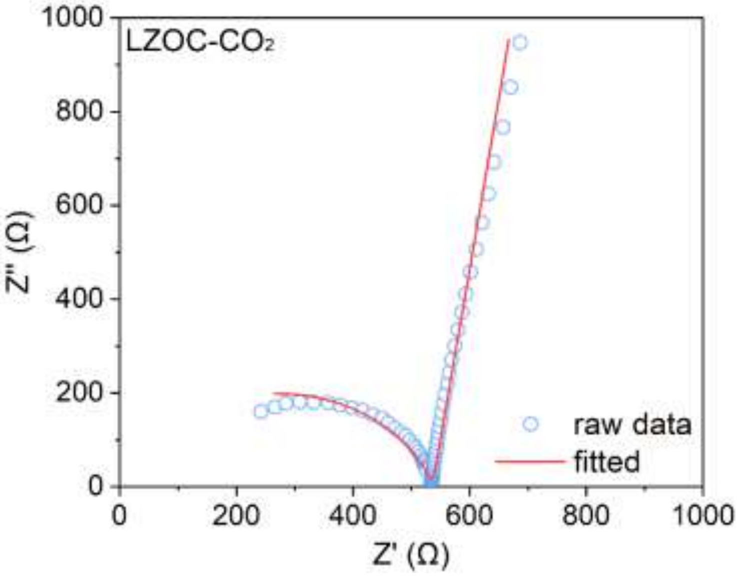

Fig. S38 Nyquist plots of LZOC-H HSSE synthesis under CO_2-rich conditions.

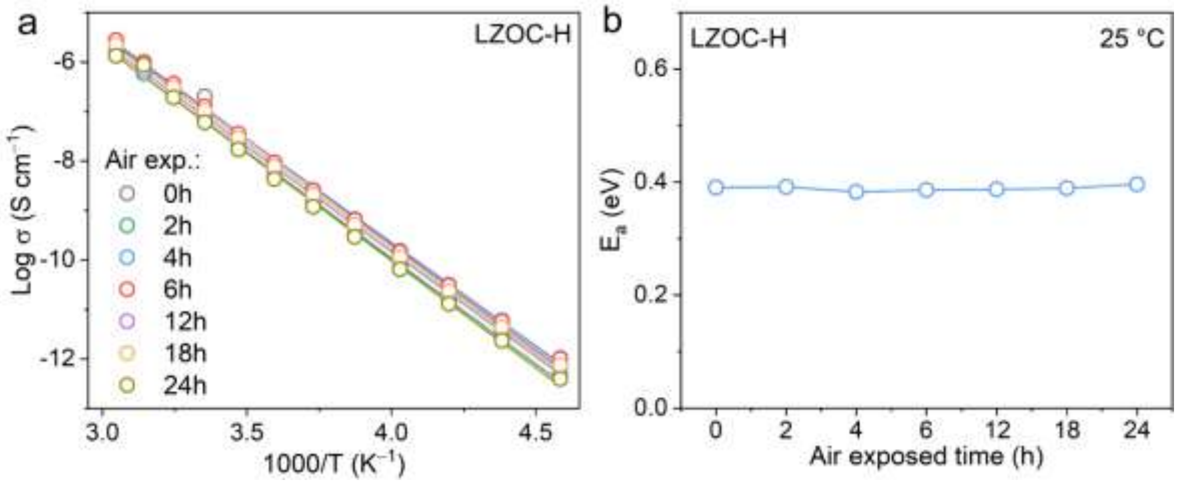

Fig. S39 (a) Arrhenius plots and (b) Active energies of LZOC-H HSSE after differnt air exposure times at 25 ℃.

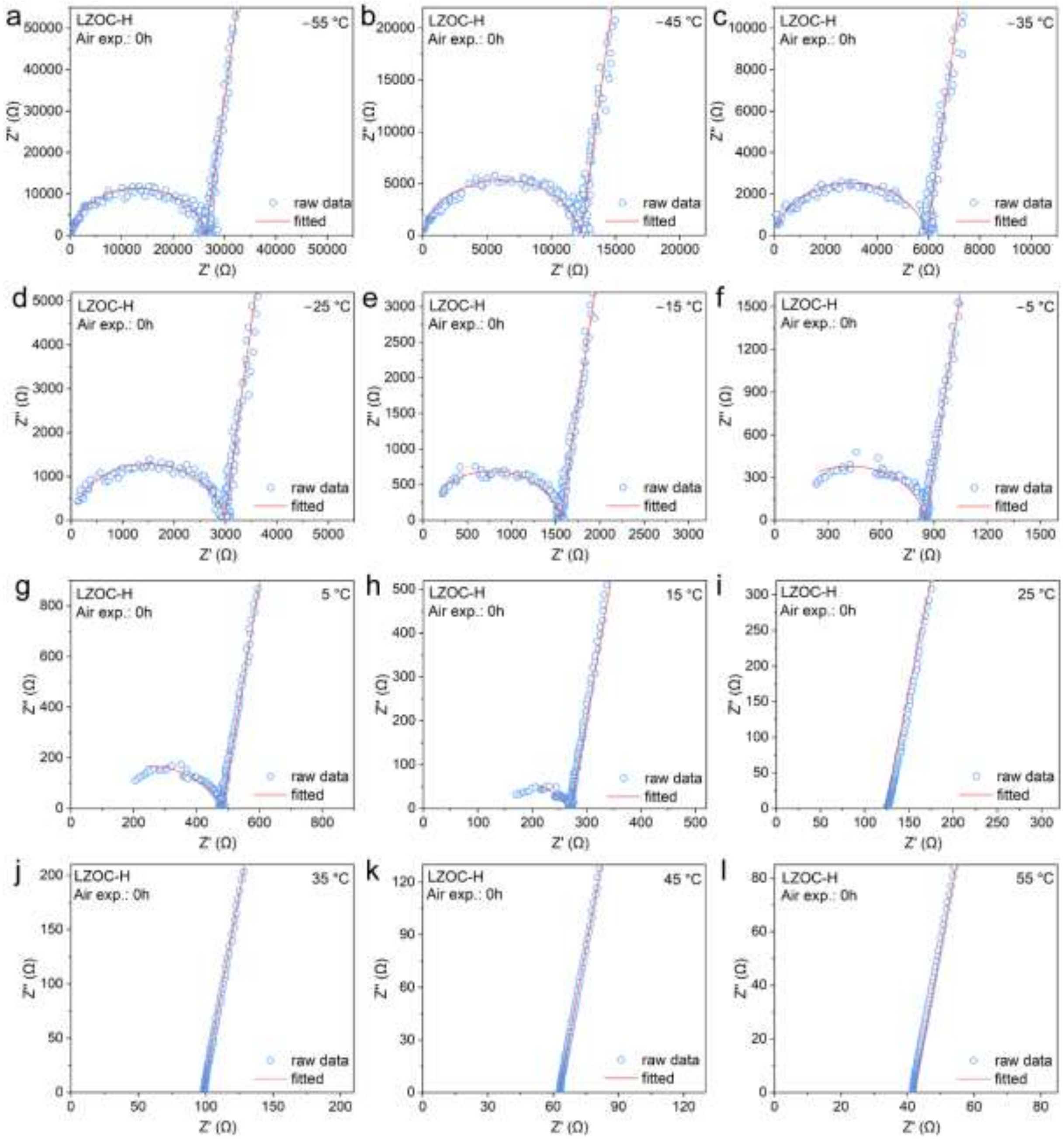

Fig. S40 Nyquist plots of LZOC-H HSSE measured immediately after conditioning in a dry room 0 h at various temperatures: (a) −55 ℃, (b) −45 ℃, (c) −35 ℃, (d) −25 ℃, (e) −15 ℃, (f) −5 ℃, (g) 5 ℃, (h) 15 ℃, (i) 25 ℃, (j) 35 ℃, (k) 45 ℃, and (l) 55 ℃.

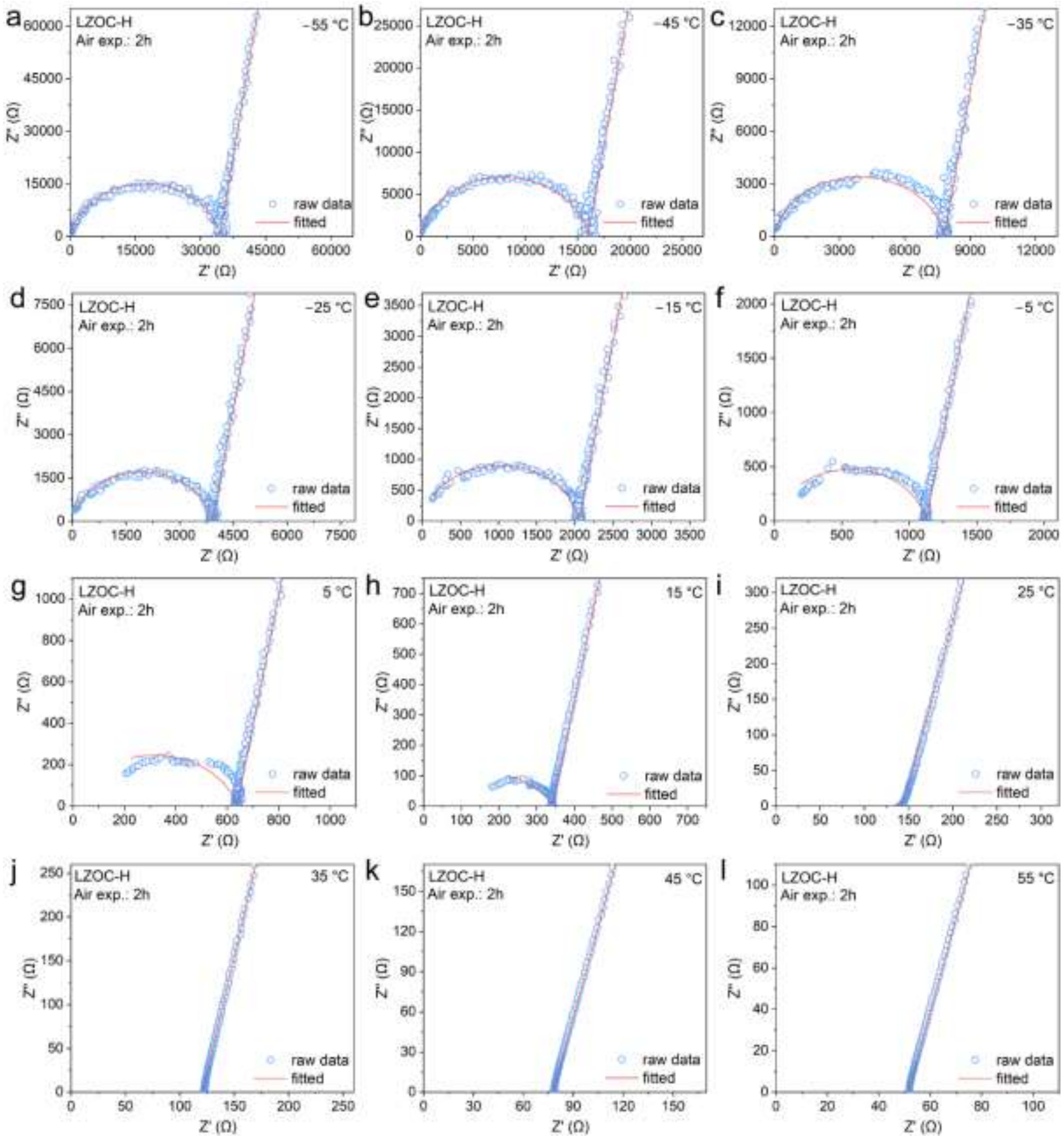

Fig. S41 Nyquist plots of LZOC-H HSSE measured immediately after conditioning in a dry room 2 h at various temperatures: (a) –55 ℃, (b) –45 ℃, (c) –35 ℃, (d) –25 ℃, (e) –15 ℃, (f) –5 ℃, (g) 5 ℃, (h) 15 ℃, (i) 25 ℃, (j) 35 ℃, (k) 45 ℃, and (l) 55 ℃.

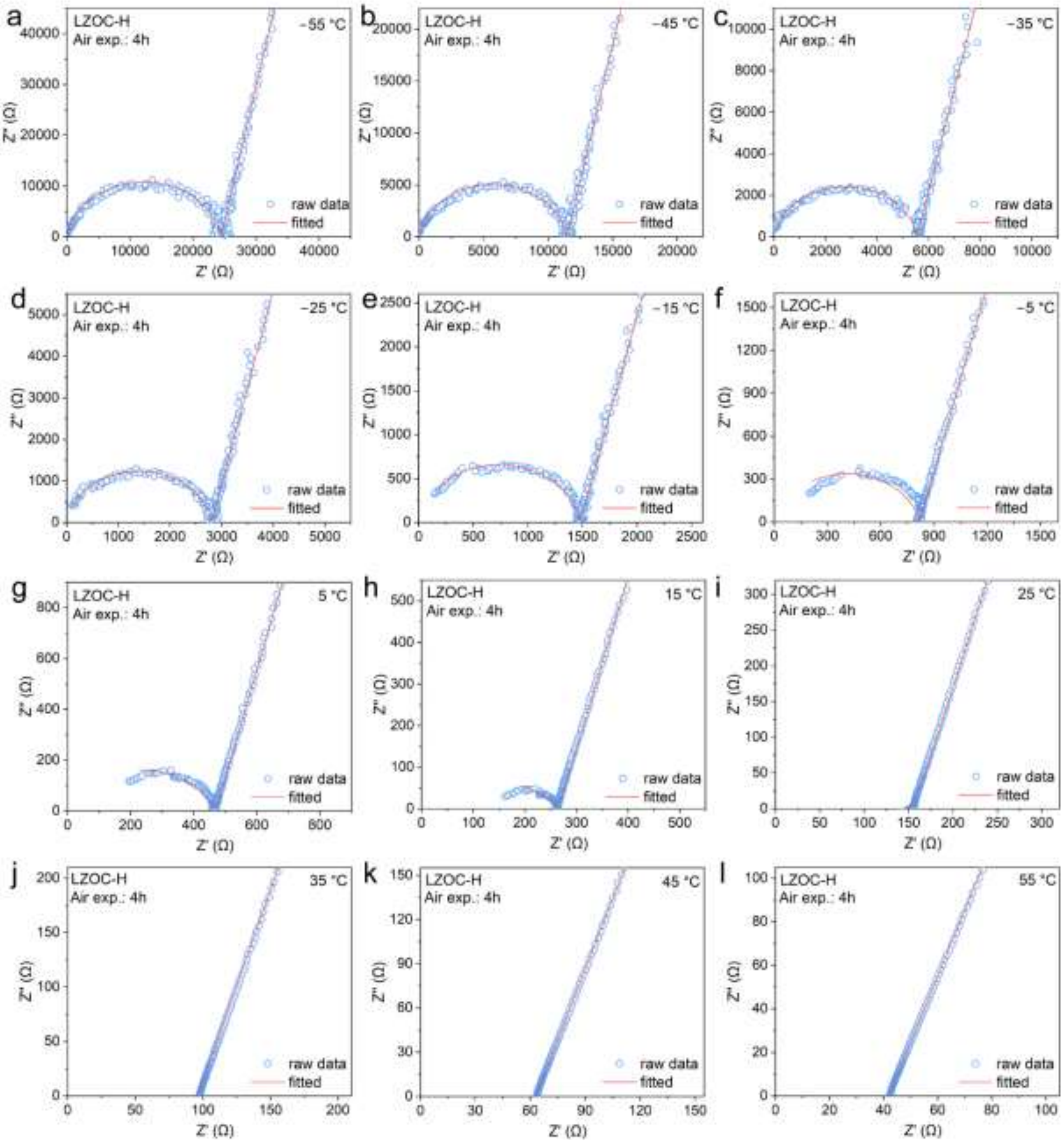

Fig. S42 Nyquist plots of LZOC-H HSSE measured immediately after conditioning in a dry room 4 h at various temperatures: (a) –55 ℃, (b) –45 ℃, (c) –35 ℃, (d) –25 ℃, (e) –15 ℃, (f) –5 ℃, (g) 5 ℃, (h) 15 ℃, (i) 25 ℃, (j) 35 ℃, (k) 45 ℃, and (l) 55 ℃.

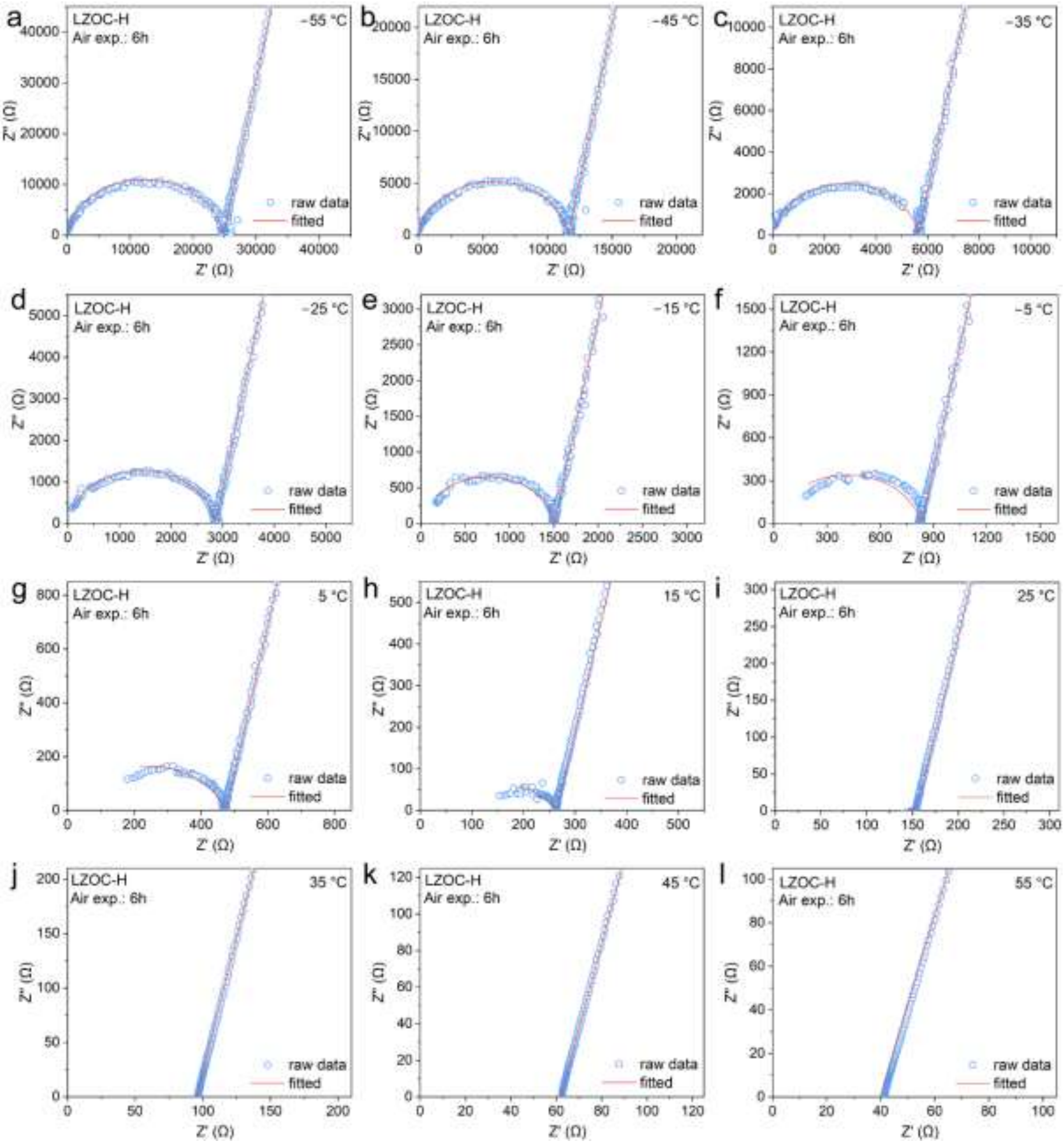

Fig. S43 Nyquist plots of LZOC-H HSSE measured immediately after conditioning in a dry room 6 h at various temperatures: (a) –55 ℃, (b) –45 ℃, (c) –35 ℃, (d) –25 ℃, (e) –15 ℃, (f) –5 ℃, (g) 5 ℃, (h) 15 ℃, (i) 25 ℃, (j) 35 ℃, (k) 45 ℃, and (l) 55 ℃.

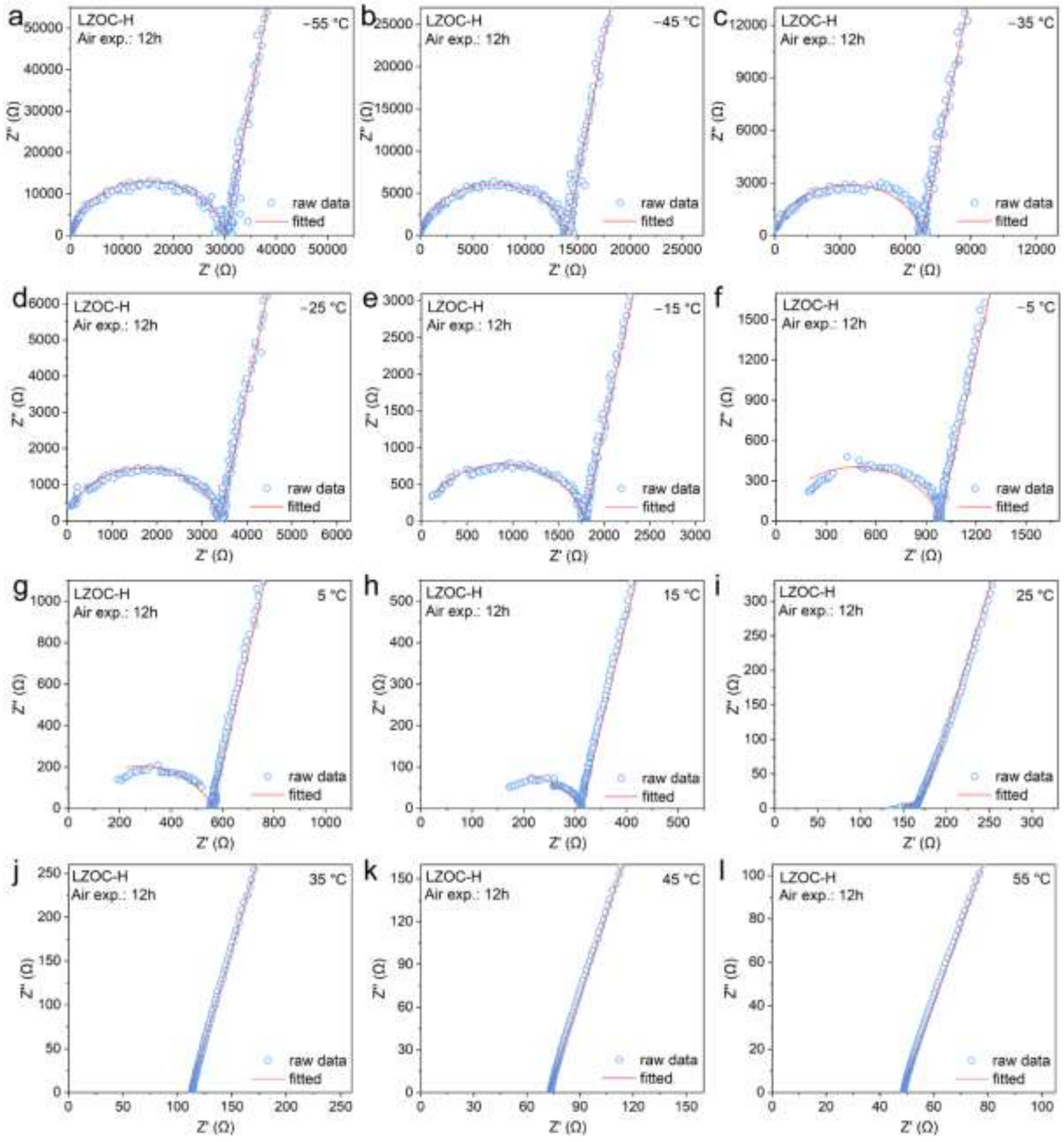

Fig. S44 Nyquist plots of LZOC-H HSSE measured immediately after conditioning in a dry room 12 h at various temperatures: (a) –55 ℃, (b) –45 ℃, (c) –35 ℃, (d) –25 ℃, (e) –15 ℃, (f) –5 ℃, (g) 5 ℃, (h) 15 ℃, (i) 25 ℃, (j) 35 ℃, (k) 45 ℃, and (l) 55 ℃.

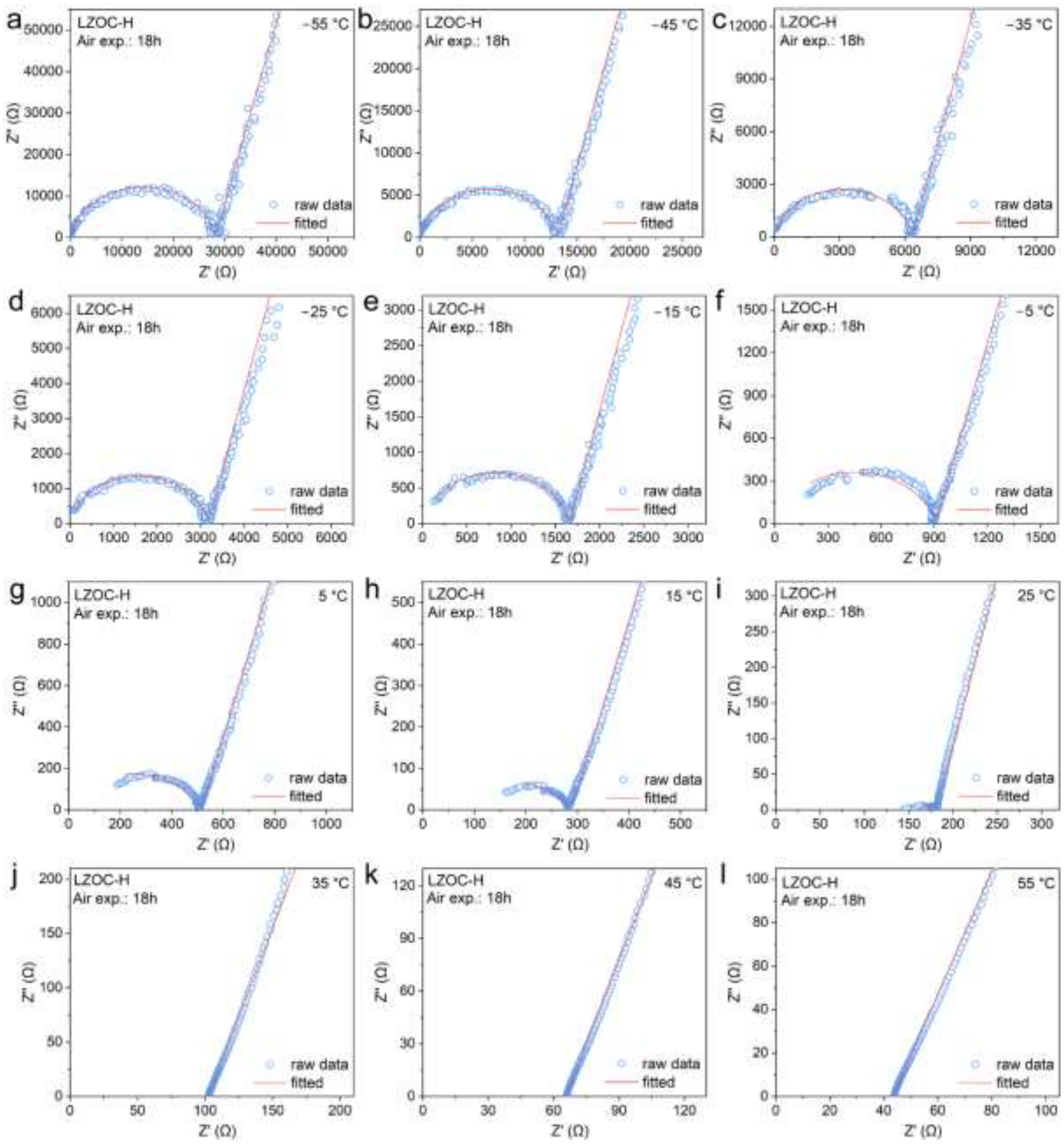

Fig. S45 Nyquist plots of LZOC-H HSSE measured immediately after conditioning in a dry room 18 h at various temperatures: (a) –55 ℃, (b) –45 ℃, (c) –35 ℃, (d) –25 ℃, (e) –15 ℃, (f) –5 ℃, (g) 5 ℃, (h) 15 ℃, (i) 25 ℃, (j) 35 ℃, (k) 45 ℃, and (l) 55 ℃.

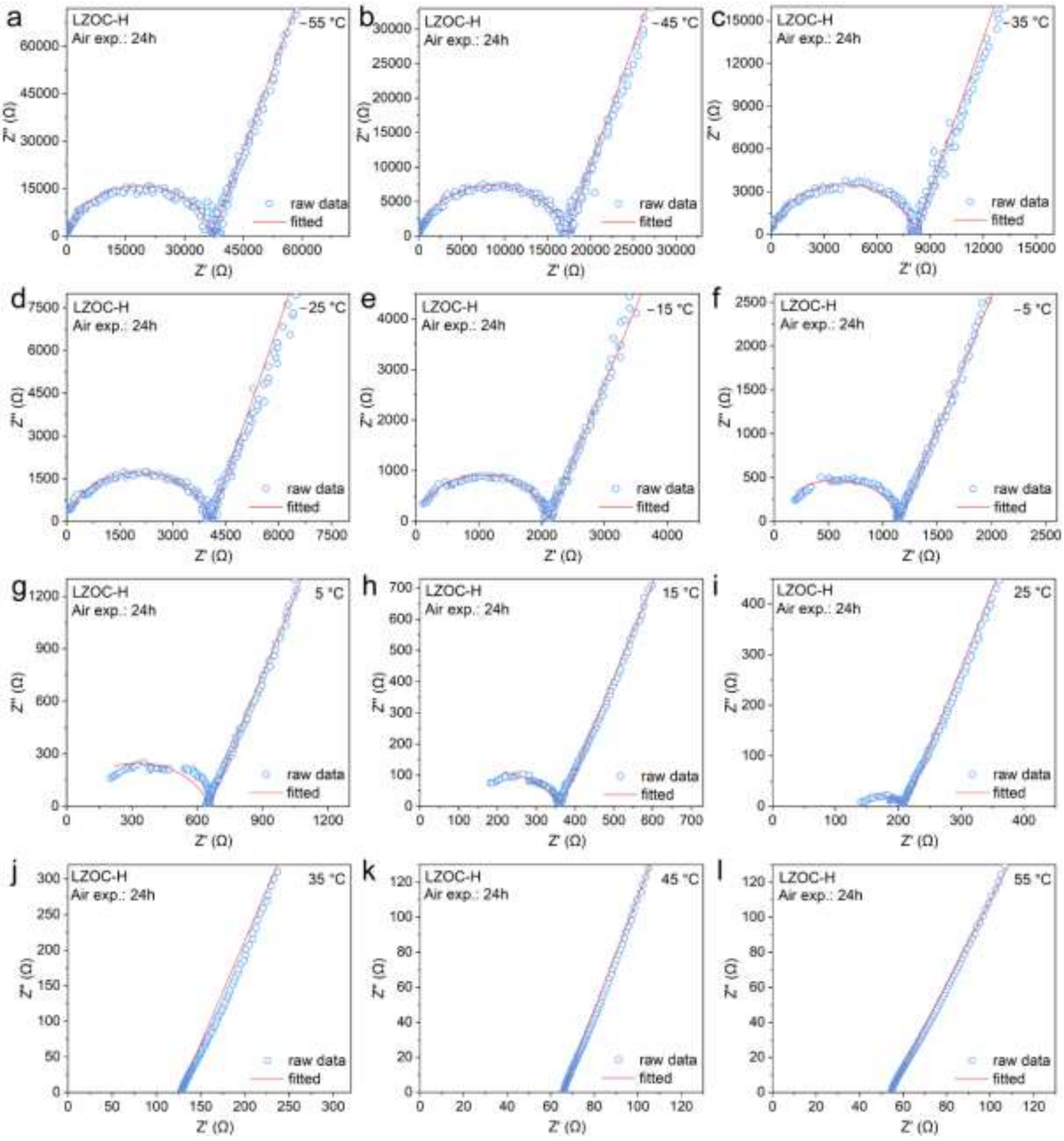

Fig. S46 Nyquist plots of LZOC-H HSSE measured immediately after conditioning in a dry room 24 h at various temperatures: (a) –55 ℃, (b) –45 ℃, (c) –35 ℃, (d) –25 ℃, (e) –15 ℃, (f) –5 ℃, (g) 5 ℃, (h) 15 ℃, (i) 25 ℃, (j) 35 ℃, (k) 45 ℃, and (l) 55 ℃.

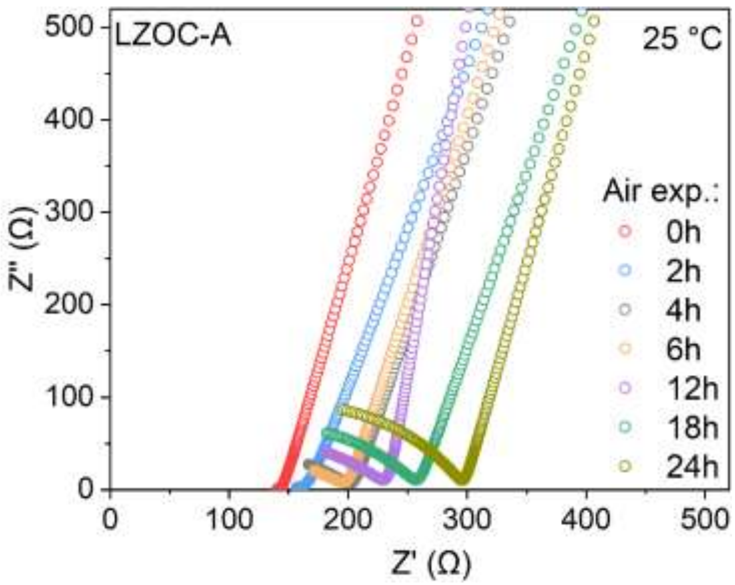

Fig. S47 Nyquist plots of LZOC-A HSSE after different air exposure times.

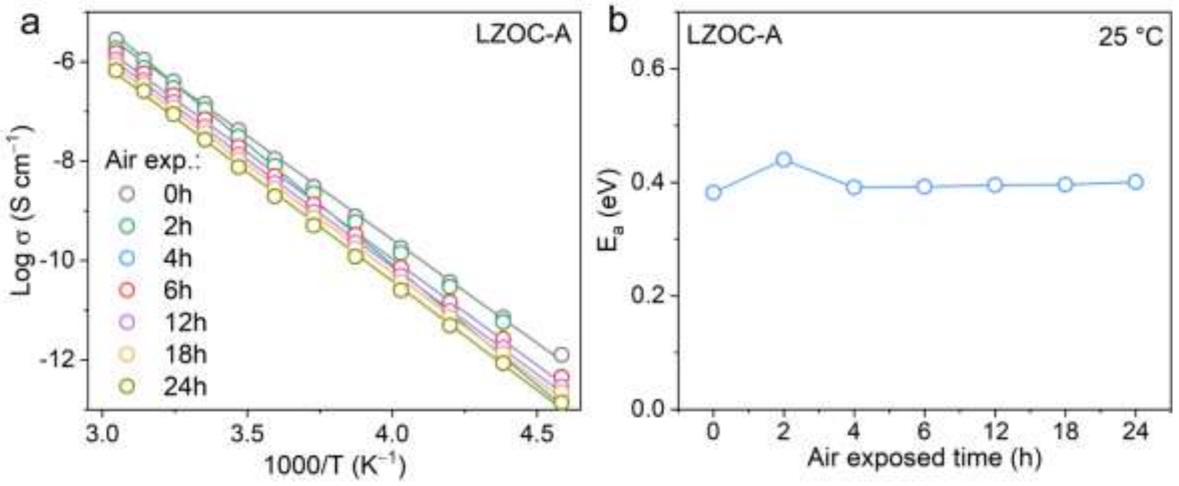

Fig. S48 (a) Arrhenius plots and (b) Active energies of LZOC-A electrolyte after different air exposure times.

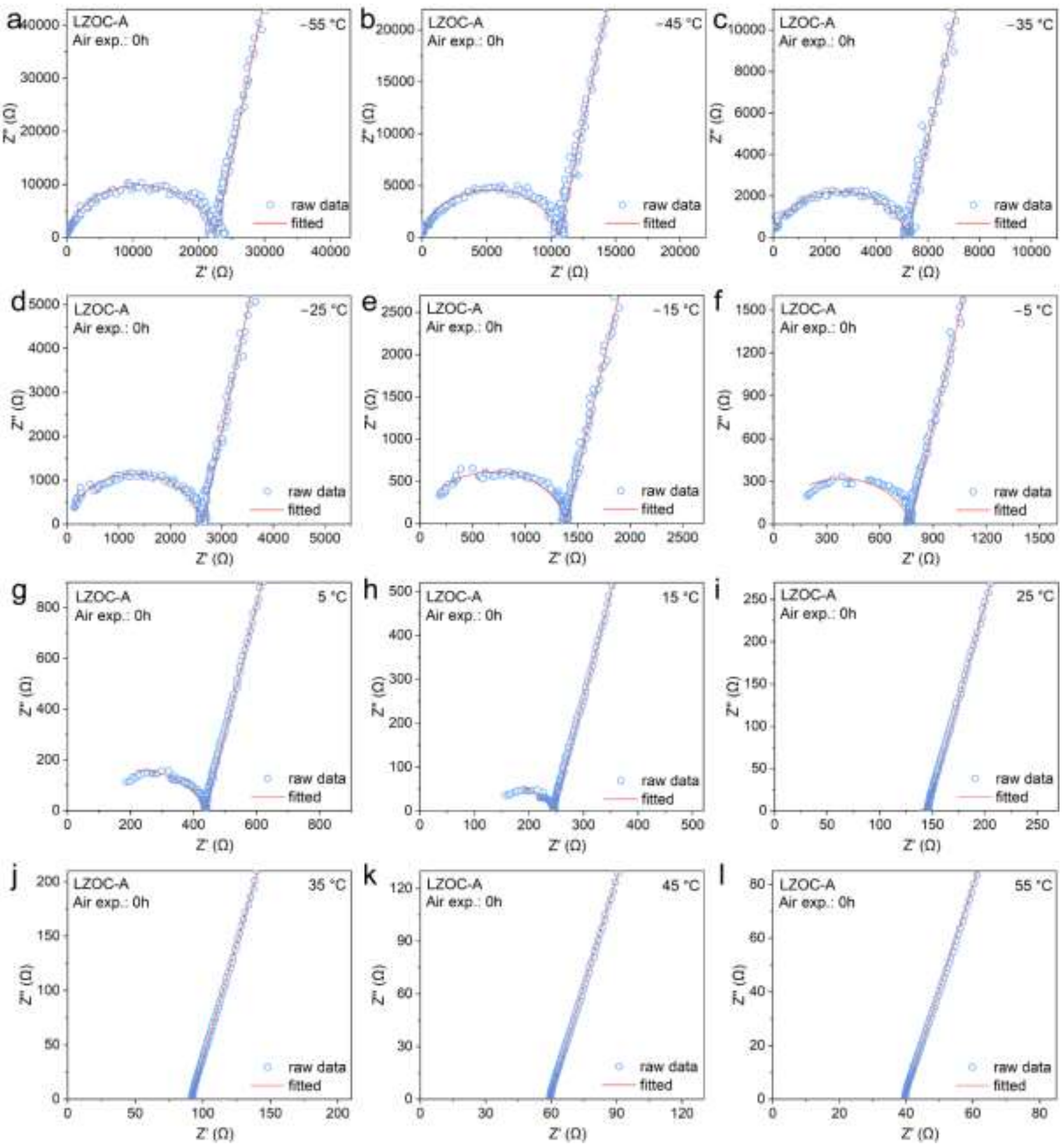

Fig. S49 Nyquist plots of LZOC-A HSSE measured immediately after conditioning in a dry room 0 h at various temperatures: (a) –55 ℃, (b) –45 ℃, (c) –35 ℃, (d) –25 ℃, (e) –15 ℃, (f) –5 ℃, (g) 5 ℃, (h) 15 ℃, (i) 25 ℃, (j) 35 ℃, (k) 45 ℃, and (l) 55 ℃.

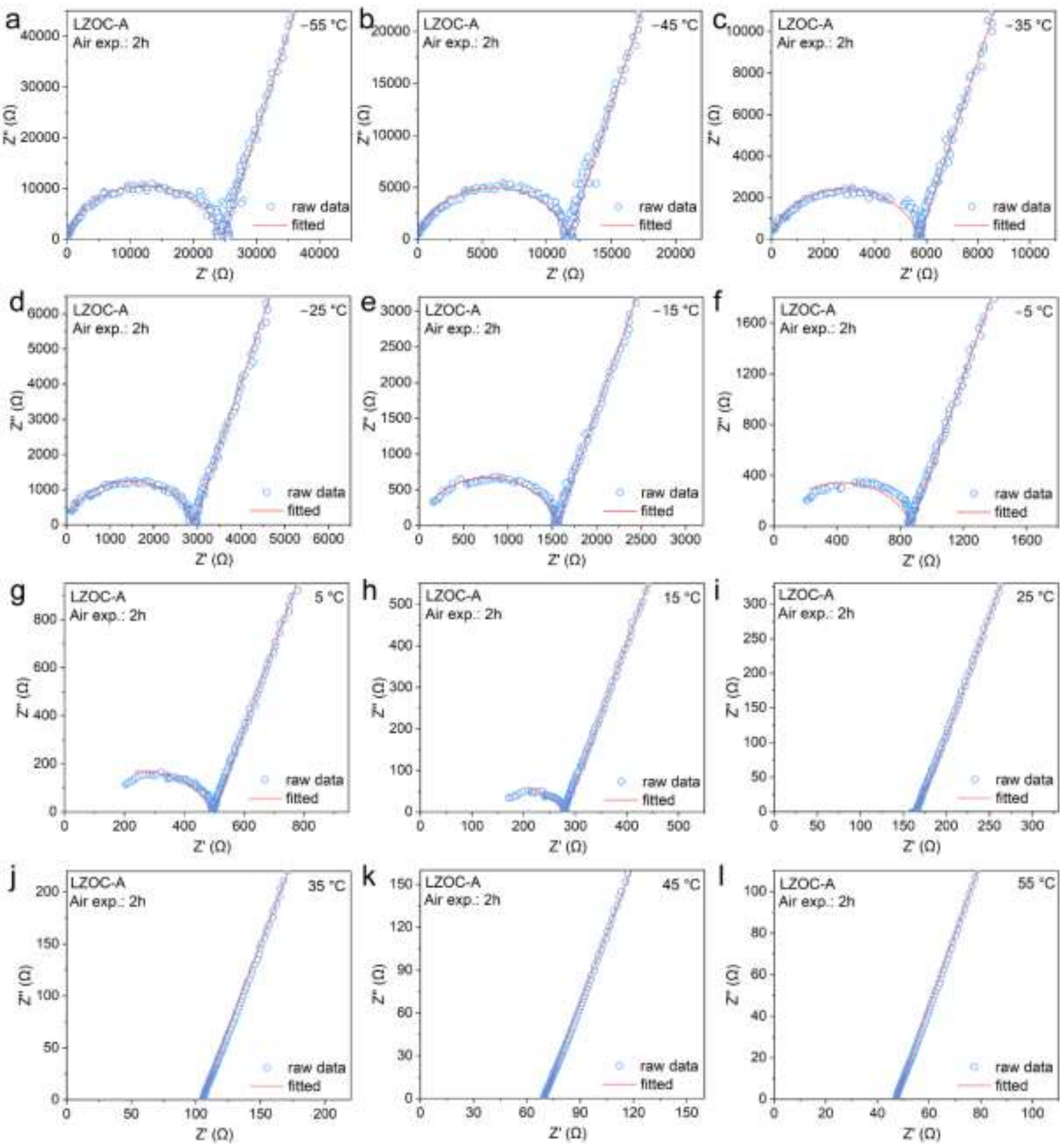

Fig. S50 Nyquist plots of LZOC-A HSSE measured immediately after conditioning in a dry room 2 h at various temperatures: (a) –55 ℃, (b) –45 ℃, (c) –35 ℃, (d) –25 ℃, (e) –15 ℃, (f) –5 ℃, (g) 5 ℃, (h) 15 ℃, (i) 25 ℃, (j) 35 ℃, (k) 45 ℃, and (l) 55 ℃.

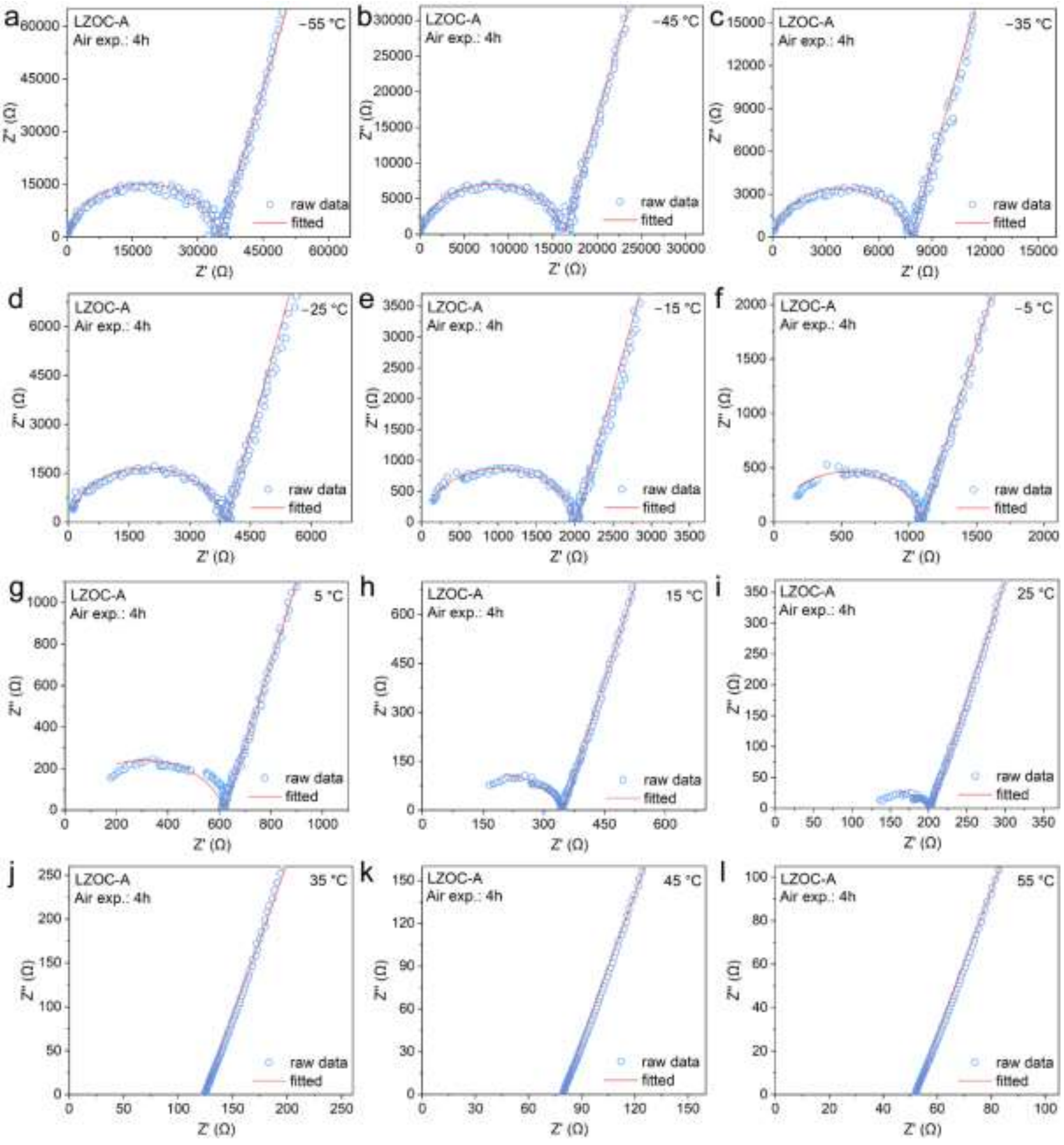

Fig. S51 Nyquist plots of LZOC-A HSSE measured immediately after conditioning in a dry room 4 h at various temperatures: (a) –55 ℃, (b) –45 ℃, (c) –35 ℃, (d) –25 ℃, (e) –15 ℃, (f) –5 ℃, (g) 5 ℃, (h) 15 ℃, (i) 25 ℃, (j) 35 ℃, (k) 45 ℃, and (l) 55 ℃.

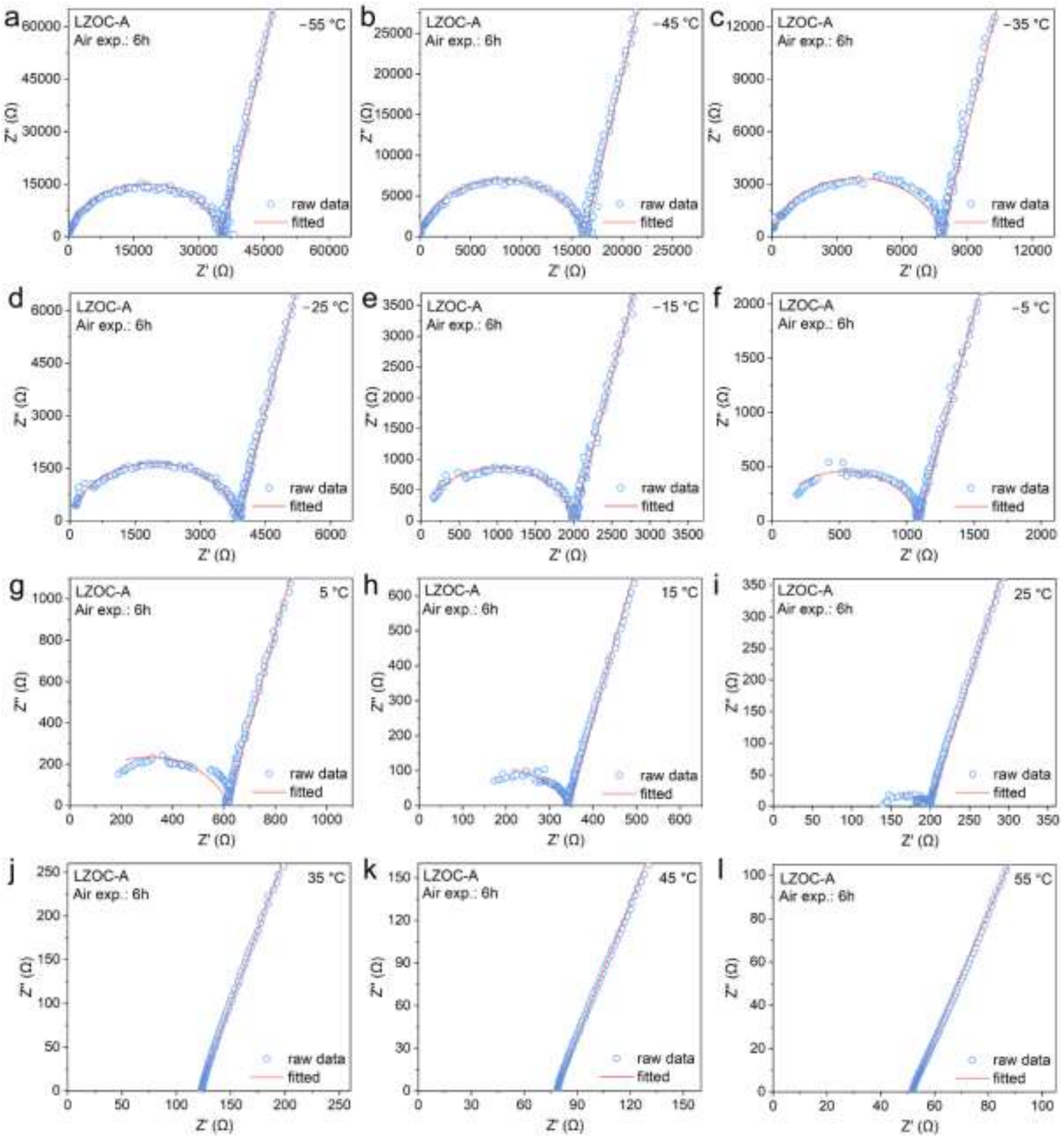

Fig. S52 Nyquist plots of LZOC-A HSSE measured immediately after conditioning in a dry room 6 h at various temperatures: (a) –55 ℃, (b) –45 ℃, (c) –35 ℃, (d) –25 ℃, (e) –15 ℃, (f) –5 ℃, (g) 5 ℃, (h) 15 ℃, (i) 25 ℃, (j) 35 ℃, (k) 45 ℃, and (l) 55 ℃.

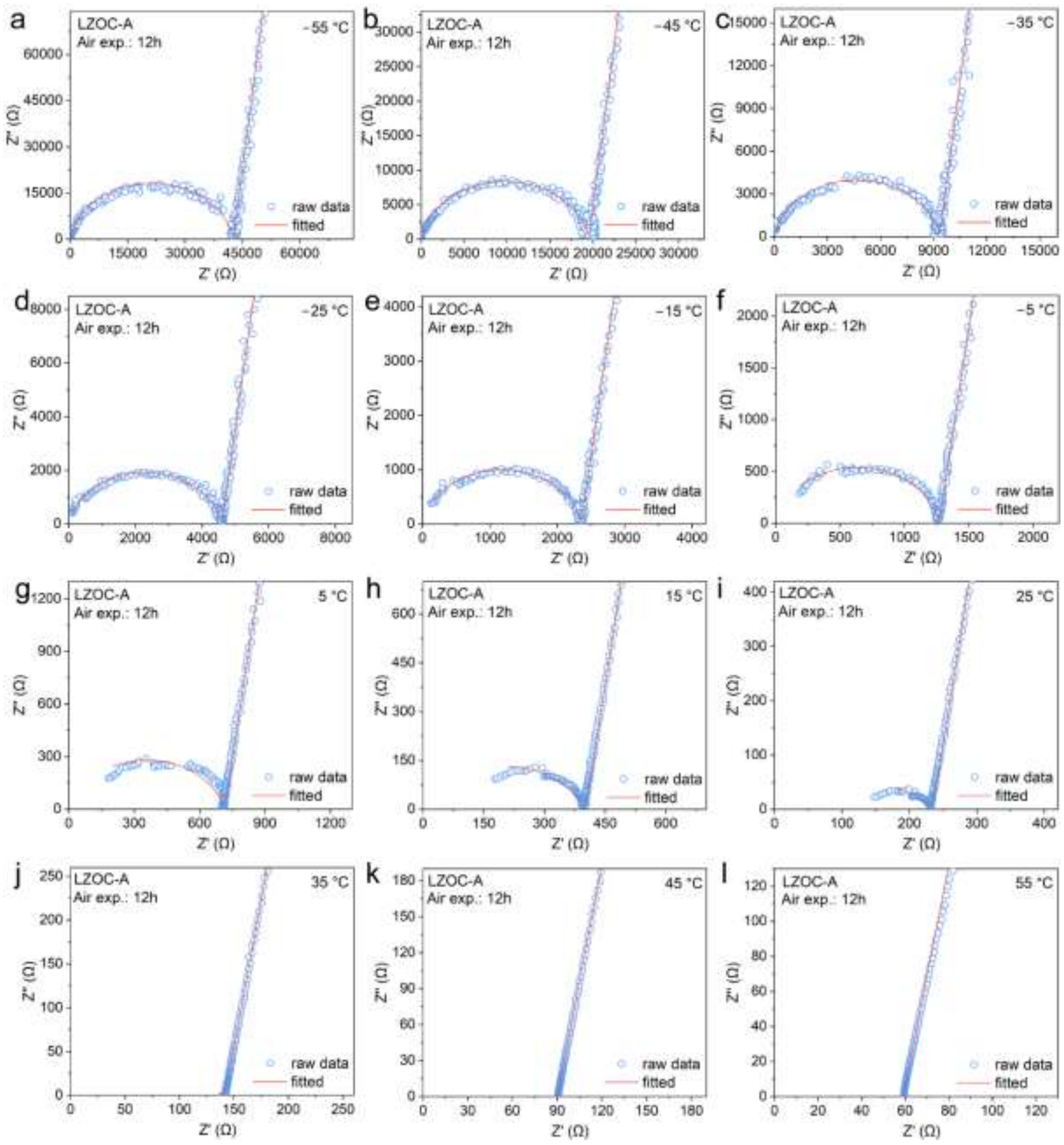

Fig. S53 Nyquist plots of LZOC-A HSSE measured immediately after conditioning in a dry room 12 h at various temperatures: (a) –55 ℃, (b) –45 ℃, (c) –35 ℃, (d) –25 ℃, (e) –15 ℃, (f) –5 ℃, (g) 5 ℃, (h) 15 ℃, (i) 25 ℃, (j) 35 ℃, (k) 45 ℃, and (l) 55 ℃.

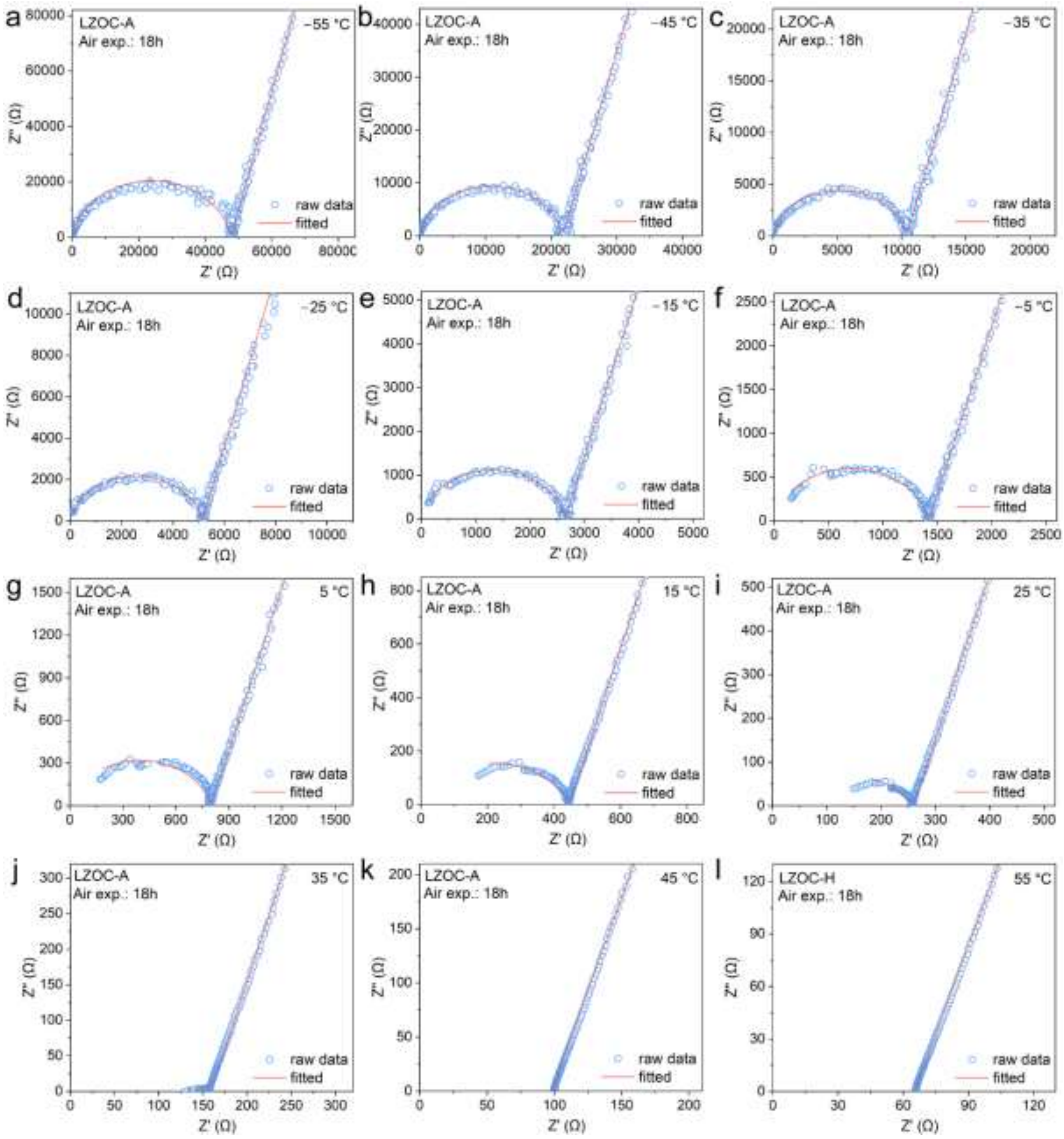

Fig. S54 Nyquist plots of LZOC-A HSSE measured immediately after conditioning in a dry room 18 h at various temperatures: (a) –55 ℃, (b) –45 ℃, (c) –35 ℃, (d) –25 ℃, (e) –15 ℃, (f) –5 ℃, (g) 5 ℃, (h) 15 ℃, (i) 25 ℃, (j) 35 ℃, (k) 45 ℃, and (l) 55 ℃.

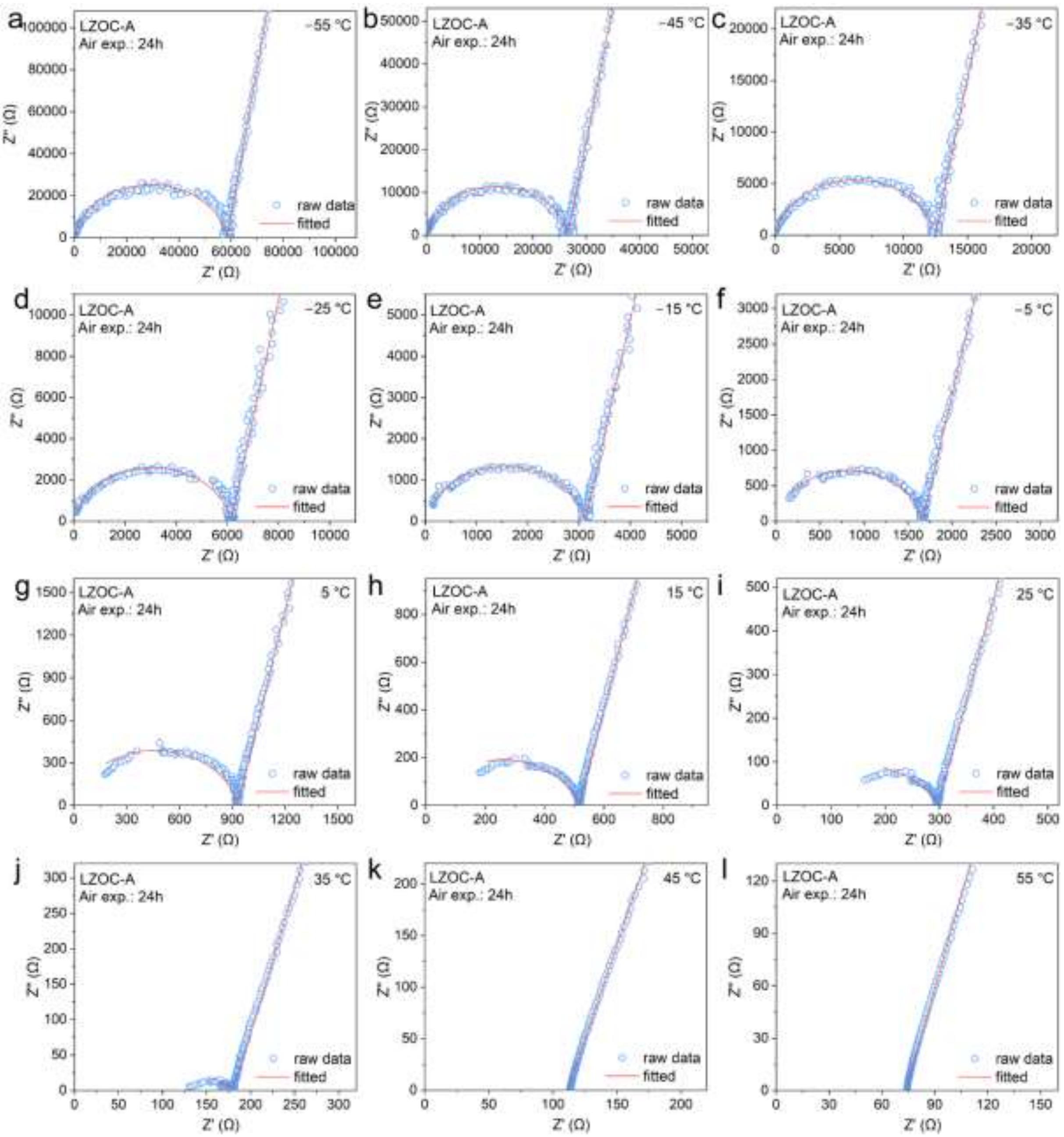

Fig. S55 Nyquist plots of LZOC-A HSSE measured immediately after conditioning in a dry room 24 h at various temperatures: (a) –55 ℃, (b) –45 ℃, (c) –35 ℃, (d) –25 ℃, (e) –15 ℃, (f) –5 ℃, (g) 5 ℃, (h) 15 ℃, (i) 25 ℃, (j) 35 ℃, (k) 45 ℃, and (l) 55 ℃.

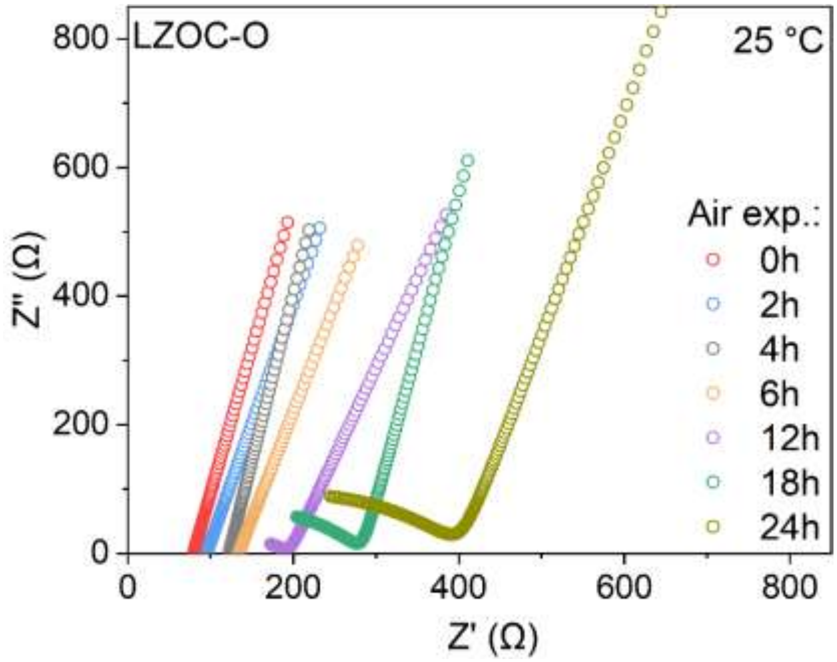

Fig. S56 Nyquist plots of LZOC-O HSSE after different air exposure times.

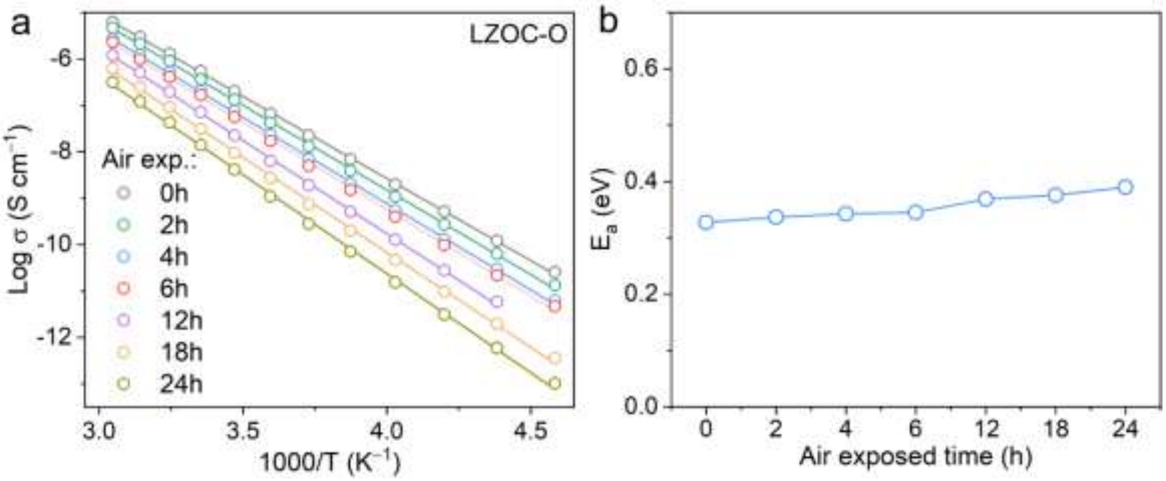

Fig. S57 (a) Arrhenius plots and (b) Active energies of LZOC-O electrolyte after different air exposure times.

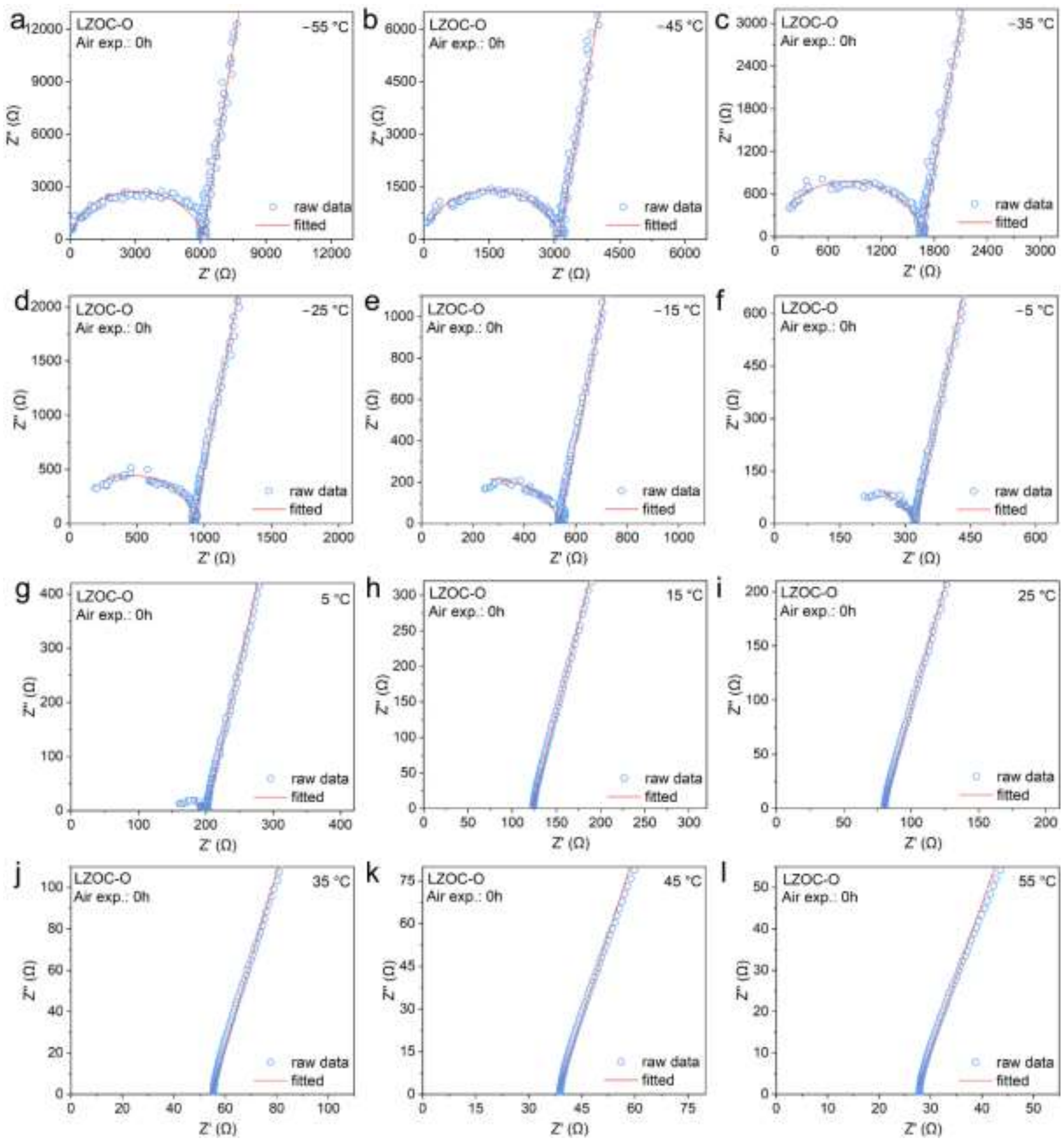

Fig. S58 Nyquist plots of LZOC-O HSSE measured immediately after conditioning in a dry room 0 h at various temperatures: (a) –55 ℃, (b) –45 ℃, (c) –35 ℃, (d) –25 ℃, (e) –15 ℃, (f) –5 ℃, (g) 5 ℃, (h) 15 ℃, (i) 25 ℃, (j) 35 ℃, (k) 45 ℃, and (l) 55 ℃.

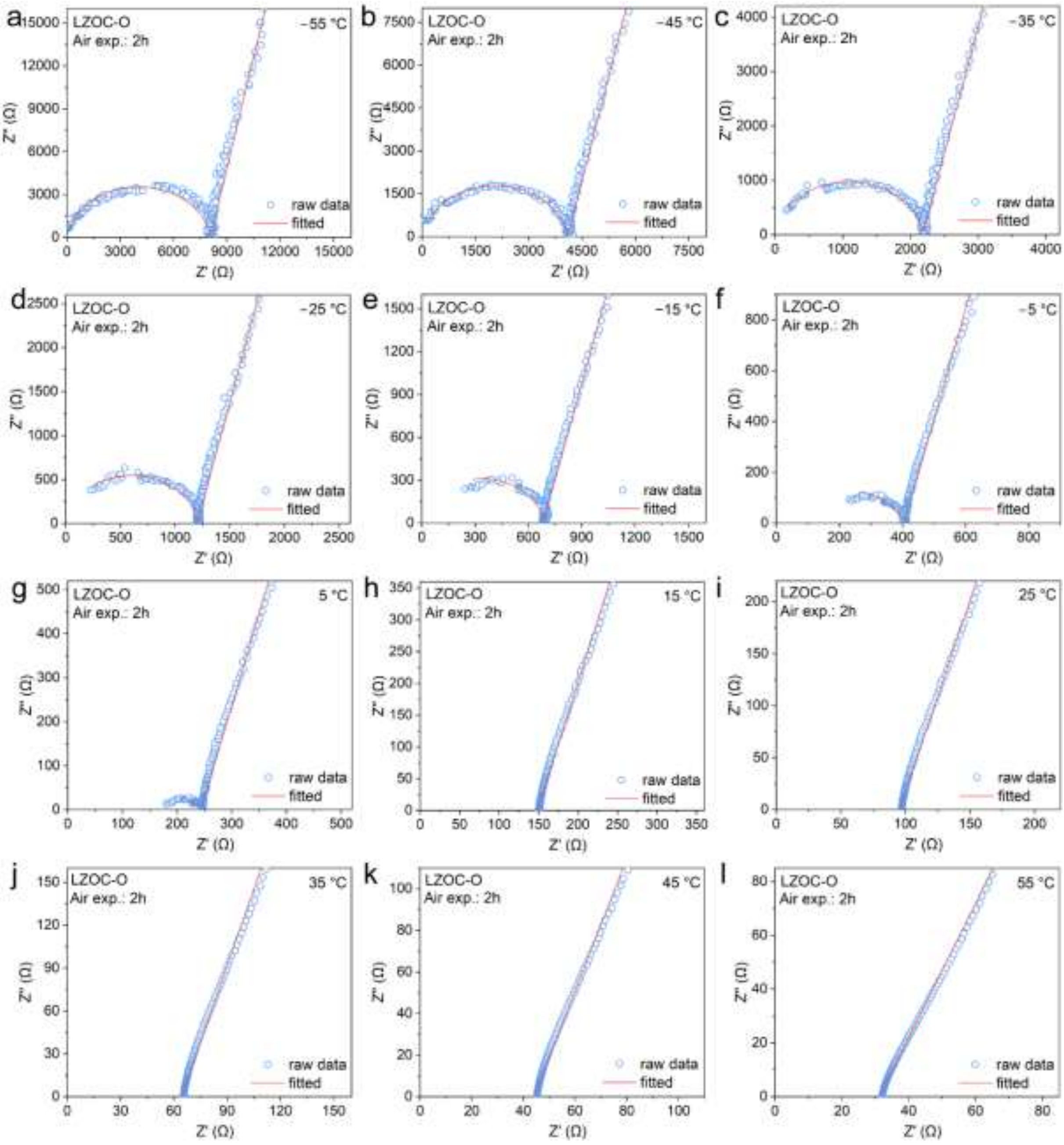

Fig. S59 Nyquist plots of LZOC-O HSSE measured immediately after conditioning in a dry room 2 h at various temperatures: (a) –55 ℃, (b) –45 ℃, (c) –35 ℃, (d) –25 ℃, (e) –15 ℃, (f) –5 ℃, (g) 5 ℃, (h) 15 ℃, (i) 25 ℃, (j) 35 ℃, (k) 45 ℃, and (l) 55 ℃.

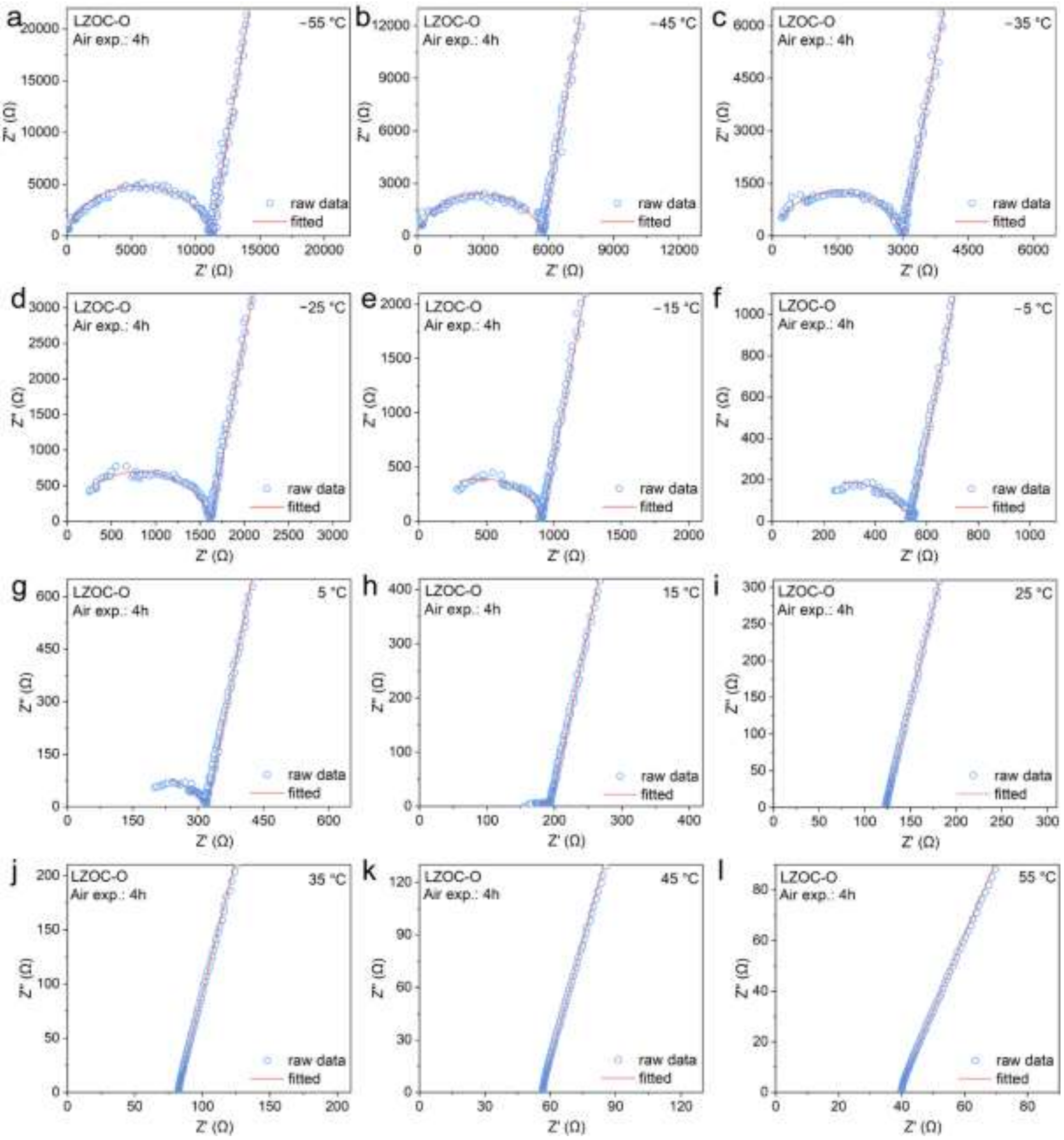

Fig. S60 Nyquist plots of LZOC-O HSSE measured immediately after conditioning in a dry room 4 h at various temperatures: (a) –55 ℃, (b) –45 ℃, (c) –35 ℃, (d) –25 ℃, (e) –15 ℃, (f) –5 ℃, (g) 5 ℃, (h) 15 ℃, (i) 25 ℃, (j) 35 ℃, (k) 45 ℃, and (l) 55 ℃.

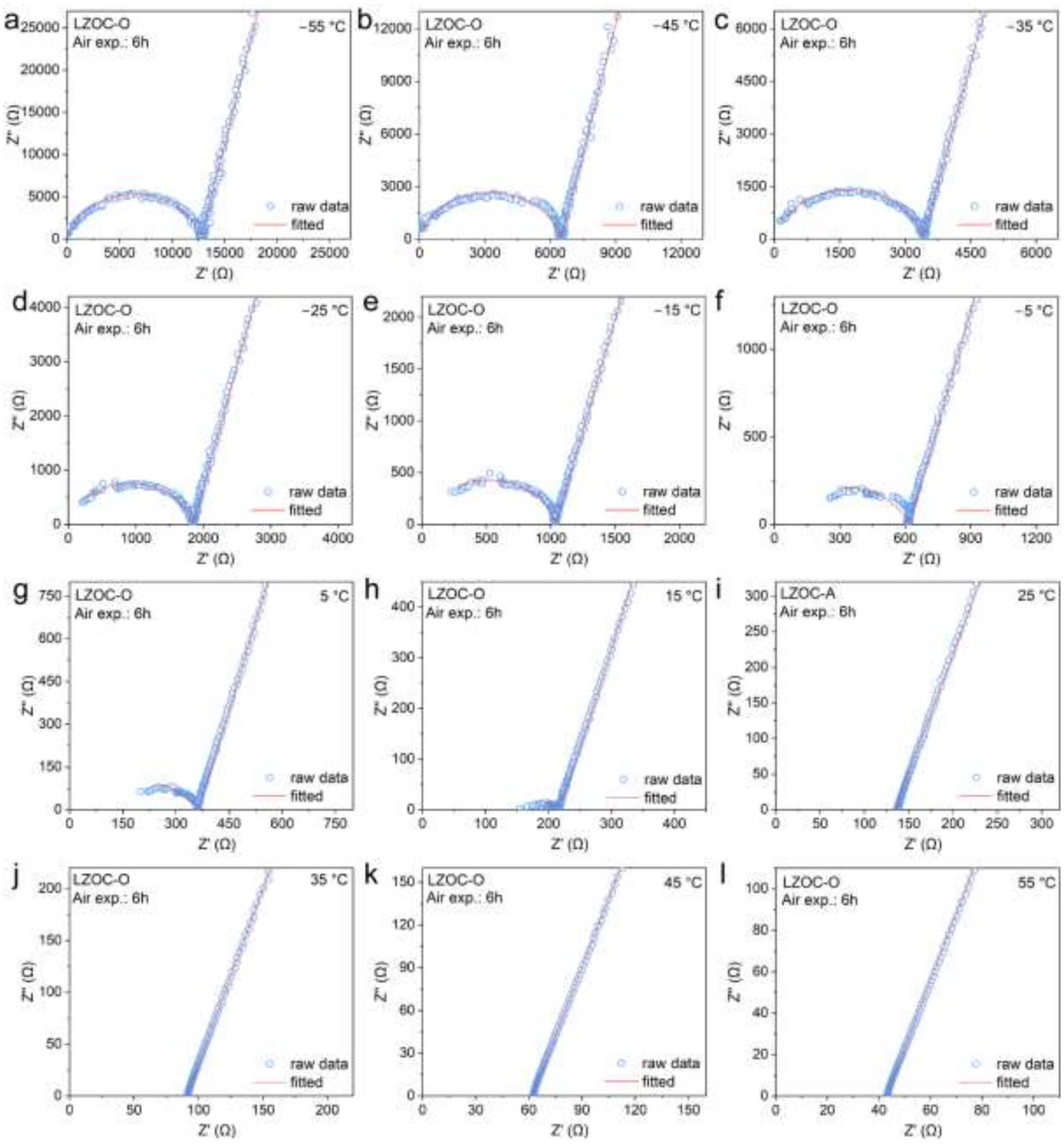

Fig. S61 Nyquist plots of LZOC-O HSSE measured immediately after conditioning in a dry room 6 h at various temperatures: (a) –55 ℃, (b) –45 ℃, (c) –35 ℃, (d) –25 ℃, (e) –15 ℃, (f) –5 ℃, (g) 5 ℃, (h) 15 ℃, (i) 25 ℃, (j) 35 ℃, (k) 45 ℃, and (l) 55 ℃.

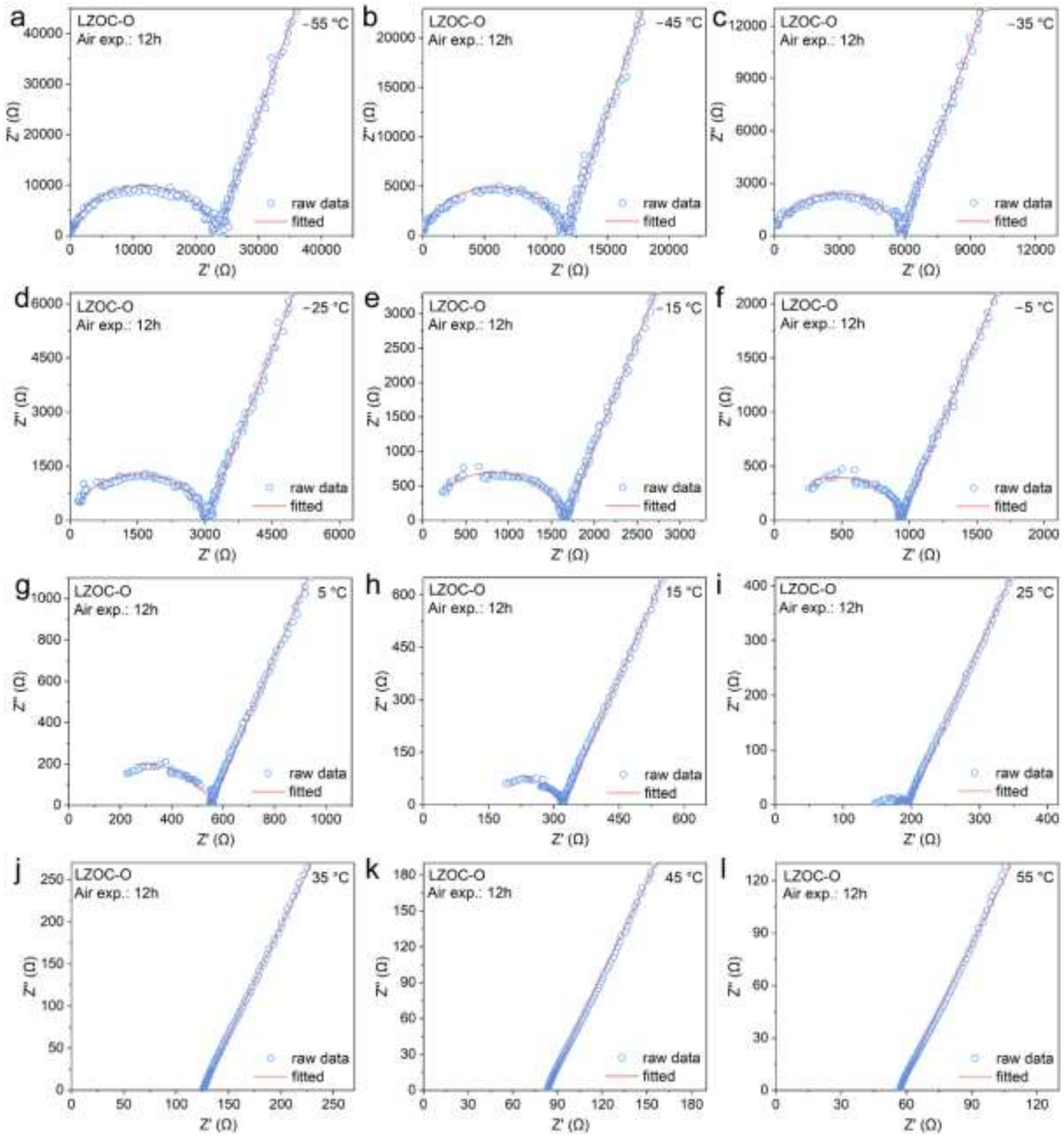

Fig. S62 Nyquist plots of LZOC-O HSSE measured immediately after conditioning in a dry room 12 h at various temperatures: (a) –55 ℃, (b) –45 ℃, (c) –35 ℃, (d) –25 ℃, (e) –15 ℃, (f) –5 ℃, (g) 5 ℃, (h) 15 ℃, (i) 25 ℃, (j) 35 ℃, (k) 45 ℃, and (l) 55 ℃.

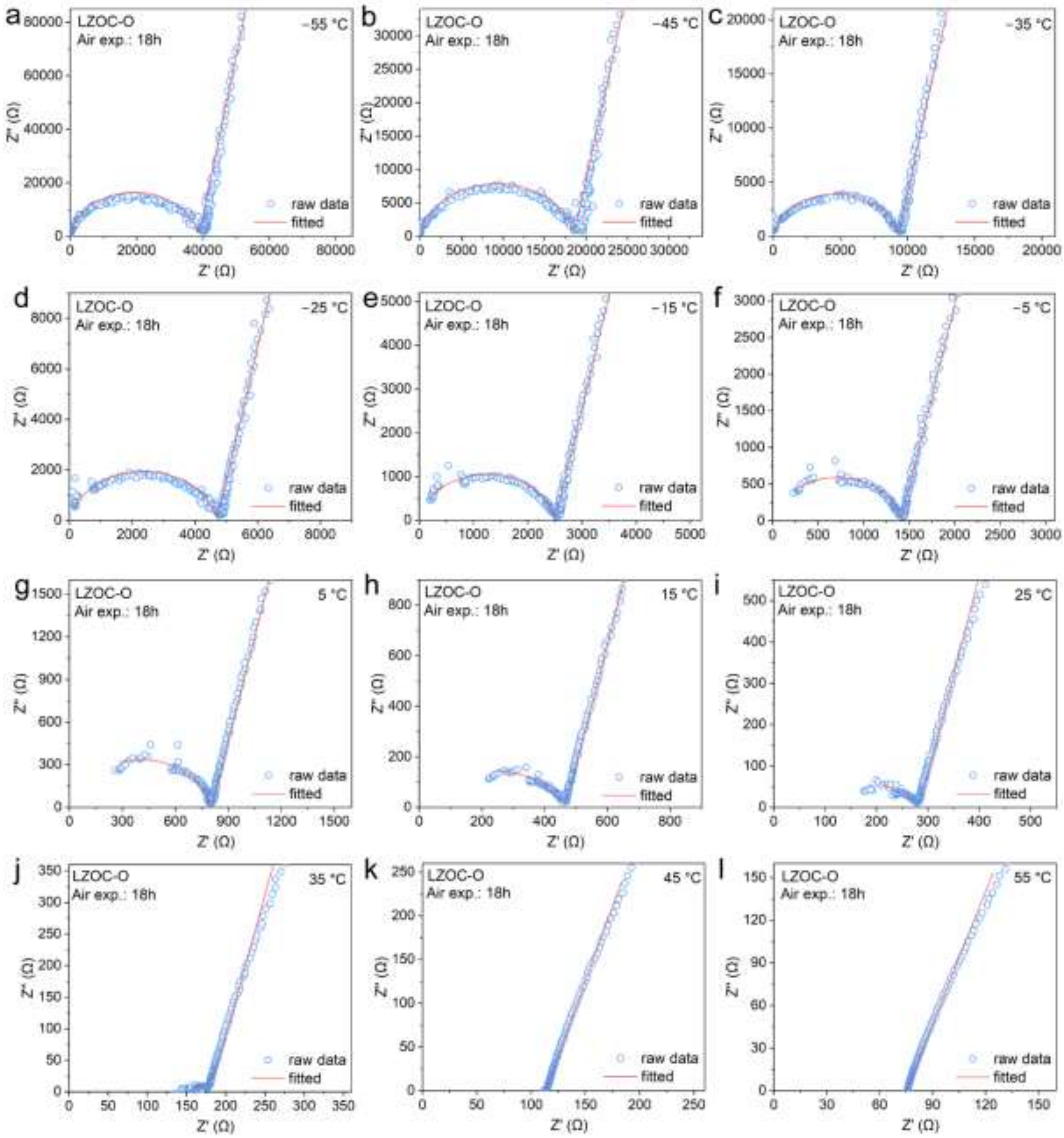

Fig. S63 Nyquist plots of LZOC-O HSSE measured immediately after conditioning in a dry room 18 h at various temperatures: (a) –55 ℃, (b) –45 ℃, (c) –35 ℃, (d) –25 ℃, (e) –15 ℃, (f) –5 ℃, (g) 5 ℃, (h) 15 ℃, (i) 25 ℃, (j) 35 ℃, (k) 45 ℃, and (l) 55 ℃

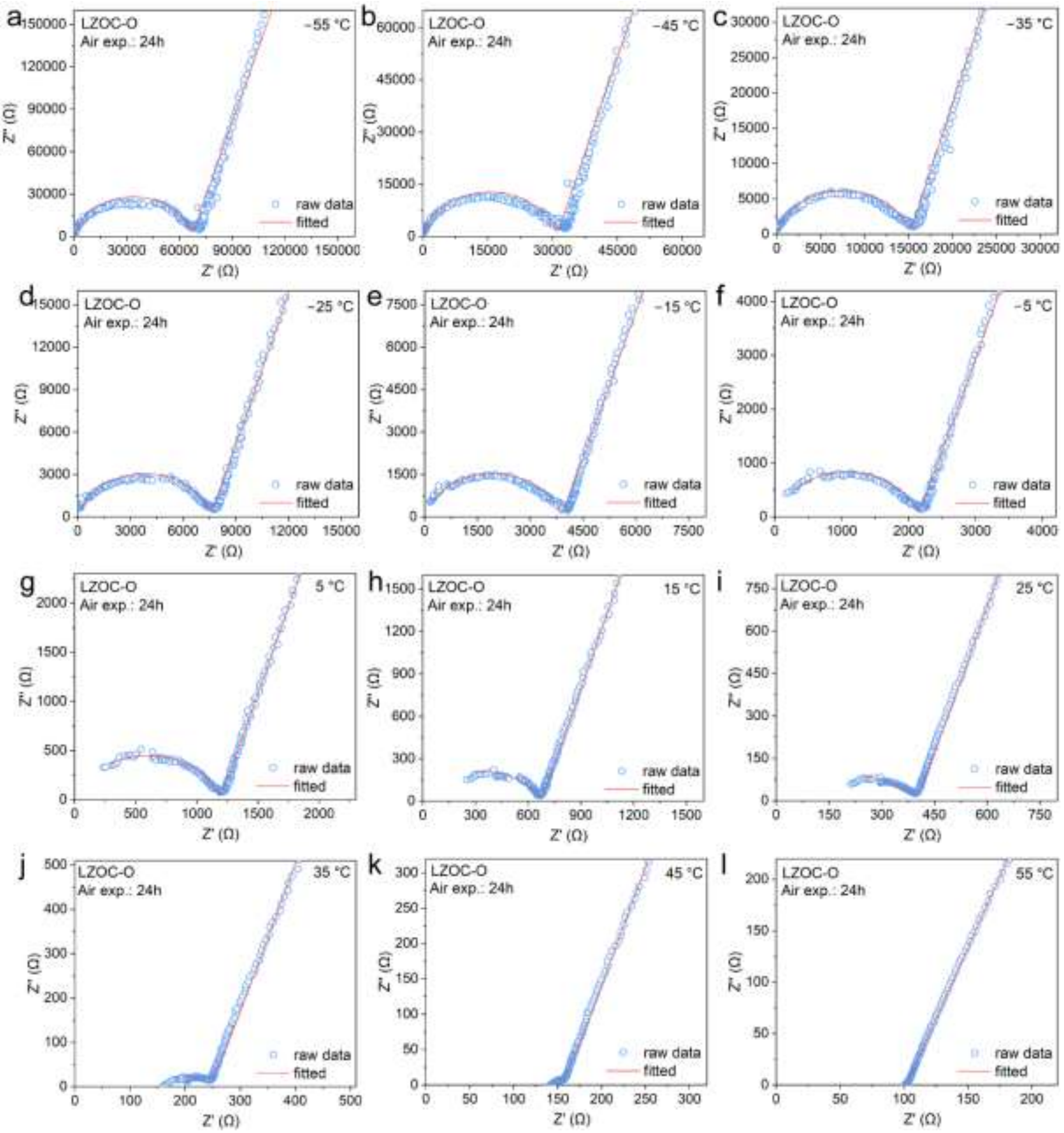

Fig. S64 Nyquist plots of LZOC-O HSSE measured immediately after conditioning in a dry room 24 h at various temperatures: (a) –55 ℃, (b) –45 ℃, (c) –35 ℃, (d) –25 ℃, (e) –15 ℃, (f) –5 ℃, (g) 5 ℃, (h) 15 ℃, (i) 25 ℃, (j) 35 ℃, (k) 45 ℃, and (l) 55 ℃.

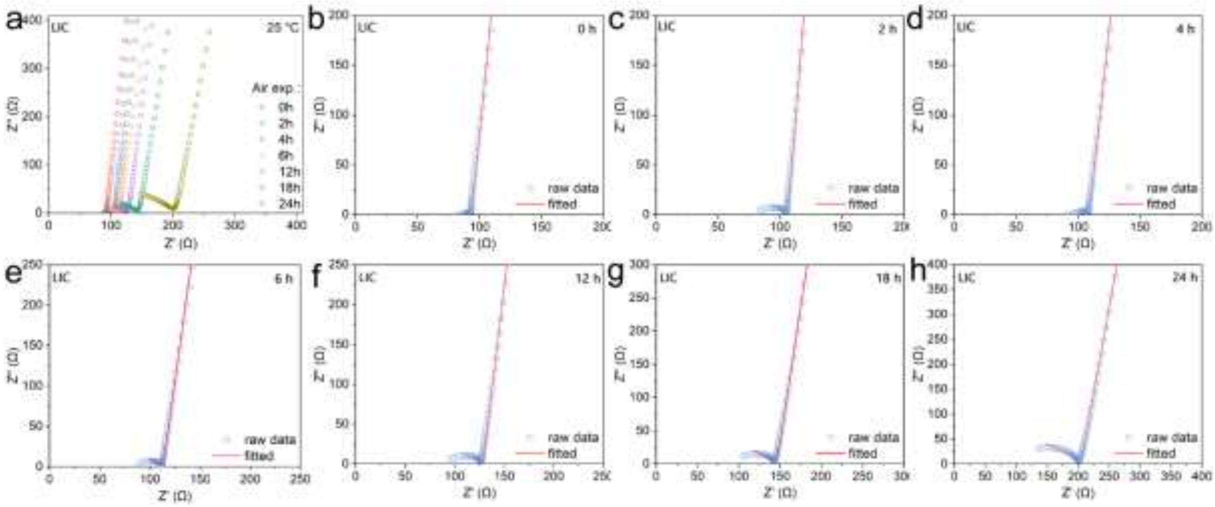

Fig. S65 Nyquist plots of Li_3InCl_6 HSSE measured immediately after conditioning in a dry room 0, 2, 4, 6, 12, 18, and 24 h at 25 ℃.

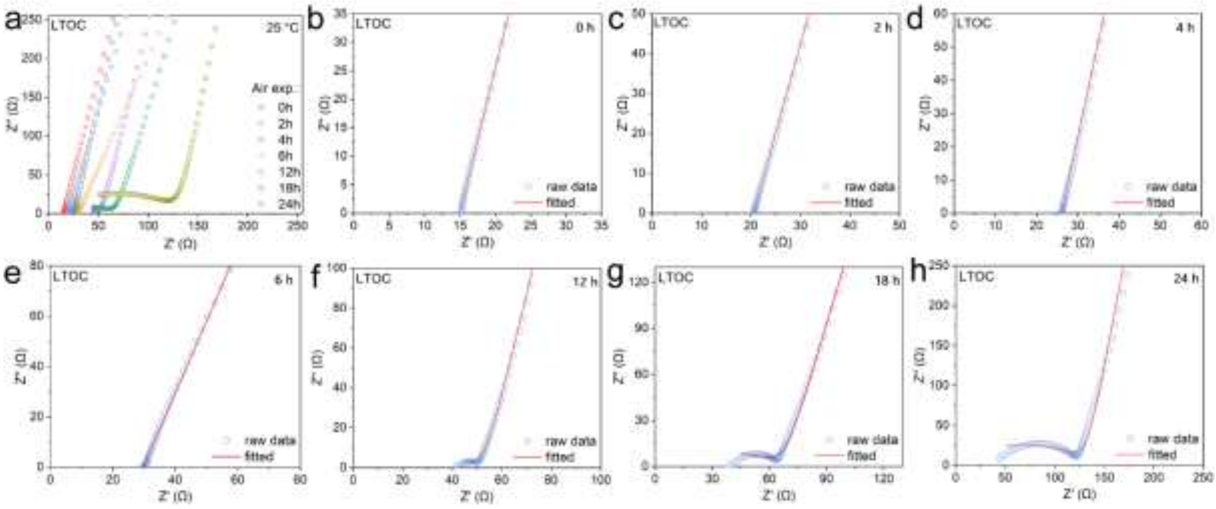

Fig. S66 Nyquist plots of $LiTaOCl_4$ HSSE measured immediately after conditioning in a dry room 0, 2, 4, 6, 12, 18, and 24 h at 25 ℃.

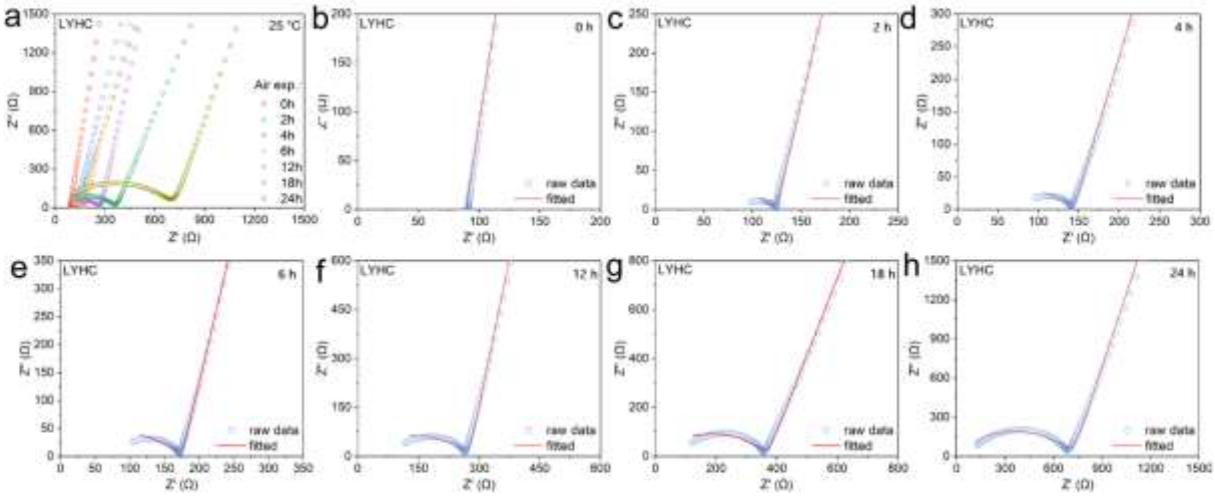

Fig. S67 Nyquist plots of LYHC HSSE measured immediately after conditioning in a dry room 0, 2, 4, 6, 12, 18, and 24 h at 25 ℃.

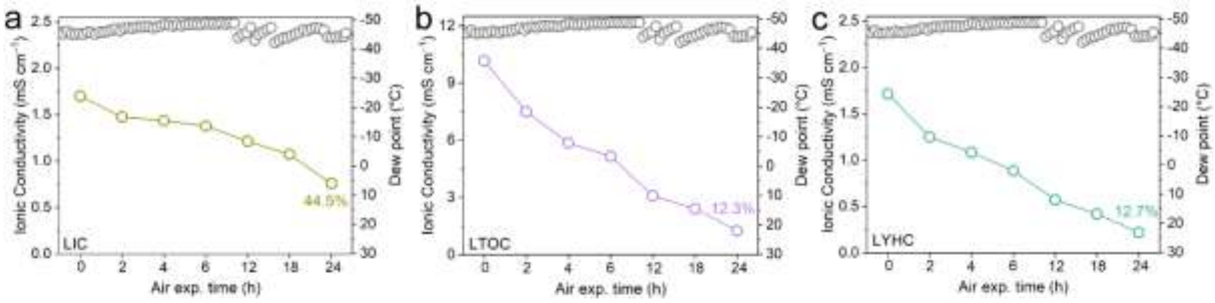

Fig. S68 Ionic conductivity degradation kinetics of LIC, LTOC, and LYHC electrolytes under prolonged air exposure.

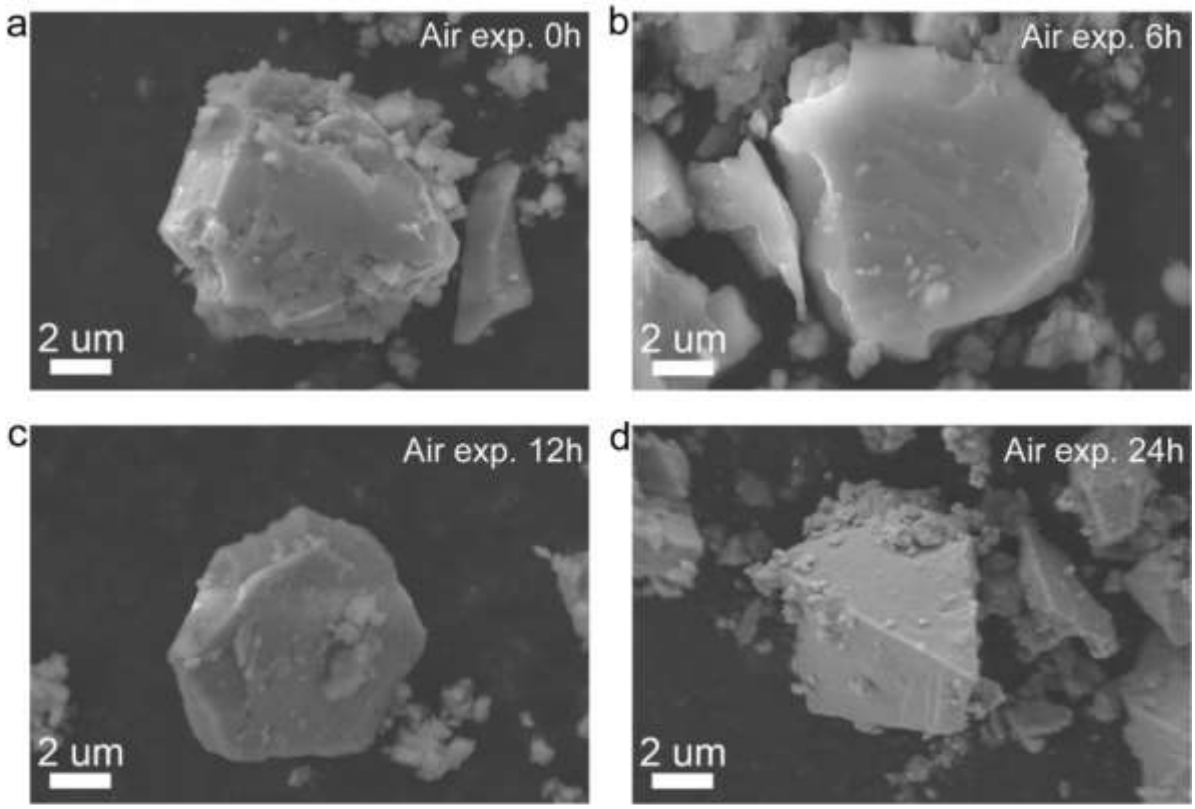

Fig. S69 SEM images of the LZOC-H HSSE at different air-exposed times. (a) 0 h, (b) 6 h, (c) 12 h, (d) 24 h.

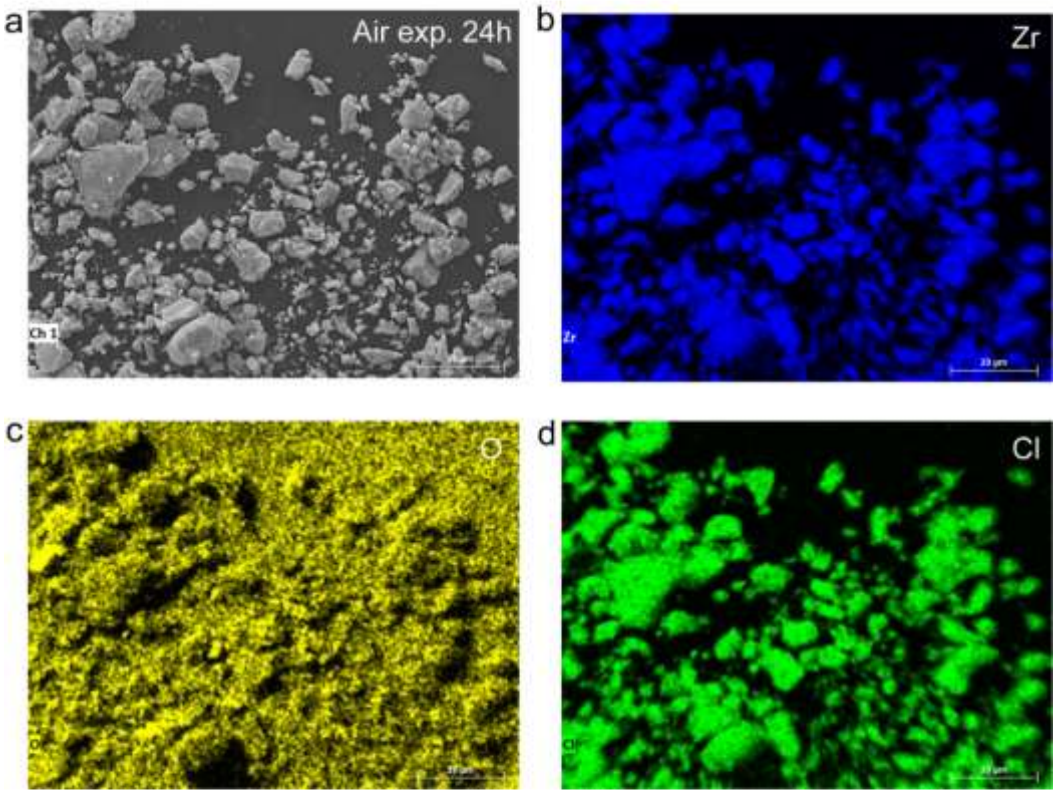

Fig. S70 SEM-EDS images of LZOC-H HSSE after air-exposed 24 h.

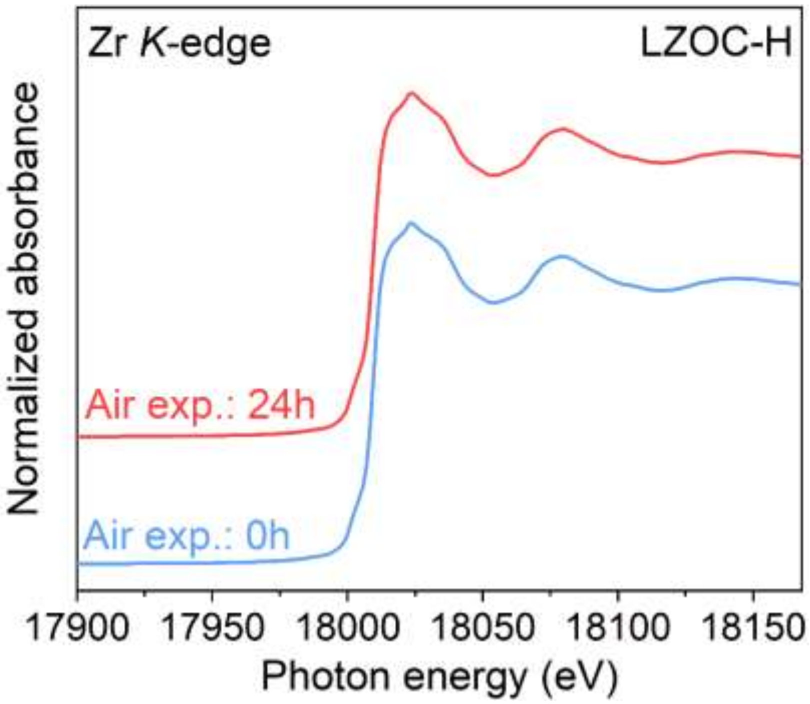

Fig. S71 Zr K-edge XANES spectra after air-exposure for 0 h and 24 h.

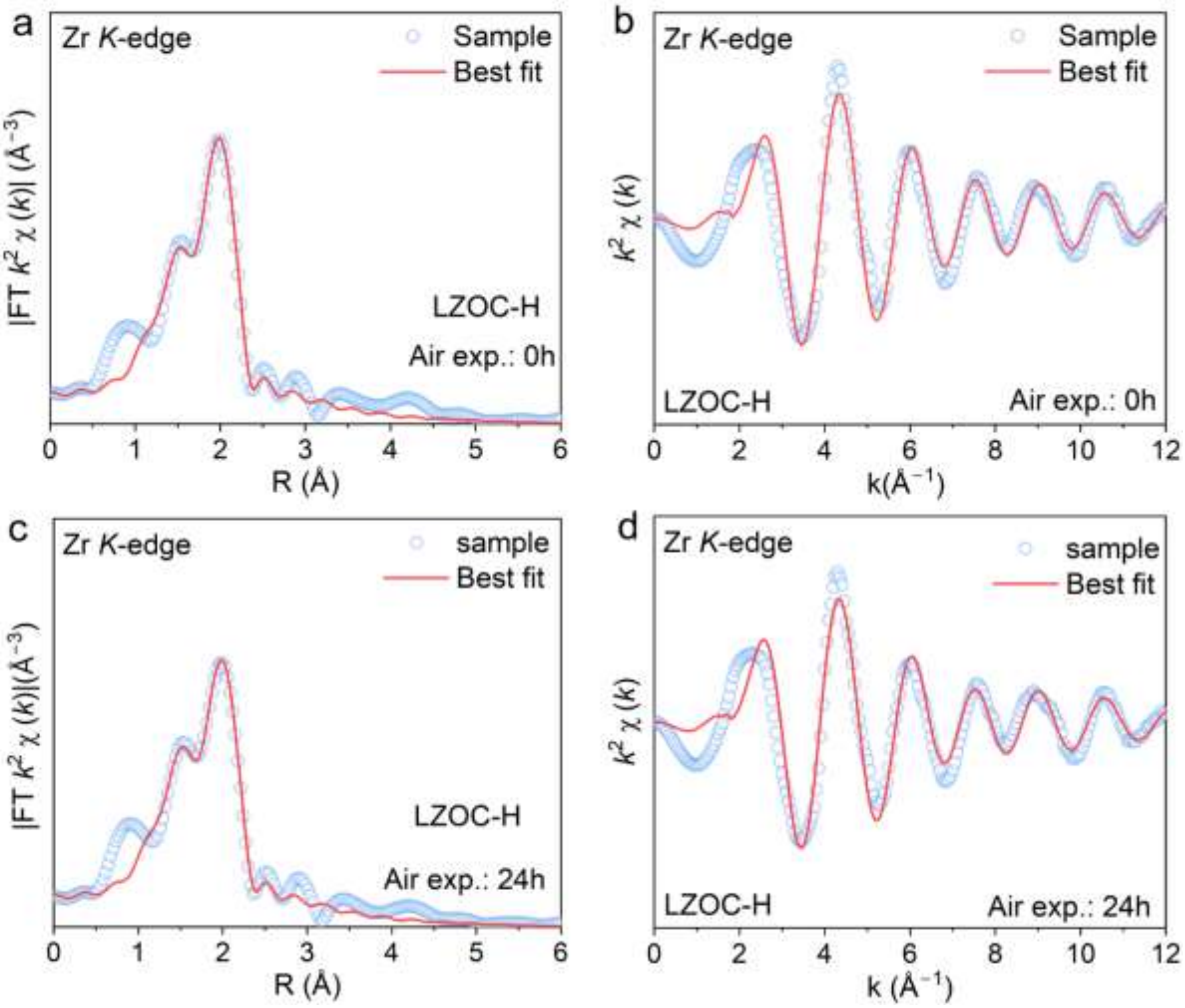

Fig. S72 Zr K-edge Fourier-transformed (FT) EXAFS fitting results of LZOC-H HSSE after air-exposure for (a,b) 0 h and (c,d) 24 h, showing the experimental data (blue circle) and Feff modeling (red line) traces.

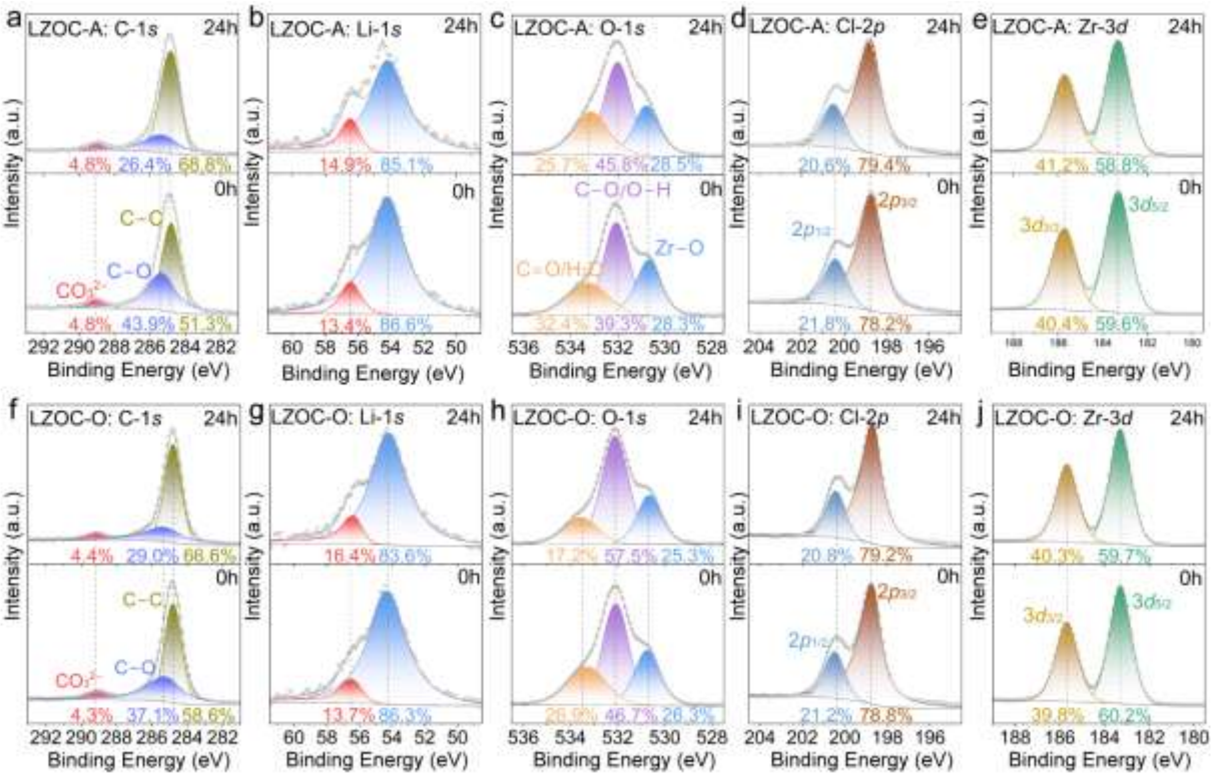

Fig. S73 (a-e) XPS spectra of LZOC-A HSSE before and after humid-air exposure for 24 h. (f-j) XPS spectra of LZOC-A before and after humid-air exposure for 24 h.

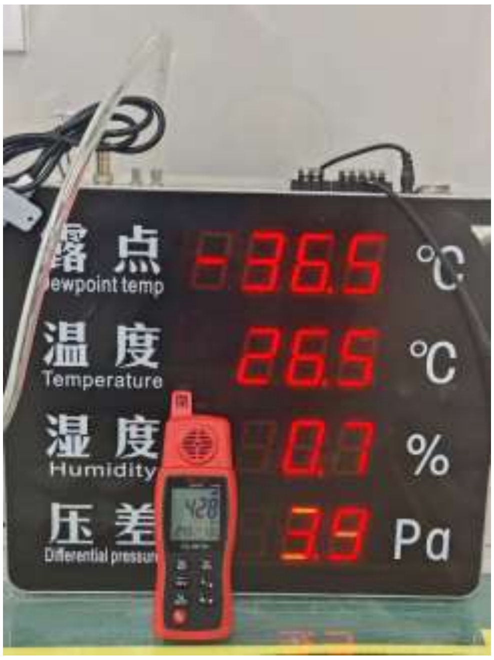

Fig. S74 The concentration of CO_2 in a dry room with a dew point of −40 ℃.

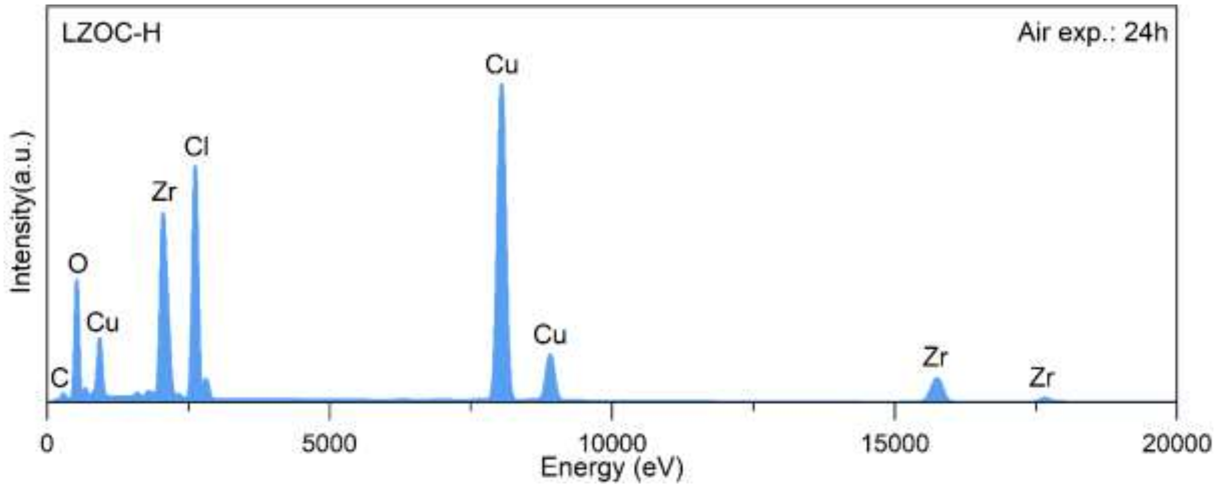

Fig. S75 Spectra from the area of TEM-EDS for the LZOC-H HSSE.

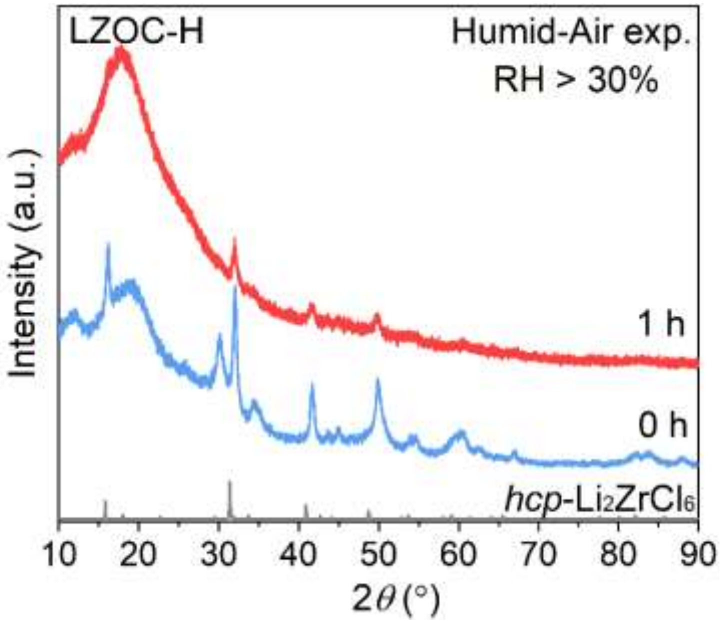

Fig. S76 XRD patterns of the LZOC-H HSSE after ambient conditions (RH > 30%) exposure for 1 h.

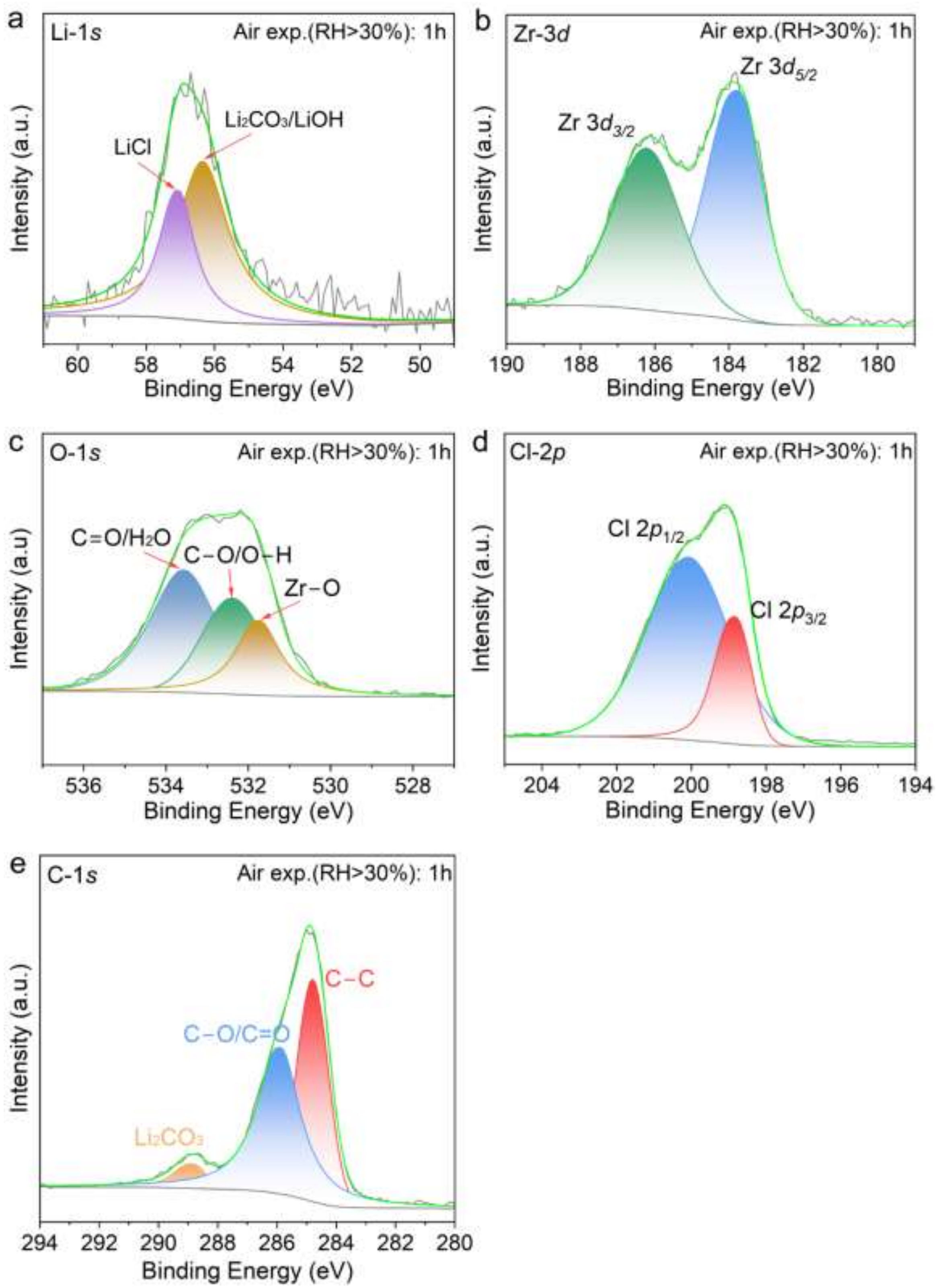

Fig. S77 XPS patterns of the LZOC-H HSSE after ambient conditions (RH > 30%) exposure for 1 h.

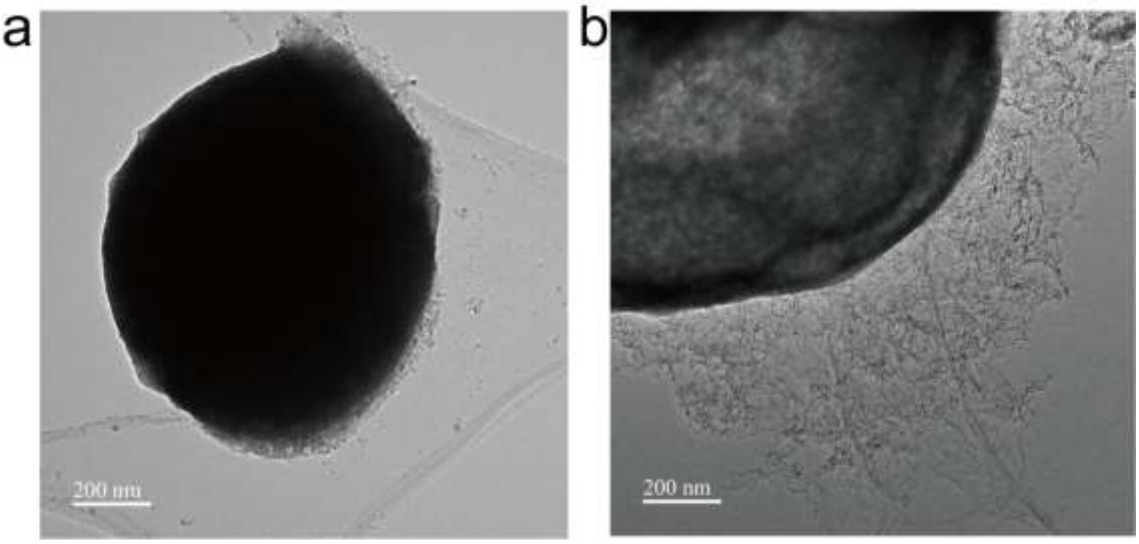

Fig. S78 HRTEM images of the LZOC-H HSSE after ambient conditions (RH > 30%) exposure for 1 h.

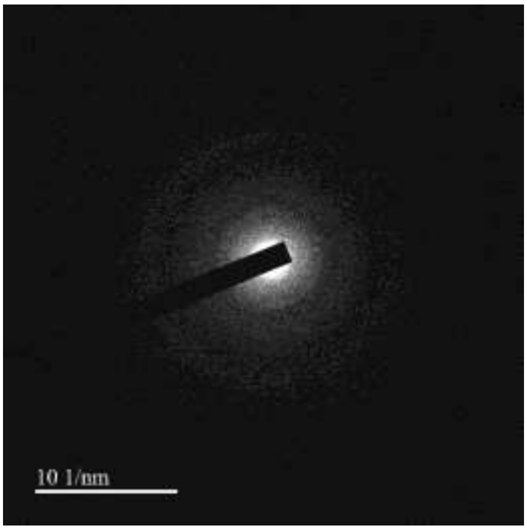

Fig. S79 SAED image of the LZOC-H HSSE after ambient conditions (RH > 30%) exposure for 1 h.

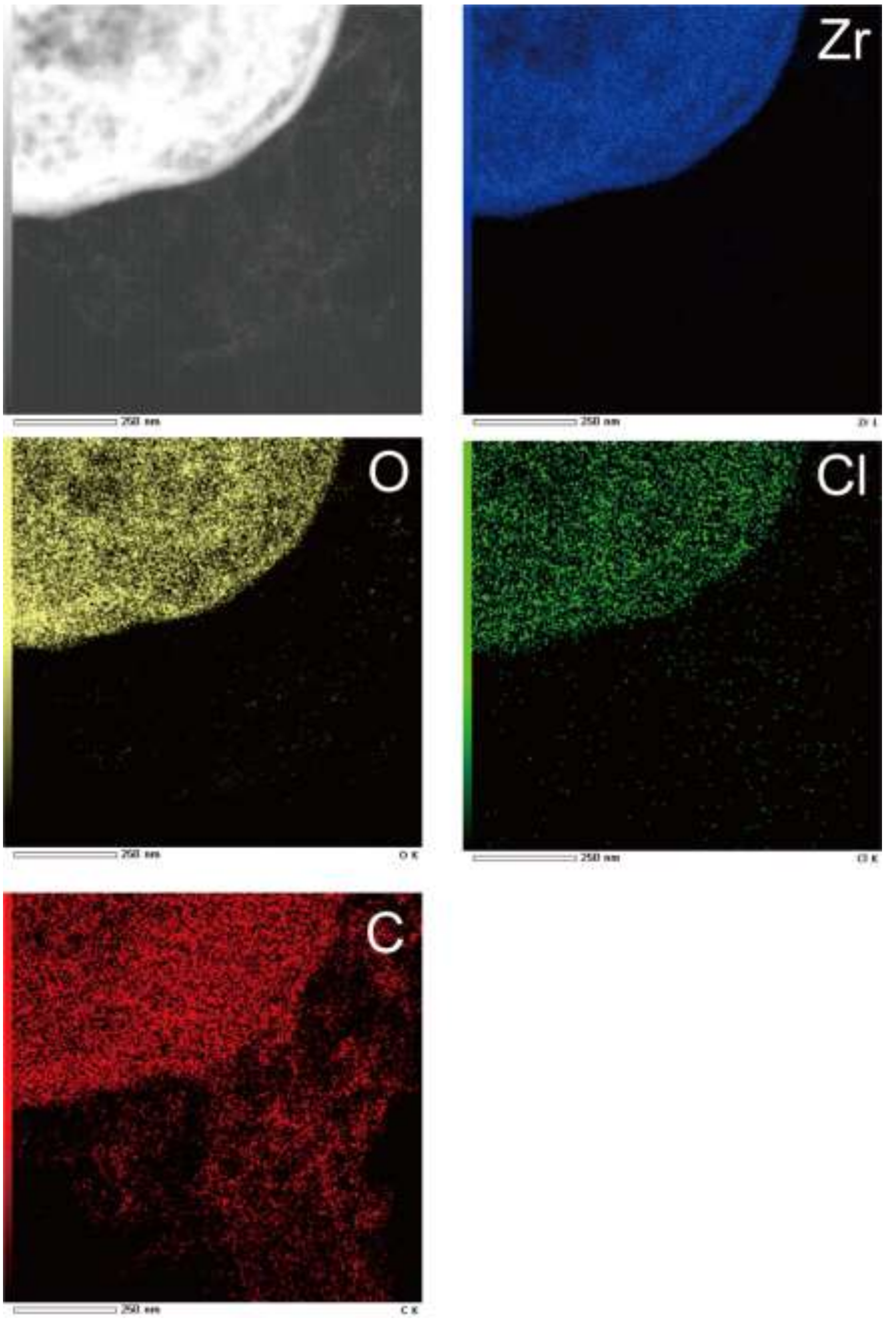

Fig. S80 TEM-EDS mapping images of the LZOC-H HSSE after ambient conditions (RH > 30%) exposure for 1 h.

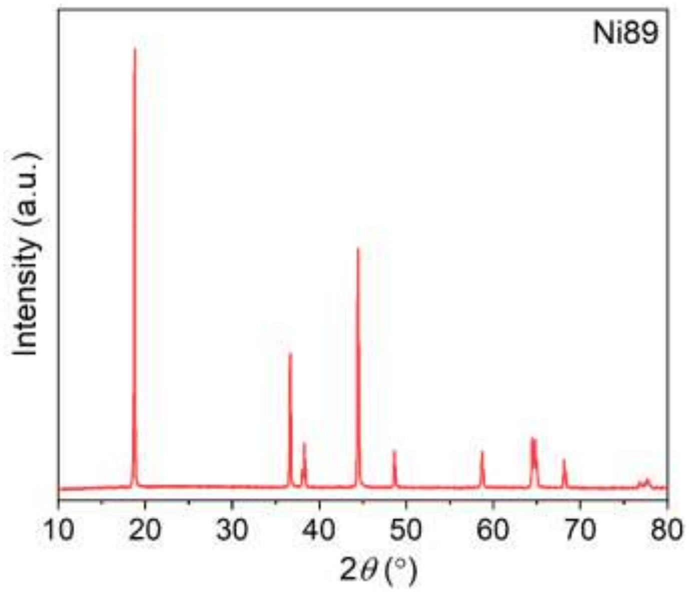

Fig. S81 XRD Pattern of the Ni89 cathode.

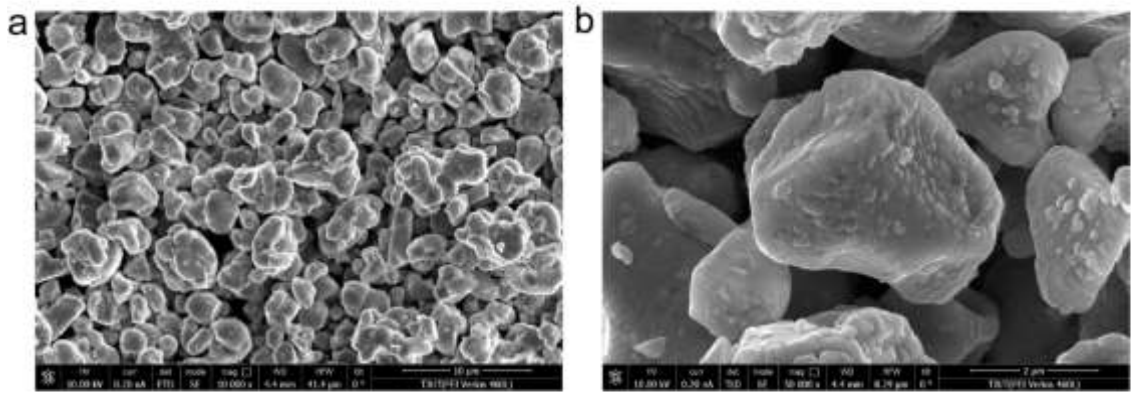

Fig. S82 SEM images of the Ni89 cathode.

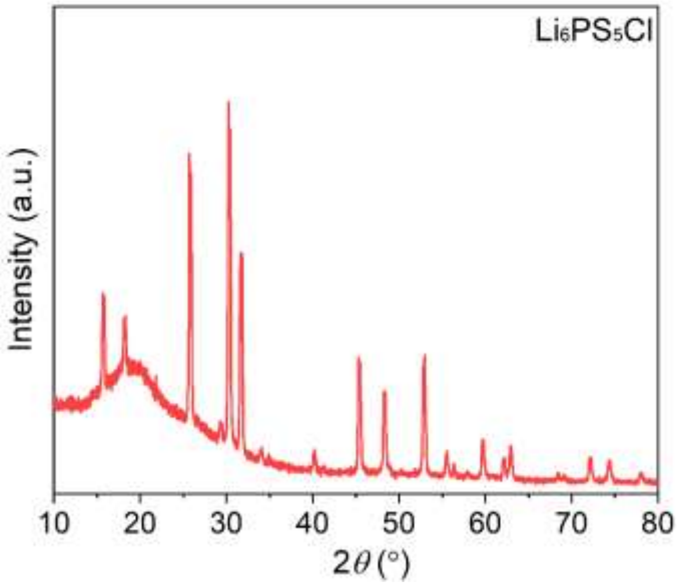

Fig. S83 XRD Pattern of the Li_6PS_5Cl electrolyte.

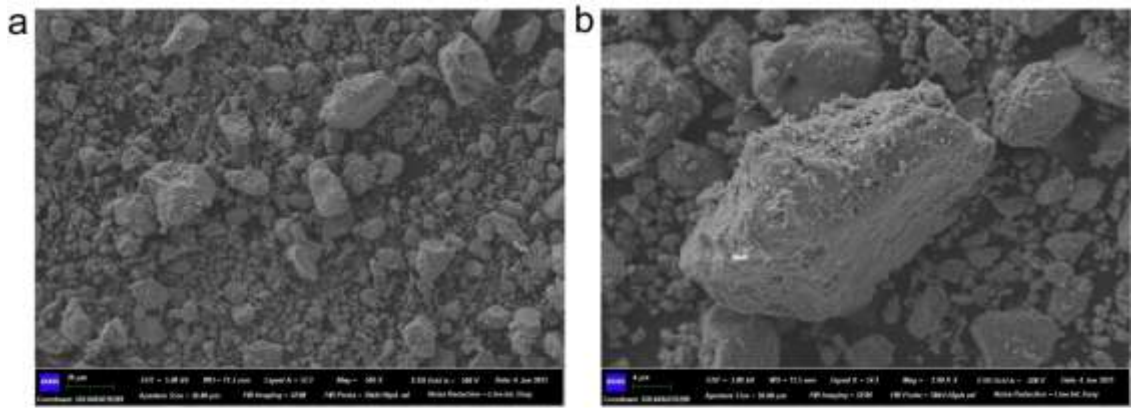

Fig. S84 SEM images of the Li_6PS_5Cl electrolyte.

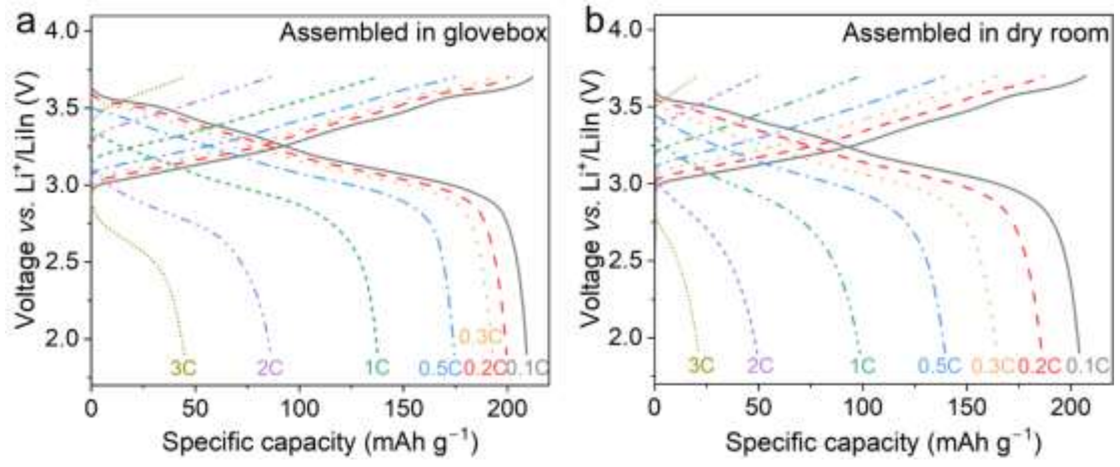

Fig. S85 Charge-discharge curves of the Ni89|LZOC-H|LPSC|LiIn mold-type ASSLBs assembled in (a) glovebox and (b) dry room at 0.1–1 C.

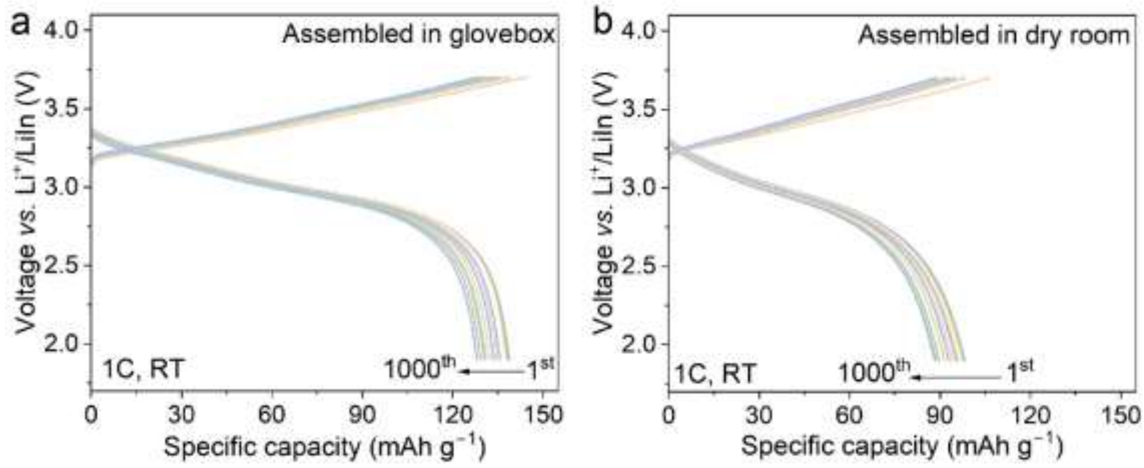

Fig. S86 Charge-discharge curves of the Ni89|LZOC-H|LPSC|LiIn mold-type ASSLBs assembled in (a) glovebox and (b) dry room at 1C.

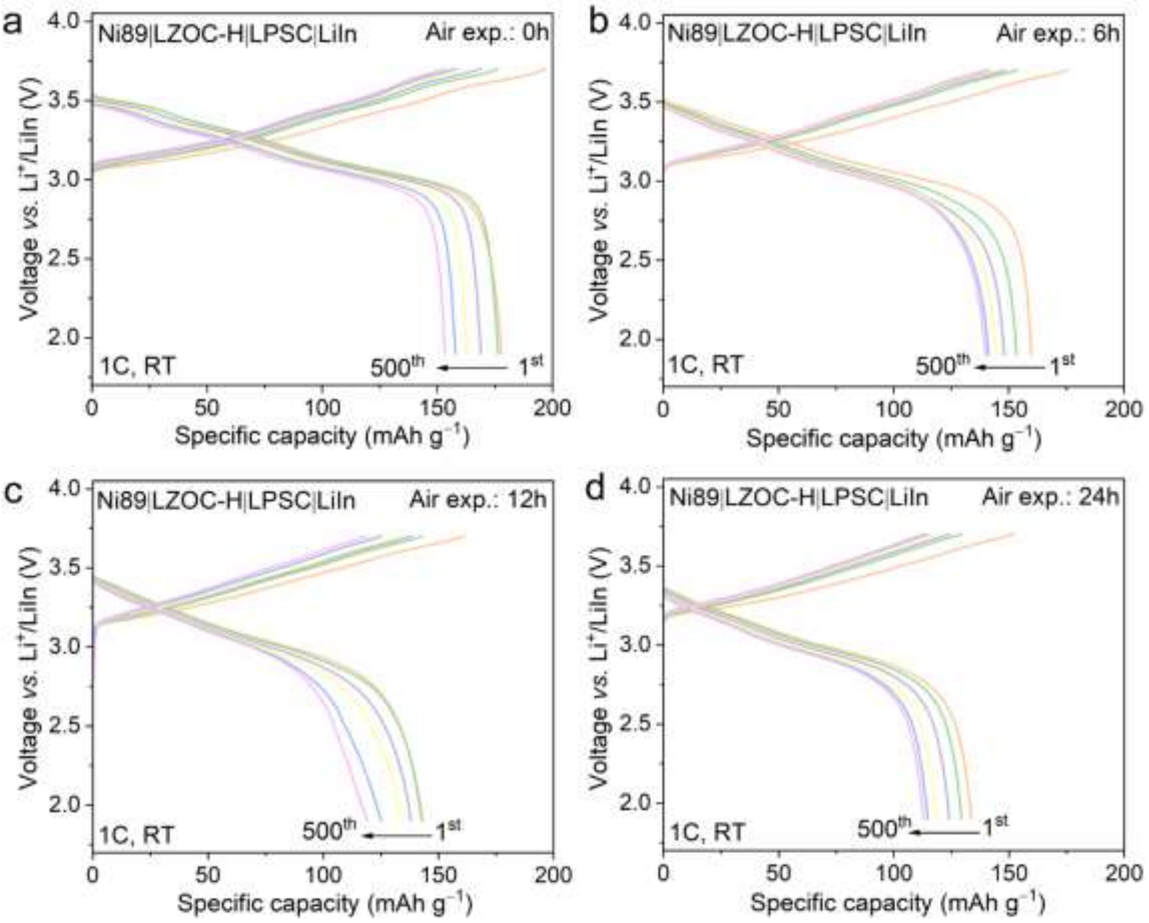

Fig. S87 Charge-discharge curves of the Ni89|LZOC-H|LPSC|LiIn mold-type ASSLBs after LZOC-H exposure in dry room for (a) 0 h, (b) 6 h, (c) 12 h, and (d) 24 h.

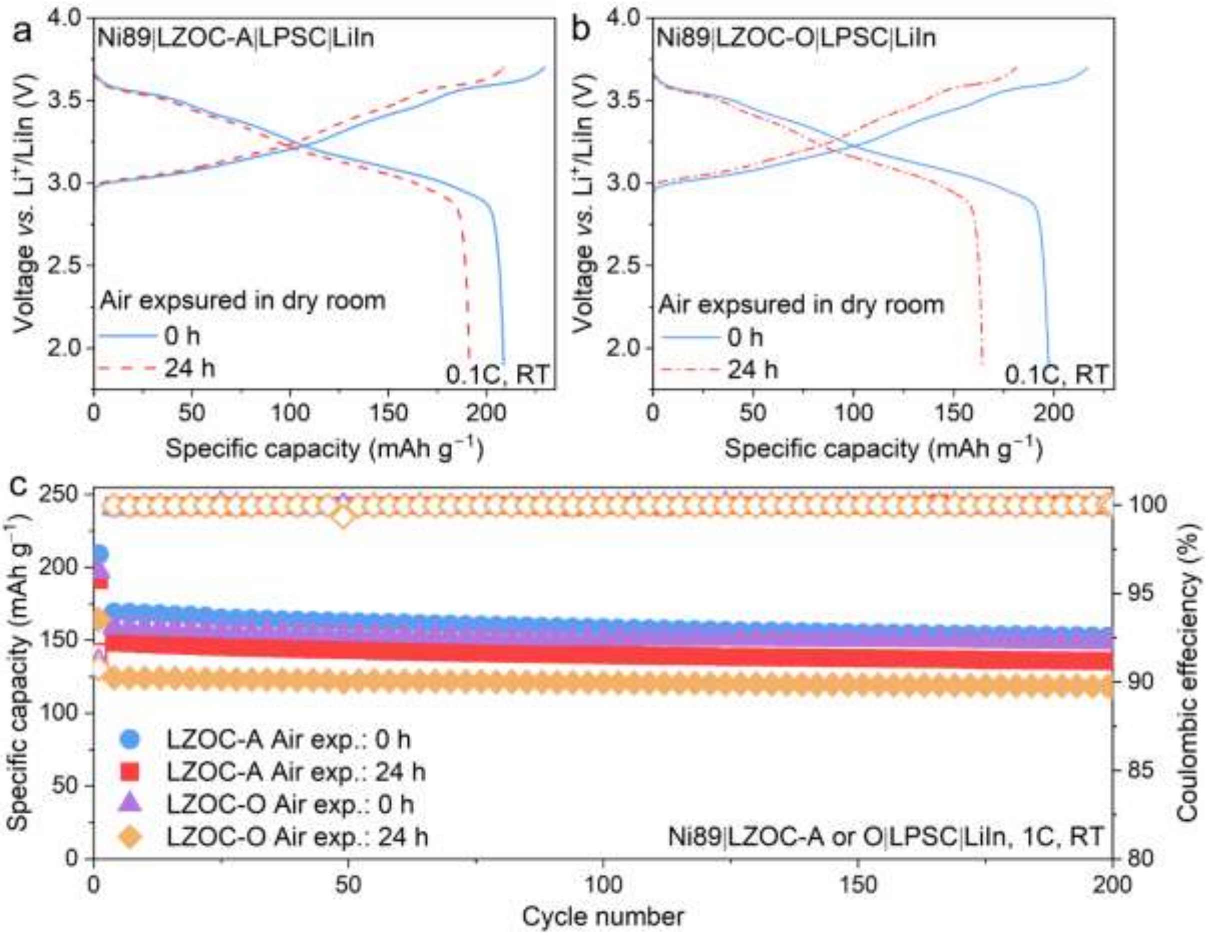

Fig. S88 Electrochemical performance of Ni89|LZOC-A or LZOC-O|LPSC|In-Li mold-type ASSLBs. (a) The charge-discharge curves of Ni89|LZOC-A|LPSC|In-Li mold-type ASSLB at 0.1C after different times of exposure in a dry room. (b) The charge-discharge curves of Ni89|LZOC-O|LPSC|In-Li mold-type ASSLB at 0.1C after different times of exposure in a dry room. (c) The long-term cycling performances of these cells assembled with LZOC-A and LZOC-O HSSEs at 1C after different times of exposure in a dry room.

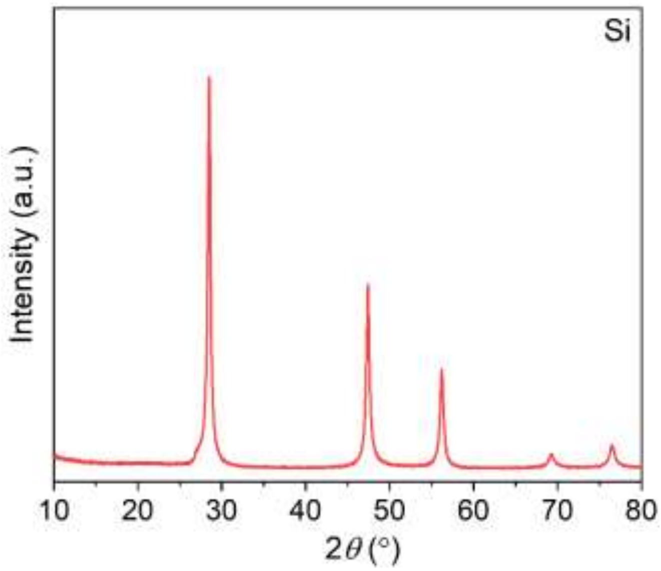

Fig. S89 XRD Pattern of the Si anode.

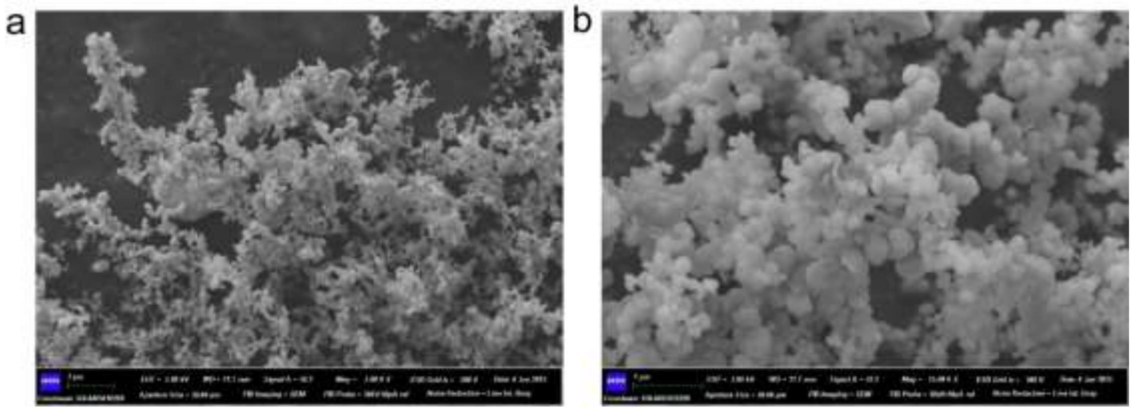

Fig. S90 SEM images of the Si anode.

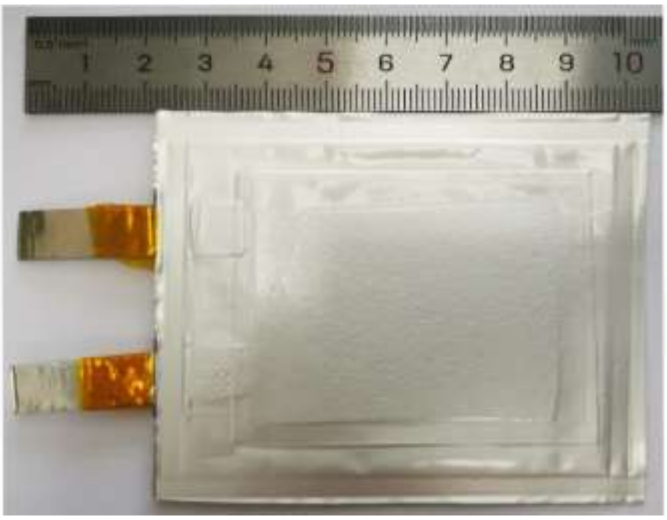

Fig. S91 Digital photo of the Ni89|LZOC-H|LPSC|In pouch-type ASSLBs.

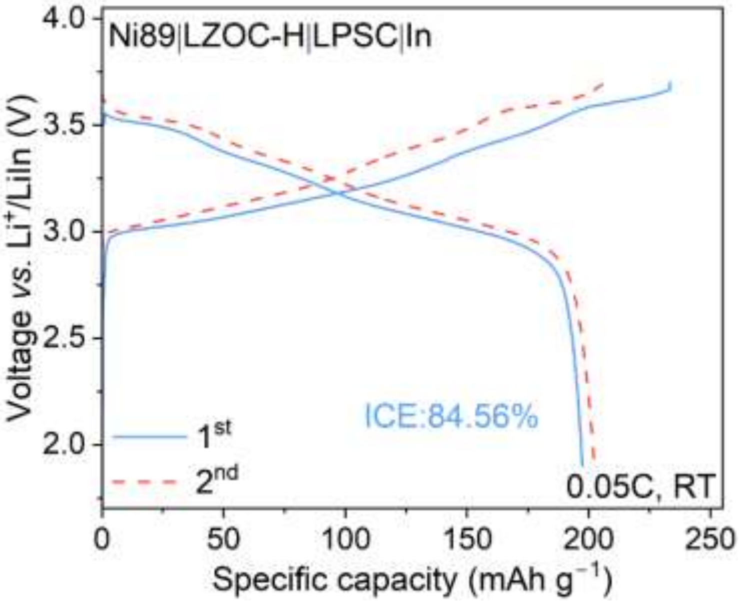

Fig. S92 Charge-discharge curves of the Ni89|LZOC-H|LPSC|In pouch-type ASSLB at 0.05C.

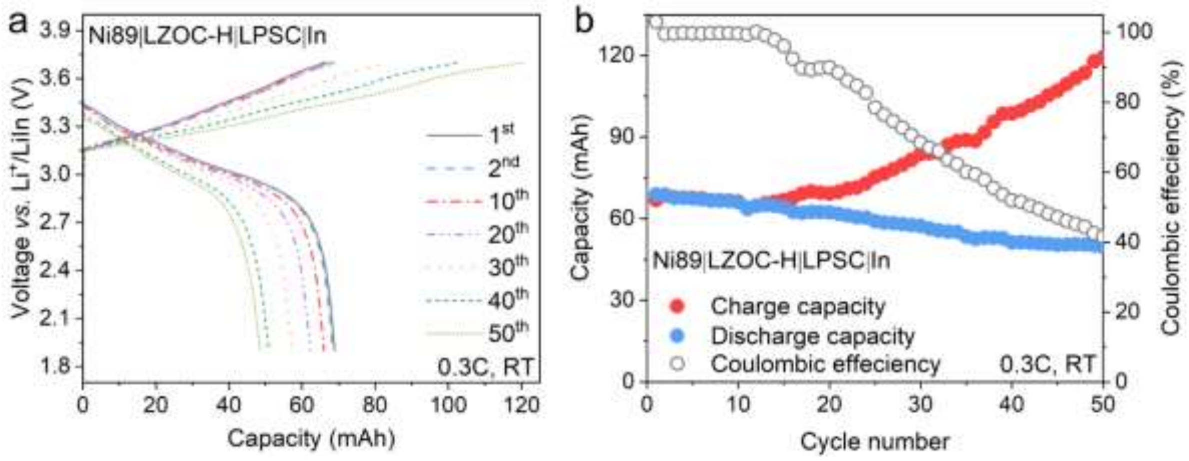

Fig. S93 (a) Charge-discharge curves and (b) Cycling performance of the Ni89|LZOC-H|LPSC|In pouch-type ASSLB at 0.3C.

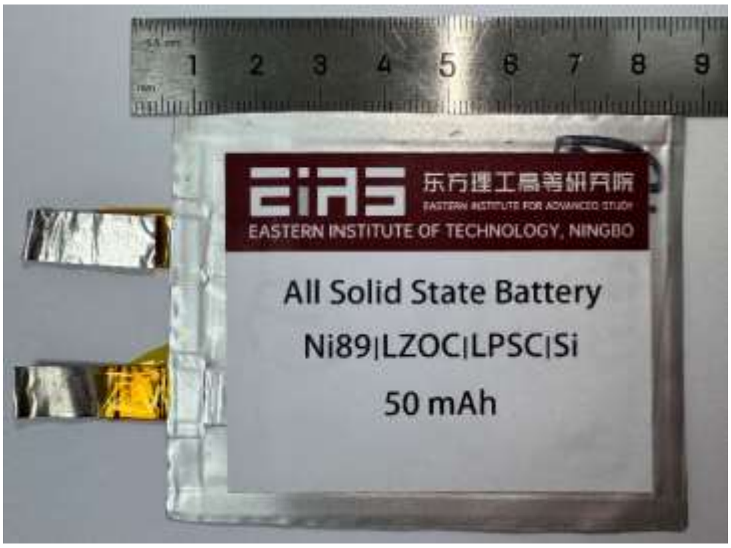

Fig. S94 Digital photo of the Ni89|LZOC-H|LPSC|Si pouch-type ASSLB.

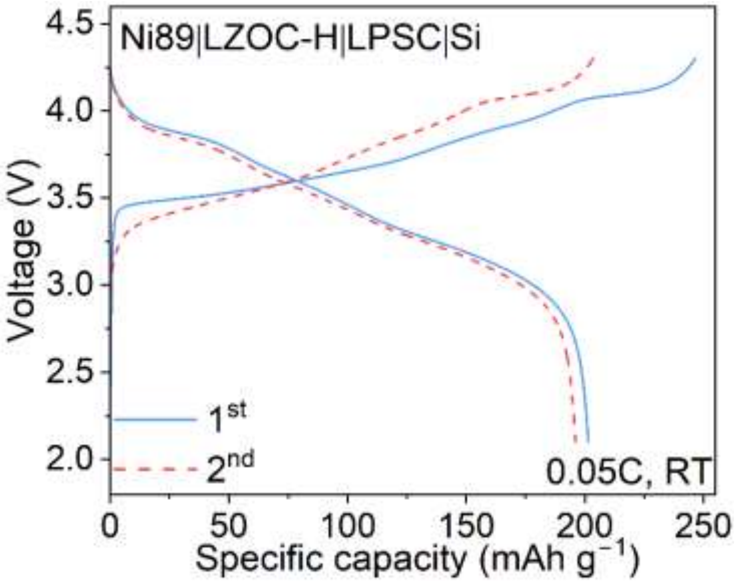

Fig. S95 Charge-discharge curves of the Ni89|LZOC-H|LPSC|Si pouch-type ASSLB at 0.05C.

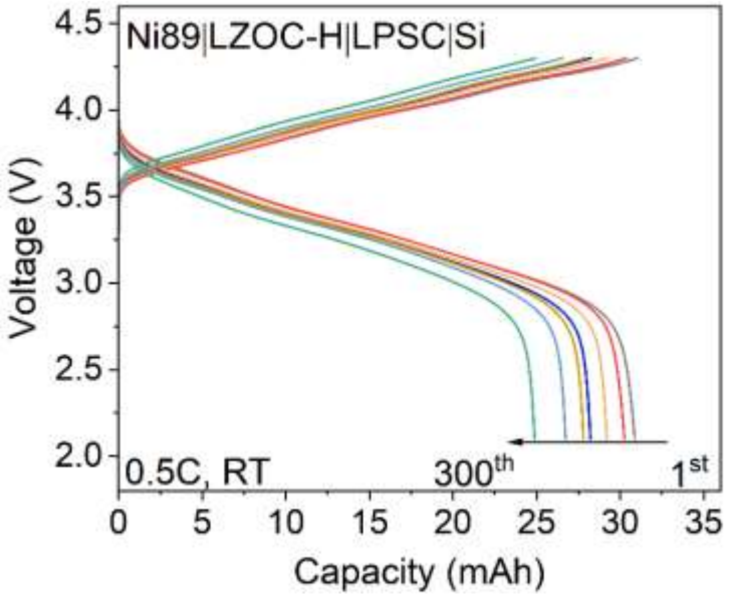

Fig. S96 Charge-discharge curves of the Ni89|LZOC-H|LPSC|Si pouch-type ASSLB at 0.5C.

Section 3. Supplementary Tables

Table S1 Comparison of the prices for raw materials of Lithium sources needed to synthesize different types of HSSEs. All the prices listed here are taken from Alfa Aesar and Sigma-Aldrich.

Chemical name	Brand	description	Purchase quantity, g	Price, $	Unit price, $/kg
LiCl	Alfa Aesar	anhydrous, 98+%	100	72.94	729.4
			500	257.65	515.3
		anhydrous, 99%	100	180.14	1801.4
			500	489.04	978.08
			2000	1783.72	891.86
			10000	8024.58	802.46
		anhydrous, 99+%	5	141.74	28348
			100	182.28	1822.8
			500	560.55	1121.1
			25000	2130.87	85.23
	Sigma-Aldrich	anhydrous, 99%	100	122.62	1226.2
			500	171.01	342.02
			1000	252.31	252.31
			25000	257.97	103.19
LiOH	Alfa Aesar	anhydrous, 98%	25	47.39	1895.6
			100	146.74	1467.4
			500	476.62	953.24
		anhydrous, 99.995%	5	124.61	24922
			25	398.40	15936
			100	1230.73	12307.3
	Sigma-Aldrich	anhydrous, 98%	100	114.46	1144.60
			500	634.94	1269.88
		anhydrous, 99.9%	100	129.54	1295.4
			500	484.59	969.18
Li_2O	Alfa Aesar	anhydrous, 99.5%	10	128.33	12833
			50	491.46	9829.2
			250	2082.05	8328.2
	Sigma-Aldrich	anhydrous, 97%	10	143.80	14380
			100	568.93	5689.3
		anhydrous, 99.9%	25	310.38	12415.2

			500	2459.06	4918.12
Li_2CO_3	Alfa Aesar	anhydrous, 98+%	250	124.61	498.44
			1000	432.65	432.65
		anhydrous, 99%	500	318.03	636.06
			2000	986.35	493.18
			10000	6515.50	651.55
		anhydrous, 99+%	100	149.31	1493.1
			500	512.59	1025.18
			2500	2111.74	844.70
	Sigma-Aldrich	anhydrous, 99%	100	118.46	1184.6
			500	184.29	368.58
			1000	275.62	275.62
		anhydrous, 99.9%	100	221.67	2216.7
			500	616.91	1233.82
		anhydrous, 99.99%	50	151.51	3030.2
			250	513.83	2055.32

Table S2 Comparison of the prices for raw materials needed to synthesize different types of HSSEs. All the prices listed here are taken from Alfa Aesar and Sigma-Aldrich.

Chemical name	Brand	description	Purchase quantity, g	Price, $	Unit price, $/kg
Lutetium (III) chloride ($LuCl_3$)	Alfa Aesar	anhydrous, 99.9%	1	295.70	259700
			5	1473.95	294790
		anhydrous, 99.98%	1	283.86	283860
			5	1210.64	242128
	Sigma-Aldrich	anhydrous, 99.99%	1	331.07	331070
			5	1102.25	220450
Scandium (III) chloride ($ScCl_3$)	Alfa Aesar	anhydrous, 99.9%	1	292.13	292130
			5	584.12	116824
	Sigma-Aldrich	anhydrous, 99.9%	1	292.13	292130
			5	584.12	116824
		anhydrous, 99.99%	1	310.55	310550
			5	1304.95	260990
Holmium (III) chloride ($HoCl_3$)	Alfa Aesar	anhydrous, 99.9%	5	196.80	39360
			25	858.57	34350
		anhydrous, 99.95%	5	333.09	66618
			25	1429.85	57194
	Sigma-Aldrich	anhydrous, 99.9%	5	315.16	63000
Erbium (III) chloride ($ErCl_3$)	Alfa Aesar	anhydrous, 99.9%	5	191.81	38362
			25	839.16	33566.4
	Sigma-Aldrich	anhydrous, 99.9%	5	462.79	92558
			25	1355.46	54218.4
Indium (III) chloride ($InCl_3$)	Alfa Aesar	anhydrous, 98+%	5	96.76	19352
			25	344.37	13774.8
		anhydrous, 99.99%	10	203.65	20365
			50	467.39	9347.8
			250	1478.37	5913.48
		hydrate, 99.99%	10	214.07	21407
			50	1070.36	21407.2
			250	2000.99	8003.96
	Sigma-Aldrich	anhydrous, 98%	10	196.92	19692
			50	684.18	13683.6
		anhydrous, 99.999%	10	428.31	42831
			50	1160.32	23206.4

		hydrate, 97%	5	131.28	26256
			50	615.83	12316.6
Yttrium (III) chloride (YCl_3)	Alfa Aesar	anhydrous, 99.9%	10	126.16	12616
			50	368.34	7366.8
			250	1032.25	4129
		anhydrous, 99.99%	10	188.81	18881
			50	596.69	11933.8
			250	1839.30	7357.2
	Sigma-Aldrich	anhydrous, 99.99%	10	261.75	26175
			50	487.28	9745.6
Ytterbium (III) chloride ($YbCl_3$)	Alfa Aesar	anhydrous, 99.99%	5	282.71	56542
			25	1436.27	57450.8
	Sigma-Aldrich	anhydrous, 99.9%	5	447.78	89556
		anhydrous, 99.99%	5	173.77	34754
			25	862.55	34502
Hafnium (IV) chloride ($HfCl_4$)	Alfa Aesar	anhydrous, 99.9%	25	173.68	6947.2
			100	693.88	6938.8
			500	3469.38	6938.76
	Sigma-Aldrich	anhydrous, 99.9%	5	494.74	98948
			25	772.75	30910
Tantalum (V) chloride ($TaCl_5$)	Alfa Aesar	anhydrous, 99.8%	5	60.65	12130
			25	206.36	8254.4
			100	490.94	4909.4
		anhydrous, 99.99%	5	89.05	17810
			25	300.70	12028
			100	817.32	8173.2
	Sigma-Aldrich	anhydrous, 99.8%	5	84.82	16964
			25	160.59	6423.6
			100	628.83	6288.3
		anhydrous, 99.99%	5	69.53	13906
			25	268.88	10755.2
Niobium (V) chloride ($NbCl_5$)	Alfa Aesar	anhydrous, 99%	10	88.91	8891
			100	514.06	5140.6
			500	2617.80	5235.6
		anhydrous, 99.9%	25	137.86	5514.4
			100	350.36	3503.6
			500	1227.06	2454.12
	Sigma-Aldrich	anhydrous, 99%	10	82.12	8212
			50	169.24	3384.8
		anhydrous, 99.9%	10	118.79	11879
			50	393.84	7876.8

Zirconium (IV) chloride ($ZrCl_4$)	Alfa Aesar	anhydrous, 98%	5	97.33	19466
			100	137.58	1375.8
			500	487.37	974.74
			2500	1791.06	716.42
		anhydrous, 99.5%	2000	1248.75	624.38
	Sigma-Aldrich	anhydrous, 99 %	5	67.84	13568
			250	179.45	717.8
			1000	212.98	212.98
		anhydrous, 99.5 %	5	39.03	7806
			100	117.03	1170.3
			500	210.41	420.82
		anhydrous, 99.9 %	10	54.73	5473
			100	342.97	3429.7
		anhydrous, 99.99 %	5	163.80	32760
			25	467.44	18697.60
Zirconium (IV) dichloride oxide octahydrate ($ZrOCl_2 \cdot 8H_2O$)	Alfa Aesar	Hydrate, 98%	100	139.72	1397.20
			500	534.18	3068.36
			2500	2154.84	861.94
		Hydrate, 98+%	25	160.27	6410.80
			100	663.48	6634.8
			500	2890.81	5781.62
	Sigma-Aldrich	Hydrate, 98%	5	90.95	18190
			100	255.92	2559.2
			500	547.97	1095.94

Table S3 Cost Evaluation of Raw Materials for Halide Solid-State Electrolytes in Large-Scale Manufacturing, using one metric ton of product as an example. All the prices listed here are taken from the minimum values from **Supplementary Tables 1 and 2**.

HSSEs	Li sources		M sources		other		Price, $/kg
	Weight percent	Price, $	Weight percent	Price, $	Weight percent	Price, $	
LZOC-H	LiCl, 19.07%	16.25	$ZrCl_4$, 72.85%	155.16	$ZrOCl_2·8H_2O$, 8.06%	69.47	240.88
Li_3InCl_6	LiCl, 35.60%	30.34	$InCl_3$, 63.49%	3754.47	--	--	3784.81
Li_3YCl_6	LiCl, 39.41%	33.59	YCl_3, 60.59%	2501.76	--	--	2535.35
Li_3ScCl_6	LiCl, 45.67%	38.92	$ScCl_3$, 54.33%	63470.48	--	--	63509.4
$LiNbOCl_4$	LiOH, 8.14%	77.59	$NbCl_5$, 91.86%	2254.35	--	--	2331.94
$LiTaOCl_4$	LiOH, 6.27%	59.77	$TaCl_5$, 93.73%	4601.58	--	--	4661.35

Table S4 Resistance values of LZOC-H at different temperatures after different ball-milling times.

Time / Temperature	2 h	4 h	6 h	8 h	10 h
	R (Ω)	R (Ω)	R (Ω)	R (Ω)	R (Ω)
55 ℃	125.3	58.6	40.1	77.9	72.5
45 ℃	200.9	86.8	58.8	113.7	105.6
35 ℃	304.6	134.1	88.7	172.2	166.9
25 ℃	474.8	215.9	136.3	277.9	270.4
15 ℃	814.5	362.3	228.7	459.9	455.5
5 ℃	1445.1	633.4	400.5	803.4	808.6
−5 ℃	2651.7	1149.8	687.4	1442.2	1487.7
−15 ℃	5182.9	2154.1	1199.9	2719.6	2857.7
−25 ℃	10348.5	4253.9	2211.1	5296.0	5711.6
−35 ℃	22192.7	9110.1	4221.9	11085.1	12109.9
−45 ℃	51104.1	20440.9	8423.8	24117.2	26602.7
−55 ℃	112146.3	49205.7	17290.3	58285.9	66246.6

Table S5 Results of crystal structure analysis by Rietveld refinement of XRD pattern for the LZOC-H sample (space group *P*-3m1).

a (Å)	*b* (Å)	*c* (Å)	*α* (°)	*β* (°)	*γ* (°)	V (Å^3)	R_{wp}%	R_p%	χ^2
10.9503	10.9503	5.9331	90	90	120	616.117	4.62	3.50	1.29

Table S6 Fractional atomic parameters by Rietveld refinements of XRD pattern for the LZOC-H sample (space group *P*-3m1).

Atom	Position	x	y	z	Occupancy factor	Uiso
Li1	6g	0.3599	0.0	0.0	0.503	0.0596
Li2	6h	0.3165	0.0	0.5	0.731	0.1208
Zr1	1a	0.0	0.0	0.0	0.725	0.0448
Zr2	2d	0.3333	0.6667	0.5315	0.518	0.0166
Zr3	1b	0.0	0.0	0.5	0.596	0.0241
Zr4	2d	0.3333	0.6667	0.9404	0.241	0.0219
Cl1	6i	0.1096	-0.1096	0.7471	0.955	0.0174
Cl2	6i	0.2287	-0.2287	0.2784	0.955	0.0348
Cl3	6i	0.4394	-0.4394	0.7699	0.955	0.0347
O1	6i	0.1096	-0.1096	0.7471	0.045	0.0174
O2	6i	0.2287	-0.2287	0.2784	0.045	0.0348
O3	6i	0.4394	-0.4394	0.7699	0.045	0.0347

Table S7 Results of crystal structure analysis by Rietveld refinement of NPD pattern for the LZOC-H sample (space group *P*-3m1).

a (Å)	*b* (Å)	*c* (Å)	*α* (°)	*β* (°)	*γ* (°)	V (Å^3)	R_{wp}%	R_p%	χ^2
10.9610	10.9610	5.9321	90	90	120	617.218	1.03	2.09	3.69

Table S8 Fractional atomic parameters by Rietveld refinements of NPD pattern for the LZOC-H sample (space group *P*-3m1).

Atom	Position	*x*	*y*	*z*	Occupancy factor	Uiso
Li1	6g	0.3599	0	0	0.503	0.0596
Li2	6h	0.3166	0	0.5	0.731	0.1208
Zr1	1a	0	0	0	0.725	0.0448
Zr2	2d	0.3333	0.6667	0.5315	0.518	0.0166
Zr3	1b	0	0	0.5	0.596	0.0241
Zr4	2d	0.3333	0.6667	0.9404	0.241	0.0219
Cl1	6i	0.1096	-0.1096	0.7471	0.955	0.0174
Cl2	6i	0.2287	-0.2287	0.2784	0.955	0.0348
Cl3	6i	0.4394	-0.4394	0.7700	0.955	0.0347
O1	6i	0.1096	-0.1096	0.7471	0.045	0.0174
O2	6i	0.2287	-0.2287	0.2784	0.045	0.0348
O3	6i	0.4394	-0.4394	0.7700	0.045	0.0347

Table S9 Structural parameters of LZOC-H HSSE extracted from Zr *K*-edge EXAFS fitting.

sample	shell	Bond length (Å)	Coordination number	σ^2 (Å^2)
LZOC-H	Zr-O	2.07±0.04	1.8±0.5	0.004±0.002
	Zr-Cl	2.48±0.01	5.9±1.7	0.009±0.003

The value of the amplitude reduction factor (S_0^2) lines between 0.7 and 1. Bond length is the interactomic distance. CN is the coordination number. σ^2 is Debye-Waller factor (a measure of thermal and static disorder in absorber scatter distance). ΔE_0 shift is edge-energy shift (the difference between the zero kinetic energy value of the sample and that of the theoretical model), and the value is -1.6±1.1. R factor is used to value the goodness of the fitting, and the value is 0.012.

Table S10 Resistance values of LZOC-H at different temperatures after different air exposure times in the dry room (DP < −40 ℃).

Time / Temperature	0 h	2 h	4 h	6 h	12 h	18 h	24 h
	R (Ω)	R (Ω)	R (Ω)	R (Ω)	R (Ω)	R (Ω)	R (Ω)
55 ℃	42.0	51.5	41.9	41.4	48.2	43.6	54.6
45 ℃	62.7	77.8	62.9	61.9	73.3	66.1	65.7
35 ℃	98.1	121.1	96.9	95.8	111.9	102.9	126.9
25 ℃	123.6	141.9	154.6	153.1	161.8	178.1	207.2
15 ℃	268.6	342.1	261.1	263.3	309.1	287.2	360.8
5 ℃	478.8	638.4	467.4	472.0	555.3	510.9	655.5
−5 ℃	847.1	1128.8	822.2	829.2	978.6	907.7	1155.1
−15 ℃	1549.8	2054.5	1478.1	1501.4	1774.7	1656.7	2099.1
−25 ℃	2983.8	3909.3	2793.8	2850.7	3392.8	3174.6	4055.4
−35 ℃	5958.4	7820.6	5574.7	5652.1	6783.1	6301.8	8201.3
−45 ℃	12322.5	16051.7	11430.2	11661.7	13981.2	13221.9	17216.7
−55 ℃	26235.6	34301.4	24349.8	24818.7	30120.3	28330.7	36926.2

Table S11 Resistance values of LZOC-A at different temperatures after different air exposure times in the dry room (DP < −40 ℃).

Time / Temperature	0 h	2 h	4 h	6 h	12 h	18 h	24 h
	R (Ω)	R (Ω)	R (Ω)	R (Ω)	R (Ω)	R (Ω)	R (Ω)
55 ℃	39.5	47.4	52.1	51.6	59.3	65.4	74.2
45 ℃	59.4	69.6	79.6	78.4	90.3	98.2	111.7
35 ℃	91.6	104.8	124.4	120.8	141.4	154.4	178.3
25 ℃	145.6	163.6	203.1	198.3	231.9	258.5	295.3
15 ℃	246.1	280.4	345.2	342.6	400.2	446.9	515.4
5 ℃	439.8	498.4	622.3	612.7	713.2	796.6	930.6
−5 ℃	764.9	867.3	1094.7	1083.5	1263.6	1415.5	1670.2
−15 ℃	1383.4	1544.7	2015.3	1988.6	2358.1	2650.6	3112.9
−25 ℃	2612.4	2899.7	3876.3	3865.5	4599.6	5130.4	6168.9
−35 ℃	5167.7	5683.7	7826.7	7850.3	9302.2	10473.2	12520.2
−45 ℃	10630.6	11729.2	16162.5	16167.4	19461.6	21726.0	26519.6
−55 ℃	22513.6	24731.7	34784.4	35318.6	42447.1	47512.6	58494.6

Table S12 Resistance values of LZOC-O at different temperatures after different air exposure times in the dry room (DP < −40 ℃).

Temperature \ Time	0 h	2 h	4 h	6 h	12 h	18 h	24 h
	R (Ω)	R (Ω)	R (Ω)	R (Ω)	R (Ω)	R (Ω)	R (Ω)
55 ℃	27.9	31.6	39.5	43.0	57.0	75.3	101.8
45 ℃	38.9	44.5	56.2	62.3	83.1	113.8	151.2
35 ℃	54.7	65.6	82.1	91.2	125.8	173.1	238.7
25 ℃	79.6	97.1	123.6	134.6	191.3	279.7	392.4
15 ℃	123.0	148.9	190.7	215.3	318.8	463.6	668.4
5 ℃	200.3	241.9	317.7	359.2	552.9	801.6	1191.4
−5 ℃	320.8	403.1	534.7	614.6	937.2	1414.6	2143.7
−15 ℃	536.3	683.2	914.9	1038.1	1667.6	2516.7	3902.3
−25 ℃	924.2	1203.5	1623.7	1846.1	3057.7	4721.6	7596.8
−35 ℃	1654.1	2180.4	2980.4	3412.8	5847.3	9287.5	15150.3
−45 ℃	3097.0	4118.2	5691.7	6468.4	11591.6	18510.8	31097.2
−55 ℃	6067.6	8057.8	11237.7	12798.9	23524.3	38654.0	67045.4

Table S13 Ionic conductivities of LZOC-H, LZOC-A, and LZOC-O at 25 ℃ after different air exposure times in the dry room (DP < −40 ℃).

Electrolytes / σ_{Li} (mS cm^{-1})	LZOC-H	LZOC-A	LZOC-O
0 h	1.23	1.06	1.89
2h	1.09	0.94	1.58
4 h	1.01	0.76	1.24
6 h	0.99	0.77	1.13
12 h	0.93	0.66	0.79
18 h	0.86	0.59	0.55
24 h	0.76	0.52	0.39

Table S14 Comparison with other reported studies on the air stability of halide solid-state electrolytes.

HSSEs	σ_{Li} (mS cm^{-1})	strategy	treatment condition	results	ref.
Li_3InCl_6	1.49, 25 ℃	water-mediated synthesis	exposed to ambient air (RH~30%) for 12 h	σ_{Li} retention was 90.6% after reheating in vacuum	[5]
Li_2ZrCl_6	0.81, 25 ℃	as-milled	exposed to dry air (RH~5%) for 24 h	σ_{Li} retention was 93.5%	[6]
$Li_3InCl_6@Al_2O_3$	--	ALD Al_2O_3 coating	exposed to ambient air (35±5% RH)	the water absorption rate in air reduces to 1/4 of initial and liquefaction time in air increases by 7 times	[7]
$Li_{2.8}Zr_{0.2}In_{0.8}Cl_6$	0.77, 30 ℃	In^{3+} doping	exposed to ambient air for 25 min	can be restored to initial structure after postheating	[8]
$Li_{2.8}Zr_{0.2}In_{0.8}Cl_6$	1.4, 25 ℃	In^{3+} doping	exposed to dry air (RH~5%) for 24 h	σ_{Li} retention was 82.5%	[9]
$Li_{2.4}Zr_{0.8}Zn_{0.2}Cl_6$	1.13, 30 ℃	Zn^{2+} doping	exposed to dry air (RH~10%) for 2 h	σ_{Li} retention was 86.7%	[10]
$Li_2ZrCl_6@LiF\text{-}ZrF_4$	0.21, 30 ℃	$LiF\text{-}ZrF_4$ coating	exposed to dry air (RH~25%) for 2 h	σ_{Li} retention was 68.4%	[11]
$Li_3GaF_{5.3}Cl_{0.7}$	0.1, 25 ℃	liquid metal Ga catalysis	exposed to ambient air (RH~35%) for 24 h	without conductivity degradation	[12]
$Li_2ZrCl_4{\cdot}CN_2$	1.4, 25 ℃	NCN^{2-} substituted	exposed to dry air (RH~10%) for 24 h	σ_{Li} retention was 71.4%	[13]
$Li_3InCl_{5.8}F_{0.2}$	1.17, 25 ℃	F^- doping	exposed to ambient air (RH~20%) for 0.5 h	σ_{Li} retention was 42.0%	[14]
Li_2TiF_6	0.097, 25 ℃	hydrothermal method	exposed to ambient air (RH~70%) for 0.5 h	σ_{Li} no significant change	[15]
$Li_3YCl_6@g\text{-}C_3N_4$	0.33, 25 ℃	$g\text{-}C_3N_4$ coating	exposed to ambient air (RH~20%) for 5 min	the water absorption rate has decreased significantly.	[16]

Table S15 Structural parameters of LZOC-H HSSE after 24-hour air-exposed extracted from Zr *K*-edge EXAFS fitting.

sample	shell	Bond length (Å)	Coordination number	σ^2 (Å^2)
LZOC-H-24 h	Zr-O	2.07±0.04	1.9±0.5	0.003±0.002
	Zr-Cl	2.48±0.01	5.9±1.7	0.009±0.004

The value of the amplitude reduction factor (S_0^2) lines between 0.7 and 1. Bond length is the interactomic distance. CN is the coordination number. σ^2 is Debye-Waller factor (a measure of thermal and static disorder in absorber scatter distance). ΔE_0 shift is edge-energy shift (the difference between the zero kinetic energy value of the sample and that of the theoretical model), and the value is -2.0±1.2. R factor is used to value the goodness of the fitting, and the value is 0.014.

Table S16 TEM-EDS mapping result of the LZOC-H HSSE after ambient conditions (RH > 30%) exposure for 1 h.

Element	Counts	Mass%
Zr	197237.05	83.45
O	52254.28	9.08
Cl	519.30	0.06
C	19000.20	7.41

Table S17 The performance parameters of the Ni89|LZOC-H|LPSC|In and Ni89|LZOC-H|LPSC|Si pouch cells.

Pouch cell	Ni89|LZOC-H|LPSC|In	Ni89|LZOC-H|LPSC|Si
Cathode material	Ni89	Ni89
Anode material	In	Si
Cathode current collector	Al foil	Al foil
Anode material collector	Cu foil	Cu foil
Active material content	70%	70%
Cathode loading (mg cm^{-2})	26.4	13.0
Cathode area capacity (mA cm^{-2})	5.28	2.60
Anode area capacity (mA cm^{-2})	37.0	4.0
Cathode area (cm × cm)	3.5 × 5	3.5 × 5
Anode area (cm × cm)	4.0 × 5.5	4.0 × 5.5
Electrolyte area (cm × cm)	5.5 × 6.0	5.5 × 6.0

Section 4. Supplementary References

1. Larson AC, Von Dreele RB. Gsas. *Report lAUR*. 1994: 86–748.
2. Toby BH, Von Dreele RB. GSAS-II: the genesis of a modern open-source all purpose crystallography software package. *Applied Crystallography*. 2013; **46**: 544–549.
3. Rietveld HM. A profile refinement method for nuclear and magnetic structures. *Applied Crystallography*. 1969; **2**: 65–71.
4. Momma K, Izumi F. VESTA 3 for three-dimensional visualization of crystal, volumetric and morphology data. *Applied Crystallography*. 2011; **44**: 1272–1276.
5. Li X, Liang J, Luo J *et al.* Air-stable Li_3InCl_6 electrolyte with high voltage compatibility for all-solid-state batteries. *Energy Environ Sci*. 2019; **12**: 2665–2671.
6. Wang K, Ren Q, Gu Z *et al.* A cost-effective and humidity-tolerant chloride solid electrolyte for lithium batteries. *Nat Commun*. 2021; **12**: 4410.
7. Wang S, Xu X, Cui C *et al.* Air sensitivity and degradation evolution of halide solid state electrolytes upon exposure. *Adv Funct Mater*. 2022; **32**: 2108805.
8. Luo X, Hu X, Zhong Y *et al.* Degradation Evolution for Li_2ZrCl_6 Electrolytes in Humid Air and Enhanced Air Stability via Effective Indium Substitution. *Small*. 2024; **20**: 2306736.
9. Wang K, Gu Z, Liu H *et al.* High-Humidity-Tolerant Chloride Solid-State Electrolyte for All-Solid-State Lithium Batteries. *Adv Sci*. 2024; **11**: 2305394.
10. Lei P, Wu G, Liu H *et al.* Boosting Ion Conduction and Moisture Stability Through Zn^{2+} Substitution of Chloride Electrolytes for All-Solid-State Lithium Batteries. *Adv Energy Mater*. 2025; **15**: 2405760.
11. Liu H, Li Y, Dong C *et al.* Fluorinated coating stabilizing halide solid electrolytes for all-solid-state lithium metal batteries. *Energy Storage Mater*. 2025; **75**: 104107.
12. Nie X, Hu J, Lei M *et al.* Humid‐Air Stable and High‐conductivity Fluoride Solid Electrolytes Induced by Liquid Metal Activation and Ga_2O_3 in situ Catalysis. *Adv Energy Mater*. 2025; **15**: 2402997.
13. Zong J, Li J, Cao Y *et al.* Boosting lithium compatibility and air stability of halide electrolytes through cyanamide substitution. *Chem Commun*. 2025; **61**: 9290–9293.
14. Wang Q, Ma X, Liu Q *et al.* Fluorine-doped Li3InCl6 to enhance ionic conductivity and air stability. *J Alloys Compd*. 2023; **969**: 172479.
15. Nie X, Lei M, Hu J *et al.* Cost-Effective and Humid Air-Stable Fluoride Solid Electrolyte with High Ionic Conductivity Induced by Microstructural Modulation. *ACS Mater Lett*. 2025; **7**: 1768–1776.
16. Long Z, Sun J, Zhang Y *et al.* Enhancing the moisture stability of halide solid-state electrolytes via graphitic carbon nitride coating: a case study on Li 3 YCl 6. *Chem Commun*. 2025; **61**: 7620–7623.
